# Design, synthesis, characterization, pharmacological evaluation and in silico ADMET and molecular docking and dynamics simulations of a novel series of *N-*substituted pyrazole from chalcone derivatives

**DOI:** 10.1038/s41598-026-38237-9

**Published:** 2026-03-01

**Authors:** Hend N. Hafez, Haleema Y. Otaif, Basmah H. Alshammari, Norah S. Alhebshe, Ahmed F. El-Sayed, Hebat-Allah S. Abbas, Reda A. Ammar

**Affiliations:** 1 Photochemistry Department, Chemical Industries Research Institute, National Research Centre, Dokki, 12622 Giza Egypt; 2 Collage of Science, Department of Physical Sciences, Chemistry Division, Jazan University, P.O. Box: 114, Jazan, 45142 Saudi Arabia; 3 Collage of Science, Department of Chemistry, University of Hail, P.O. Box: 2440, Hail, Saudi Arabia; 4 Microbial Genetics Department, Biotechnology Research Institute, National Research Centre, Dokki, 12622 Giza Egypt; 5 Egypt Center for Research and Regenerative Medicine (ECRRM), Cairo, Egypt; 6 Collage of Science, Department of Chemistry, Al Azhar University, Cairo, 11751 Egypt

**Keywords:** Chalcone, Pyrazole derivatives, Antibacterial, Anti-inflammatory, Ulcerogenic activities, Docking, ADMET and dynamic simulations, Biochemistry, Chemical biology, Chemistry, Computational biology and bioinformatics, Drug discovery

## Abstract

A novel series of *N*-pyrazolyl-thienopyrimidine and pyridopyrimidine hybrids was synthesized and characterized using spectroscopic methods. Compounds **4c**, **5c**, and **12** exhibited superior antibacterial potency compared to Levofloxacin, with molecular docking revealing strong binding to key bacterial targets (e.g., DNA gyrase, Neuraminidase). In-silico ADMET analysis confirmed favorable drug-like properties and low toxicity for **4c**, supported by stable molecular dynamics interactions. Additionally, **5c** and **12** demonstrated potent anti-inflammatory activity surpassing Celecoxib, with reduced ulcerogenicity. These findings introduce a promising new class of dual-action agents for antibacterial and anti-inflammatory drug development.

**Supplementary Information:**

The online version contains supplementary material available at 10.1038/s41598-026-38237-9.

## Introduction

Antibiotic resistance is one of the most serious global health concerns. It arises largely from the irrational and inappropriate use of antibacterial drugs, which accelerates the emergence of drug-resistant microbial infections. In response, the World Health Organization (WHO) issued a warning regarding the misuse of antibiotics, particularly during the COVID-19 pandemic^1^. Antimicrobial resistance presents a significant challenge to the effective prevention and treatment of a wide range of diseases caused by bacteria, viruses, and fungi. Heterocyclic ring systems are versatile structures found in numerous synthetic organic compounds and biologically active natural products^2,3^. These scaffolds can be incorporated into drug molecules to modulate their physicochemical, pharmacokinetic, and pharmacodynamic properties, as well as their toxicity profiles^4,5^.

**Fig. 1:**
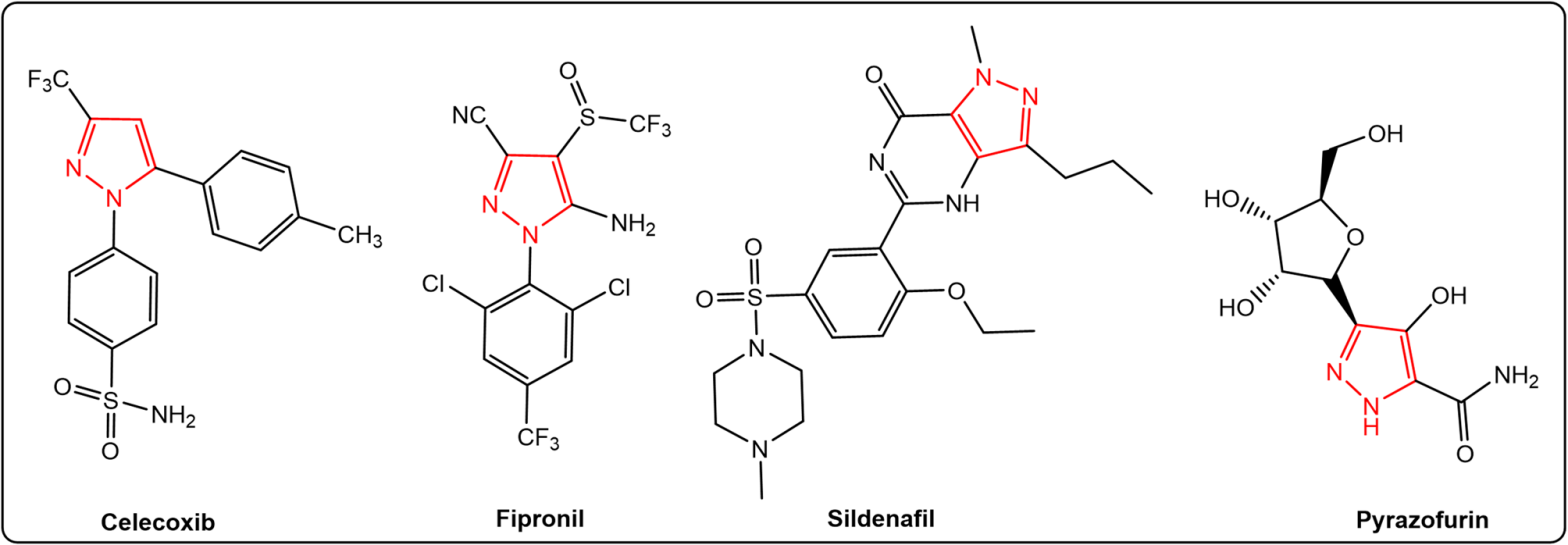
FDA approved drugs containing pyrazole scaffolds.

Among these, pyrazole-based scaffolds have attracted considerable interest due to their broad pharmacological potential and their relative resistance to antimicrobial resistance mechanisms^6,7^. Pyrazoles, a class of five-membered heterocycles containing two nitrogen atoms, have been widely investigated in the development of pharmaceutically active compounds^8^. Numerous studies have reported the diverse biological activities of pyrazole derivatives, including antidepressant^9^, anticonvulsant^10^, anticancer^11–13^, anti-inflammatory^14^, antibacterial^15^, and antihyperglycemic effects^16^. Several FDA-approved drugs featuring pyrazole scaffolds include Sildenafil^17^, Pyrazofurin^18^, Fipronil^19^, and Celecoxib^20^ (Fig. 1).

Inflammation can be triggered by various factors, including bacterial infections and biological agents. These factors play a pivotal role in the progression of conditions such as bacteremia and toxemia. Consequently, there is a pressing need for the synthesis and development of novel antibacterial and anti-inflammatory agents to ensure effective therapeutic interventions. Molecular docking is a widely employed computational technique used to predict the interactions between small-molecule therapeutic candidates and their target proteins. This method provides valuable insights into binding affinity and biological activity, making it a cornerstone of rational drug design. Given its biological and pharmacological significance, extensive research efforts have been directed toward improving the accuracy and reliability of docking algorithms^21^.

**Fig. 2:**
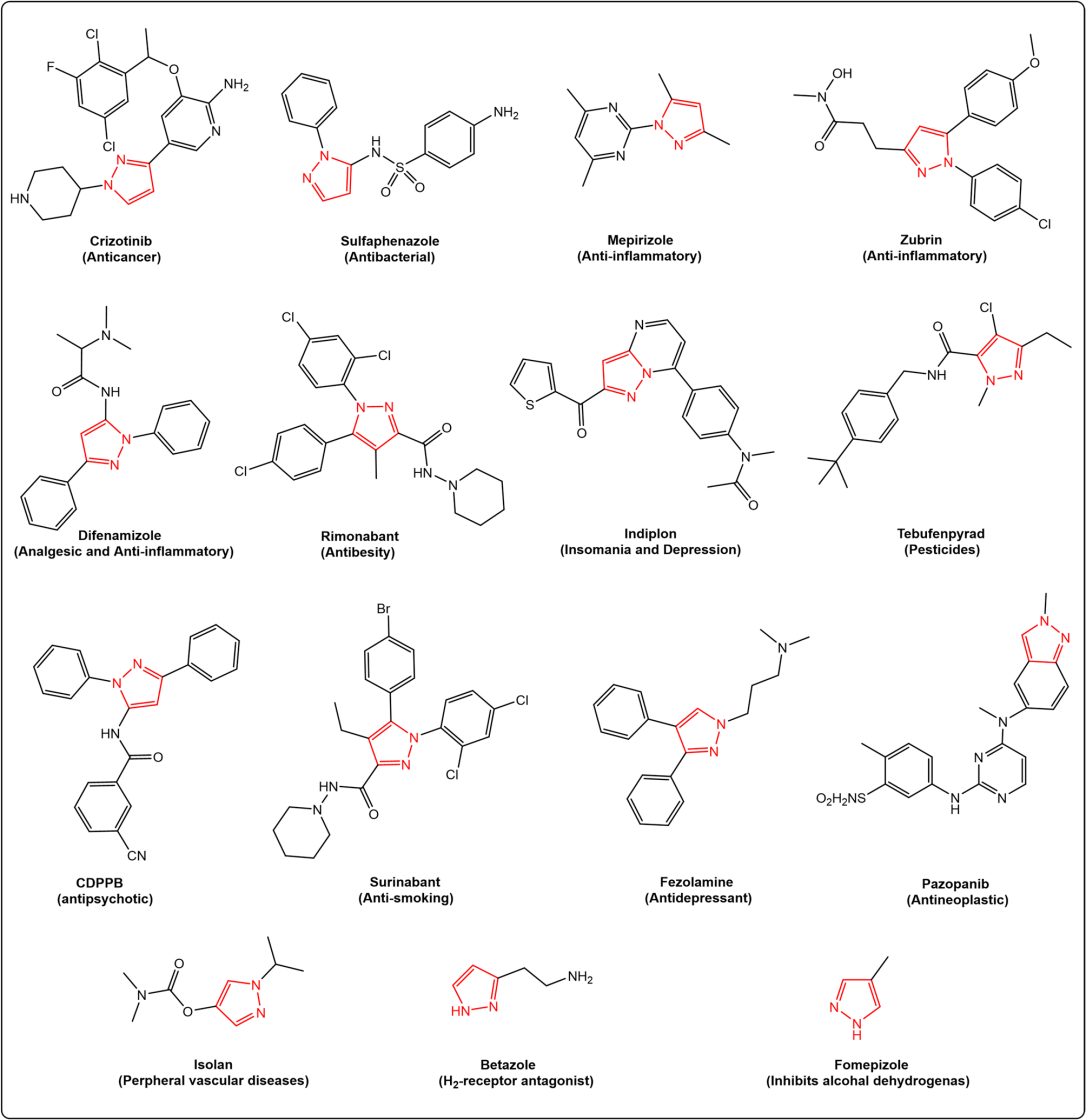
Some commercialized pyrazole-containing compounds.

The pyrazole core is one of the most vital heterocyclic scaffolds found in various marketed drugs^22^, including zubrin (anti-inflammatory), defenamizole (analgesic), sulfaphenazole (antibacterial), isolan (used for peripheral vascular diseases), indiplon (for insomnia and depression), fezolamine (antidepressant), and CDPPB (antipsychotic), among others (Fig. 2).

**Fig. 3:**
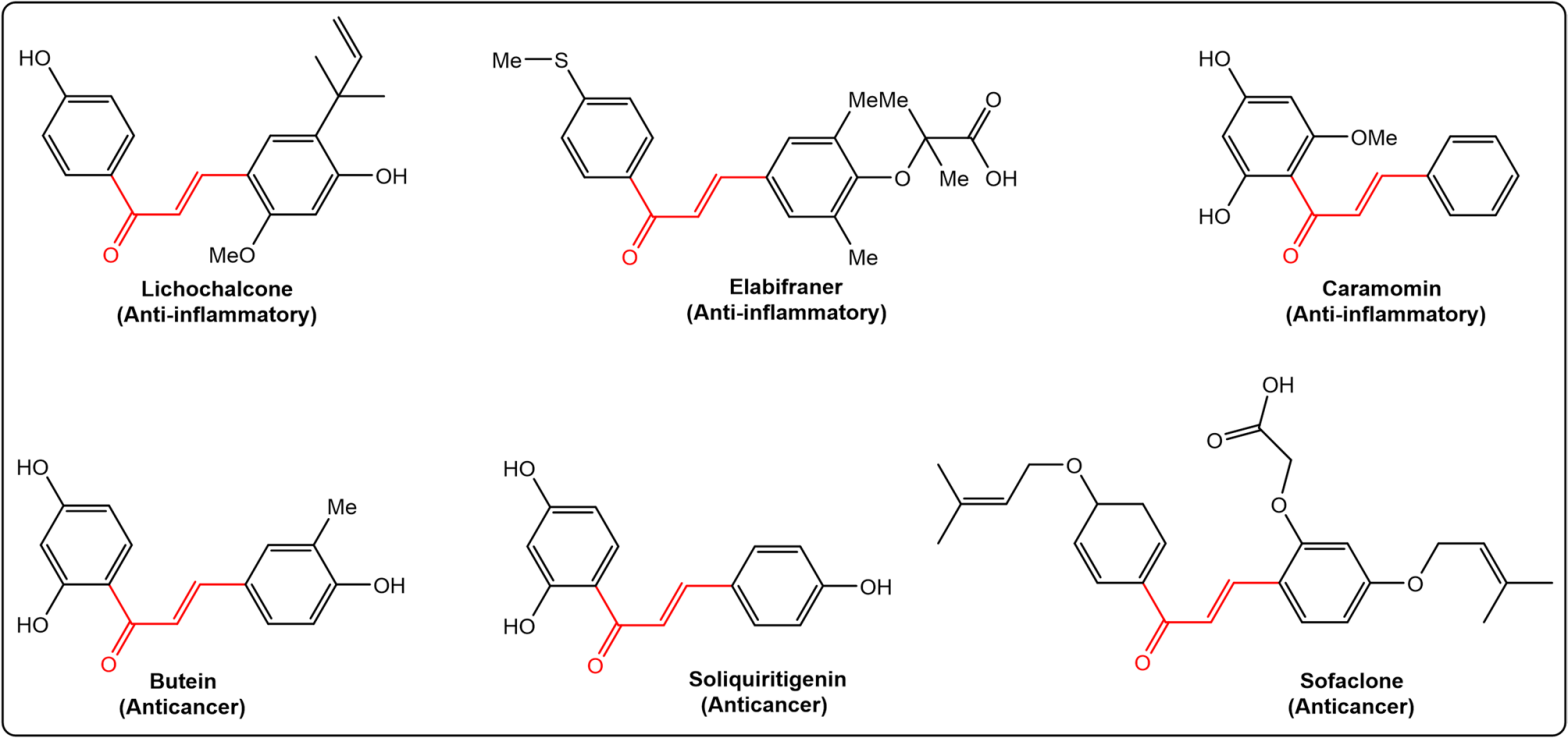
Chemical structures of selected commercial chalcone scaffold drugs.

In addition, chalcones - the aromatic ketones characterized by an *α*,*β*-unsaturated carbonyl system has emerged as promising scaffolds against the threat of antimicrobial resistance^23^. The chalcones chemistry remains a preoccupation among researchers due to the great number of replaceable hydrogens that permits a large number of derivatives and a wide range of favorable biological activities to be created^24^, including anti-inflammatory^25^, antifungal^26^, and anticancer^27^ effects (Fig. 3).

Chalcones represent important intermediates in the synthesis of various biologically active heterocyclic frameworks, including 1,4-diketones^28^, pyrazolines^29^, and benzothiazepines^30^. Among these, pyrazole–chalcone hybrids have attracted considerable attention due to their broad-spectrum antimicrobial properties, exhibiting potent inhibitory activity against diverse bacterial and fungal strains^31^. In 2020, Jadhava et al.^32^ reported the synthesis of several chalcone-linked pyrazole derivatives in excellent yields, which were subsequently evaluated for antifungal, anti-inflammatory, and antioxidant activities. Notably, some compounds demonstrated superior antifungal potency compared to the standard drug Nystatin against clinically relevant human pathogenic fungi. More recently, a novel series of pyrazole–chalcone analogs of Lonazolac^33,34^ was synthesized and investigated for anti-inflammatory potential. Within this series, one derivative emerged as a promising orally active anti-inflammatory candidate, exerting its effect through dual inhibition of 5-lipoxygenase (5-LOX) and inducible nitric oxide synthase (iNOS). Building upon these findings and in continuation of our previous investigations into pyrazole-based chemistry^35–44^. This research presents a detailed exploration of pyrazole nucleus derived from chalcones which has potent antibacterial agents, emphasizing their structural versatility, and therapeutic potential. With a modular backbone that supports diverse substitutions and heterocyclic extensions, their amphipathic nature and ability to bind critical bacterial enzymes offer an advantage in circumventing classical resistance mechanisms.

Accordingly, a new series of pyrazole derivatives functionalized with antipyrine, phenyl, thieno[2,3-*d*]pyrimidine, and pyrido[2,3-*d*]pyrimidine moieties have been synthesized. Their antibacterial properties were systematically assessed in comparison with levofloxacin as a reference drug, and their structure–activity relationships (SAR) were explored. Additionally, molecular docking analyses were conducted to elucidate the binding interactions of the synthesized compounds with relevant protein targets, thereby providing mechanistic insights into their antibacterial action^45–48^. Furthermore, the anti-inflammatory and ulcerogenic activities of these derivatives were also evaluated.

## Results and discussion

### Chemistry

**Fig. 4:**
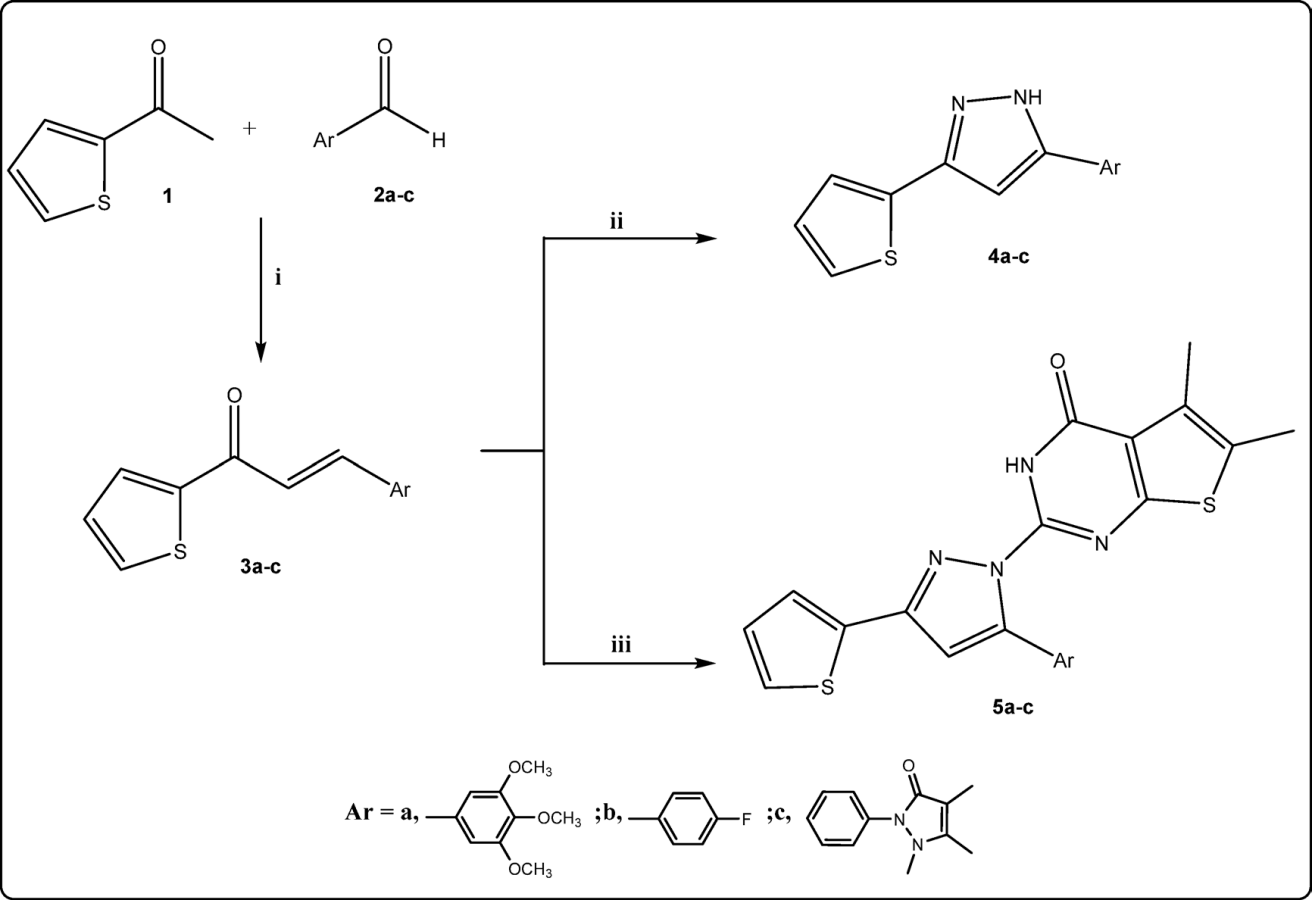
Reagents and conditions: (i): Ethanol, NaOH; (ii): H_2_N-NH_2_ (100%, 2mL)/ethanol, Δ; (iii): 2-hydrazineyl-5,6-dimethylthieno[2,3-*d*]pyrimidin-4(3*H*)-one, ethanol/pipridine, Δ.

The synthetic strategy of the target pyrazole derivatives was carried out *via* the cyclization of the key intermediate chalcone derivatives **3a-c** with hydrazine derivatives is illustrated in Fig. 4. Chalcone derivatives **3a-c** have been synthesized via Base-catalyzed Claisen-Schmidt condensation of 2-acetyl-thiophene **1** with various aldehydes **2a-c** namely (3,4,5-trimethoxybenzaldhyde, 4-fiuorobenzaldhyde and 4-antipyrine carboxaldehyde). Each reaction provided the desired product from high to moderate yield. Compound **3a-c** underwent cyclization in the treatment with hydrazine hydrate in ethanol to afford pyrazolyl derivative **4a-c**. Besides correct values of elemental analyses, IR and NMR spectra of compounds **4a-c** are in conformity with the assigned structures. IR spectrum of **4a** shows the absence of C=O group and the presence of broad absorption bands at *ν* 3382 cm^− 1^ of NH. ^1^H NMR spectra showed singlet NH at *δ* 11.21 ppm. The treatment of **3a-c** with 2-hydrazineyl-5,6-dimethylthieno[2,3-*d*]pyrimidin-4(3*H*)-one^49^ in ethanol/pipredine afforded substituted pyrazolyl-5,6-dimethylthieno[2,3-*d*]pyrimidin-4-one derivatives **5a-c** (Fig. 4).

**Fig. 5:**
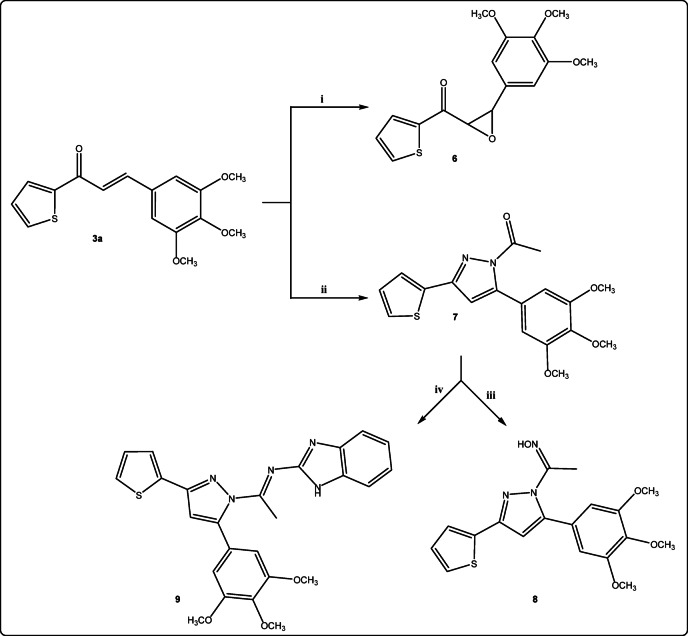
Reagents and conditions: (i): dry acetone, methanol, H_2_O_2_ (20%, 10mL), anhydrous NaOH, stir, 40 °C; (ii): H_2_N-NH_2_ (100%, 3mL)/AcOH, Δ; (iii): KOH, NH_2_OH.HCl, ethanol, Δ; (iv): 2-aminobenzimidiazole, ethanol/AcOH, Δ.

The structure of the new 5,6-dimethyl-2-(3-(thiophen-2-yl)-5-(3,4,5-trimethoxy-phenyl)-1*H*-pyrazol-1-yl)thieno[2,3-*d*]pyrimidin-4(3*H*)-one **5a** was confirmed by all spectroscopic data. The ^1^HNMR revealed that the corresponding two singlet signals of the 2CH_3_ appeared at *δ* 2.22 and 2.34 ppm and NH of pyrimidine moiety at δ 9.86 ppm and the ^13^C NMR spectrum indicated that the presence of C = O at *δ* 163.20 ppm. In basic medium, the treatment of chalcone **3a** with hydrogen peroxide afforded the corresponding epoxide derivative **6**. Cyclocondensation of chalcone **3a** with hydrazine hydrate in the presence of appropriate amount of acetic acid afforded 1-(3-(thiophen-2-yl)-5-(3,4,5-trimethoxyphenyl)-1*H*-pyrazol-1-yl)ethan-1-one **7** in good yield (Fig. 5). Compound **7** was transformed into pyrazolyl ethanone oxime derivatives **8** by the treatment with hydroxylamine hydrochloride in the presence of potassium hydroxide and ethanol as solvent. The IR spectra of compound **8** showed the absence of carbonyl group and the appearance of broad band at 3434 cm^− 1^ corresponding to OH and 1640 cm^− 1^ for C = N. On the other hand, the reaction of *N*-acetyl pyrazole derivative **7** with 2-aminobenzimidiazole afforded *N*-(1*H*-benzo[*d*]imidazol-2-yl)-1-(3-(thiophen-2-yl)-5-(3,4,5-trimethoxyphenyl)-1*H*-pyrazol-1-yl)ethan-1-imine **9** (Fig. 5).

**Fig. 6:**
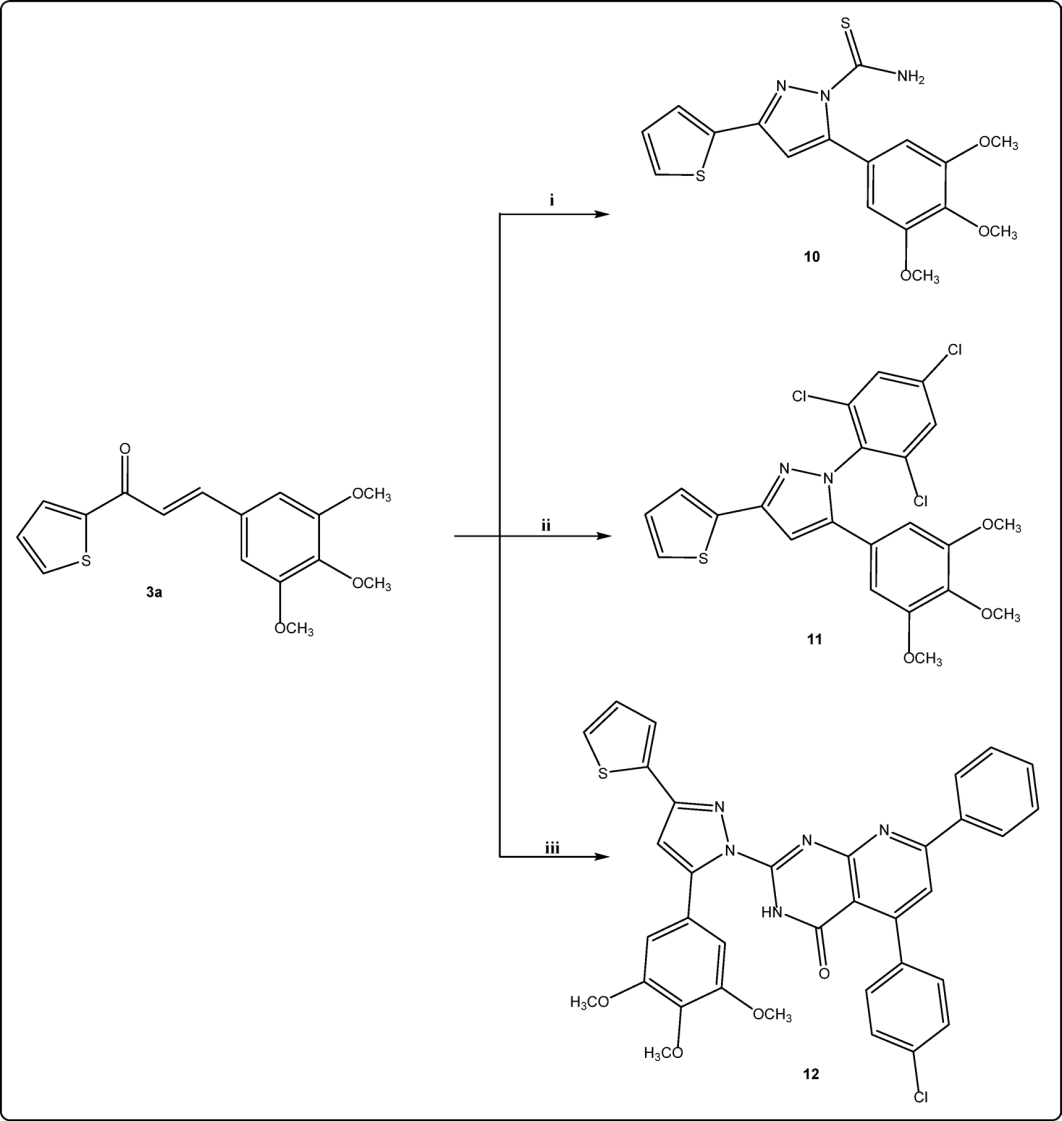
Reagents and conditions: (i): thiosemicarbazide, NaOH/ethanol, Δ; (ii): 2,4,6 trichlorophenyl hydrazine, K_2_CO_3_, ethanol, Δ; (iii): 5-(4-chlorophenyl)-2-hydrazineyl-7-phenylpyrido[2,3-*d*]pyrimidin-4(3*H*)-one, ethanol/pipridine, Δ.

The hydrazide moiety in selective compounds was exploited to synthesize some pyrazole derivatives through its reaction with it. The cyclocondensation of chalcone **3a** with hydrazide derivatives such as thiosemicarbazide in ethanolic sodium hydroxide afforded the corresponding 3-(thiophen-2-yl)-5-(3,4,5-trimethoxyphenyl)-1*H*-pyrazole-1-carbothioamide **10** and with 2,4,6 trichlorophenyl hydrazine using potassium carbonate as alkali afforded the corresponding *N*-trichlorophenyl pyrazolyl derivative **11** (Fig. 6).

Finally, chalcone **3a** was admixed with 5-(4-chlorophenyl)-2-hydrazineyl-7-phenylpyrido[2,3-*d*]pyrimidin-4(3*H*)-one^50^ in ethanol/piperidine to form the corresponding 5-(4-chlorophenyl)-7-phenyl-2-(3-(thiophen-2-yl)-5-(3,4,5-trimethoxy-phenyl)-1*H*-pyrazol-1-yl)pyrido[2,3-*d*]pyrimidin-4(3*H*)-one **12** (Fig. 6). All The title compounds have all been elucidated by ^1^H NMR, ^13^C NMR, and elemental analyses.

### Biological and pharmacological screening

#### Antibacterial activity

Pyrazole derivatives showed high antibacterial activity against various bacterial infections. Scientists used different approaches to demonstrate pyrazole as an antibacterial agent to find out the most active pyrazole derivatives in the present work^51^. The well-agar diffusion technique for the thirteen pyrazole compounds **4a-c**, **5a-c**, **6–12** showed variable antibacterial activity against different types of bacteria strains (Table 1). Among the compounds of the pyrazole tested, pyrazole derivatives **4c**, **5c** and **12** showed noticeable potent antibacterial with MIC ranges from (5–8 µmol L^− 1^) greater than or equipotent activity to the reference antibiotic (Levofloxacin) with MIC ranges from (6–10 µmol L^− 1^). On the other hand, compounds **4b**, **9** and **11** have good activities against all bacteria with MIC ranges from (8–14 µmol L^− 1^), but compound **5b** has good activity against gram positive with MIC ranges from (9–12 µmol L^− 1^), and nearly active as Levofloxacin against gram negative bacteria with MIC ranges from (8–12 µmol L^− 1^). While compounds **4a**, **5a**, **6**, **7**, **8** and **10** exhibited moderate activities toward all bacteria strains.

**Table 1:** Minimal inhibitory concentration (MIC, *µmolL*^*− 1*^) of the newly synthesized compounds.

Compound	Microorganisms					
	Gram +ve bacteria			Gram - ve bacteria		
	*S. pneumonia*	*S. aureus*	*B. subtilis*	*E. coli*	*K. pneumonia*	*S. typhimurium*
**4a**	16	12	12	14	12	15
**4b**	12	10	10	14	10	10
**4c**	8	8	8	8	6	6
**5a**	16	14	20	15	12	14
**5b**	12	10	9	12	8	10
**5c**	7	8	6	8	5	6
**6**	15	13	12	15	11	14
**7**	16	11	12	14	11	13
**8**	14	12	13	15	13	14
**9**	11	9	9	12	10	10
**10**	14	12	12	14	13	12
**11**	10	8	9	13	10	11
**12**	5	4	5	7	6	8
**Levofloxacin**	8	6	6	10	6	8

#### Anti-inflammatory activity

**Fig. 7:**
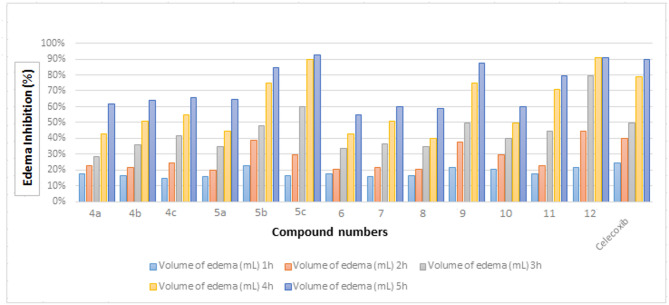
The percentage of edema inhibition produced by tested compounds at various time intervals of carrageenan-induced rat paw edema.

The study evaluated the anti-inflammatory activity of the newly synthesized derivatives. Compounds **4a-c**, **5a-c**, **6–12** were also evaluated for their anti-inflammatory activity and the results are shown in Table 2; Fig. 7. The obtained pharmacological results revealed that most of the test compounds showed significant anti-inflammatory activity with inhibition ranging from 55% to 93%. Compounds **5c** (93%) and **12** (91%) have the strongest anti-inflammatory activity compared to the standard drug celecoxib (90%). In addition, compound **9** exhibited anti-inflammatory activity (88%) as near as the standard (90%). Furthermore, compound **5b** exhibited high anti-inflammatory activity (85%) but still less than the standard drug celecoxib (90%). However, compounds **4a-c**, **5a** and **11** exhibited good activity ranging from (62–80%) and, the remaining compounds **7**, **8** and **10** moderate anti-inflammatory activity while, compound **6** showed low activity (55%).

**Table 2:** Anti-inflammatory activity of the newly synthesized compounds.

Compound^a^	Volume of edema (mL) ^b^				
	1 h	2 h	3 h	4 h	5 h
**4a**	0.68 ± 0.01(18%)	0.66 ± 0.01(23%)	0.64 ± 0.01(29%)	0.54 ± 0.01(43%)	0.42 ± 0.0152(62%)
**4b**	0.67 ± 0.005(17%)	0.65 ± 0.01(22%)	0.54 ± 0.0115^*^ (36%)	0.43 ± 0.01 (51%)	0.48 ± 0.011^*^ (64%)
**4c**	0.69 ± 0.015 (15%)	0.65 ± 0.006 (25%)	0.67 ± 0.01(42%)	0.44 ± 0.01(55%)	0.37 ± 0.0057(66%)
**5a**	0.67 ± 0.005^**^(16%)	0.65 ± 0.005^**^(20%)	0.60 ± 0.005^**^(35%)	0.54 ± 0.024^**^(45%)	0.47 ± 0.005^**^ (65%)
**5b**	0.62 ± 0.01(23%)	0.57 ± 0.005(39%)	0.56 ± 0.01(48%)	0.35 ± 0.155(75%)	0.32 ± 0.01 (85%)
**5c**	0.66 ± 0.005 (17%)	0.65 ± 0.0055(30%)	0.48 ± 0.0054(60%)	0.34 ± 0.0051(90%)	0.34 ± 0.0173 (93%)
**6**	0.64 ± 0.01(18%)	0.62 ± 0.0052(21%)	0.56 ± 0.0115(34%)	0.40 ± 0.01 (43%)	0.38 ± 0.0115(55%)
**7**	0.65 ± 0.01(16%)	0.60 ± 0.0052(22%)	0.58 ± 0.0115 (37%)	0.46 ± 0.0052(51%)	0.48 ± 0.0152 (60%)
**8**	0.66 ± 0.005 (17%)	0.64 ± 0.01(21%)	0.56 ± 0.0115^*^ (35%)	0.42 ± 0.01 (40%)	0.40 ± 0.0154(59%)
**9**	0.66 ± 0.01(22%)	0.58 ± 0.01(38%)	0.58 ± 0.0115^*^ (50%)	0.36 ± 0.0264(75%)	0.37 ± 0.0152 (88%)
**10**	0.64 ± 0.0075(21%)	0.53 ± 0.023(30%)	0.60 ± 0.0173(40%)	0.48 ± 0.0057(50%)	0.46 ± 0.0115 (60%)
**11**	0.64 ± 0.01(18%)	0.66 ± 0.0054 (23%)	0.58 ± 0.0115 (45%)	0.40 ± 0.011 (71%)	0.36 ± 0.0152 (80%)
**12**	0.63 ± 0.0055(22%)	0.60 ± 0.0173(45%)	0.38 ± 0.0115 (80%)	0.34 ± 0.0057(91%)	0.35 ± 0.0057 (91%)
**Control**	0.80 ± 0.02	0.86 ± 0.002	0.91 ± 0.001	0.95 ± 0.02	0.97 ± 0.02
**Celecoxib**	0.65 ± 0.01(25%)	0.59 ± 0.005(40%)	0.56 ± 0.01(50%)	0.38 ± 0.153(79%)	0.34 ± 0.01 (90%)

^a^Dose levels: test compounds (50 mg/kg b.w), **celecoxib** (50 mg/kg b.w).

^b^*n* = 6, values are expressed as mean ± SEM and analysed by ANOVA.

^c^Values in parentheses (percentage anti-inflammatory activity, AI%).

^*^Means significant difference with celecoxib at *p* < 0.05.

^**^Means significant difference with celecoxib at *p* < 0.01.

#### Ulcerogenic liability study

The ulcerogenic activity was screened on the compounds, which exhibited high anti-inflammatory activity **5b**, **5c**, **9**, **10**,** 11** and **12** Table 3. Compounds **12** and **9** exhibited high reduction in ulcerogenic activity was (0.4 ± 0.0) also, compounds **5c** and **5b** exhibited high reduction in ulcerogenic activity was (0.6 ± 0.0), (0.8 ± 0.2) respectively. Finally, compounds **10** and **11** showed moderate severity indexes.

**Table 3:** Ulcerogenic activity.

Compound	Dose (mg kg^− 1^, 1% CMC)	Ulcer index^a^
Control		0.0 ± 0.0^b^
**5b**	200	0.8 ± 0.2^b^
**5c**	200	0.6 ± 0.0^b^
**9**	200	0.4 ± 0.0^b^
**10**	200	0.6 ± 0.2^c^
**11**	200	0.5 ± 0.2^c^
**12**	200	0.4 ± 0.0^b^
**Celecoxib**	200	0.9 ± 0.2

Each value represents the mean ± SD ^a^ *n* = 6, ^b^ *P* < 0.0001 and ^c^ *P* < 0.001.

### Computational analysis

#### Docking and molecular interaction of synthesized compounds

**Fig. 8:**
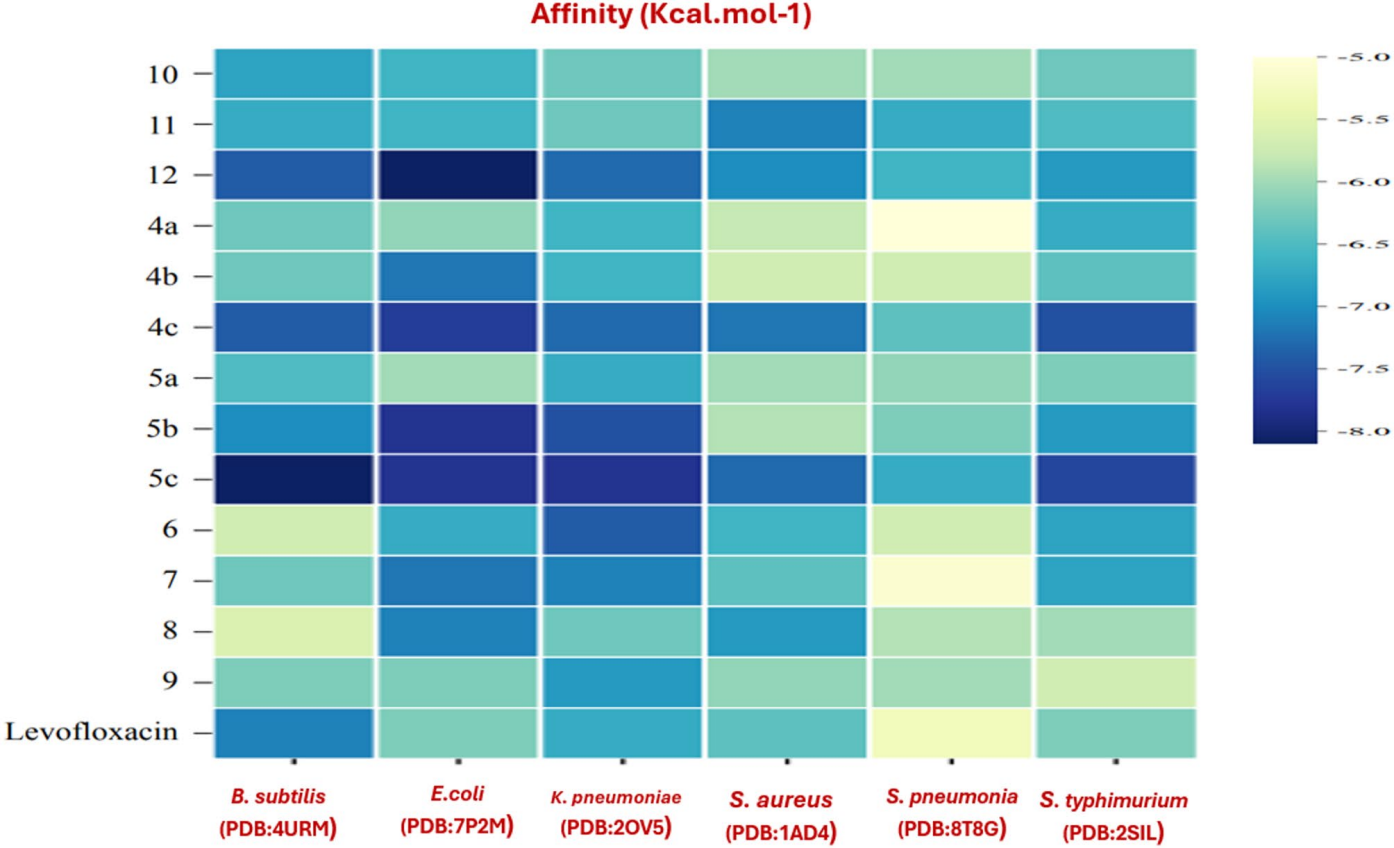
Heatmap of binding affinity of compounds with targets of proteins.

Molecular docking studies were performed to investigate the interactions between the synthesized compounds and protein targets associated with antimicrobial activity, aiming to evaluate their efficacy. The results of the docking experiments, presented in Table S1 and Fig. 8, highlight the binding affinities of the compounds with four antibacterial receptors. The binding affinity data indicates varying inhibitory capabilities of the compounds **4c**,** 5c**,** 12**,** and 5b** against bacterial targets, withstanding out as broad-spectrum candidates.

#### Docking and molecular interaction with gyraseb of B. subtilis (PDB: ID 4URM)

**Fig. 9:**
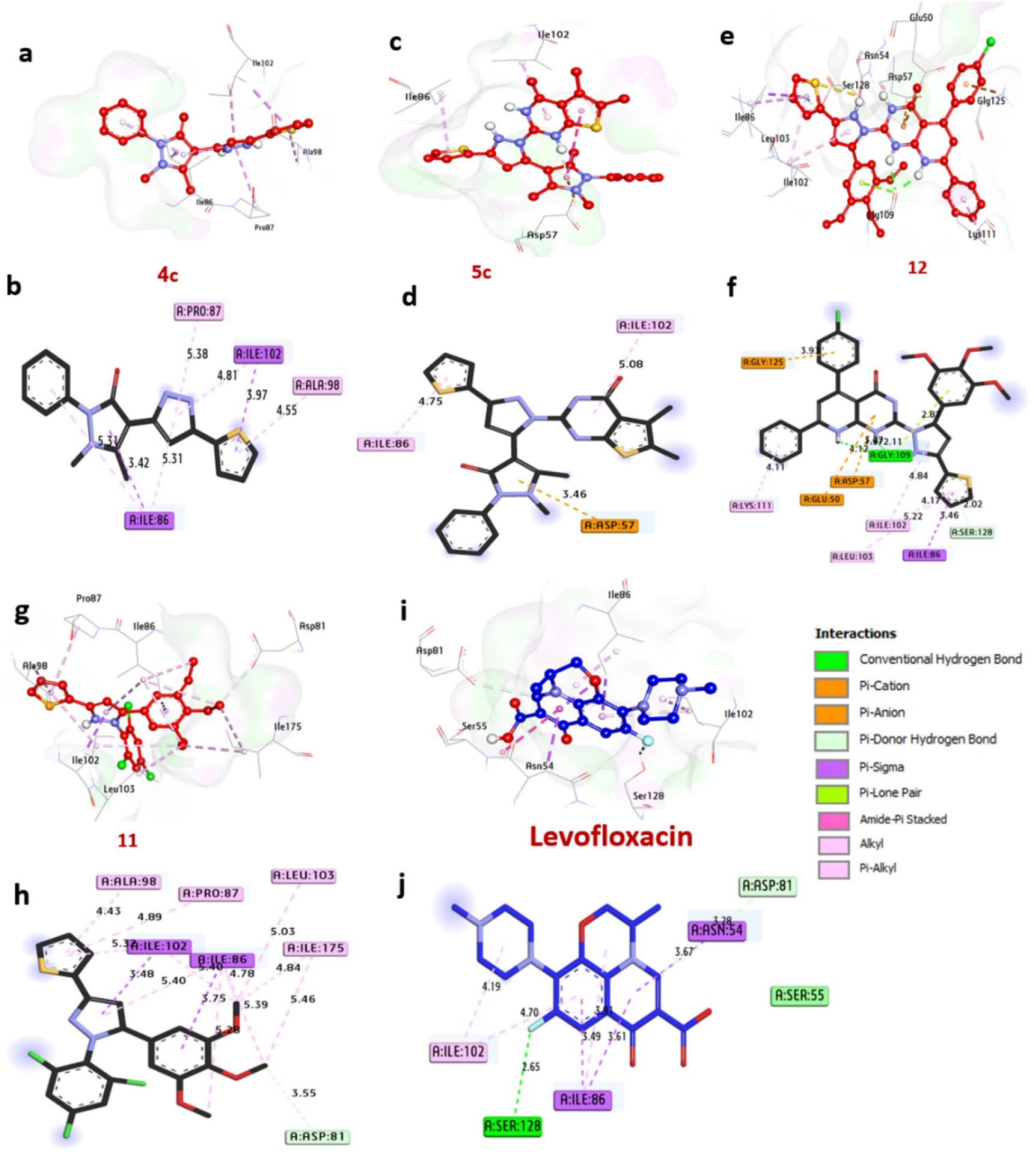
3D representations of compound conformations at the binding pocket of GyraseB of B. subtilis (PDB: ID 4URM): (**a**,**b**) **4c**, (**c**,**d**) **5c**, (**e**,**f**) **12**, (**g**,**h**) **5b**, (**I**,**j**) **Levofloxacin**.

Gyrase B in *Bacillus subtilis* **4URM** plays a pivotal role in genome maintenance, facilitating essential processes such as DNA replication, transcription, and adaptation to environmental stress. Its ATP-dependent enzymatic activity and evolutionary conservation across bacterial species make it a prime target for antibiotic development. Molecular docking analyses have identified compounds **4c**,** 5c**,** 12**, and **5b** as promising candidates, exhibiting binding energies of -7.40, -8.10, -7.40 and − 8.10 kcal/mol, respectively, approaching the binding affinity of the reference antibiotic Levofloxacin (-7.10 kcal/mol) (Table S2, Fig. 9). Among these, compound **12** uniquely forms a hydrogen bond with the catalytic site residue Gly109, a critical interaction for inhibitory activity. Additional stabilizing interactions include hydrophobic (Pi-alkyl) bonds with Pro87, Ala98, Ile86, Lys111, Leu103, and Ile102; carbon-hydrogen bonds with Asp81 and Ser128; and diverse interactions such as Pi-sigma bonds with Ile86 and Ile102, and Pi-cation bonds with Gly125, Glu50, and Asp57. Key catalytic residues, including Gly109, Ile86, Ile102, and Ala98, were found to significantly enhance ligand binding. Collectively, these findings highlight the potential of compounds **4c**,** 5c**,** 12**,** and 5b** as inhibitors of *B. subtilis* Gyrase-B. These results corroborate recent studies employing molecular docking to explore antibacterial mechanisms. For instance, investigated compound-protein interactions to elucidate anti-bacterial properties^52^, while focused on small-molecule inhibition of B. subtilis Gyrase B^53^.

#### Docking and molecular interaction studies with DNA gyrase of E.coli 7P2M

**Fig. 10:**
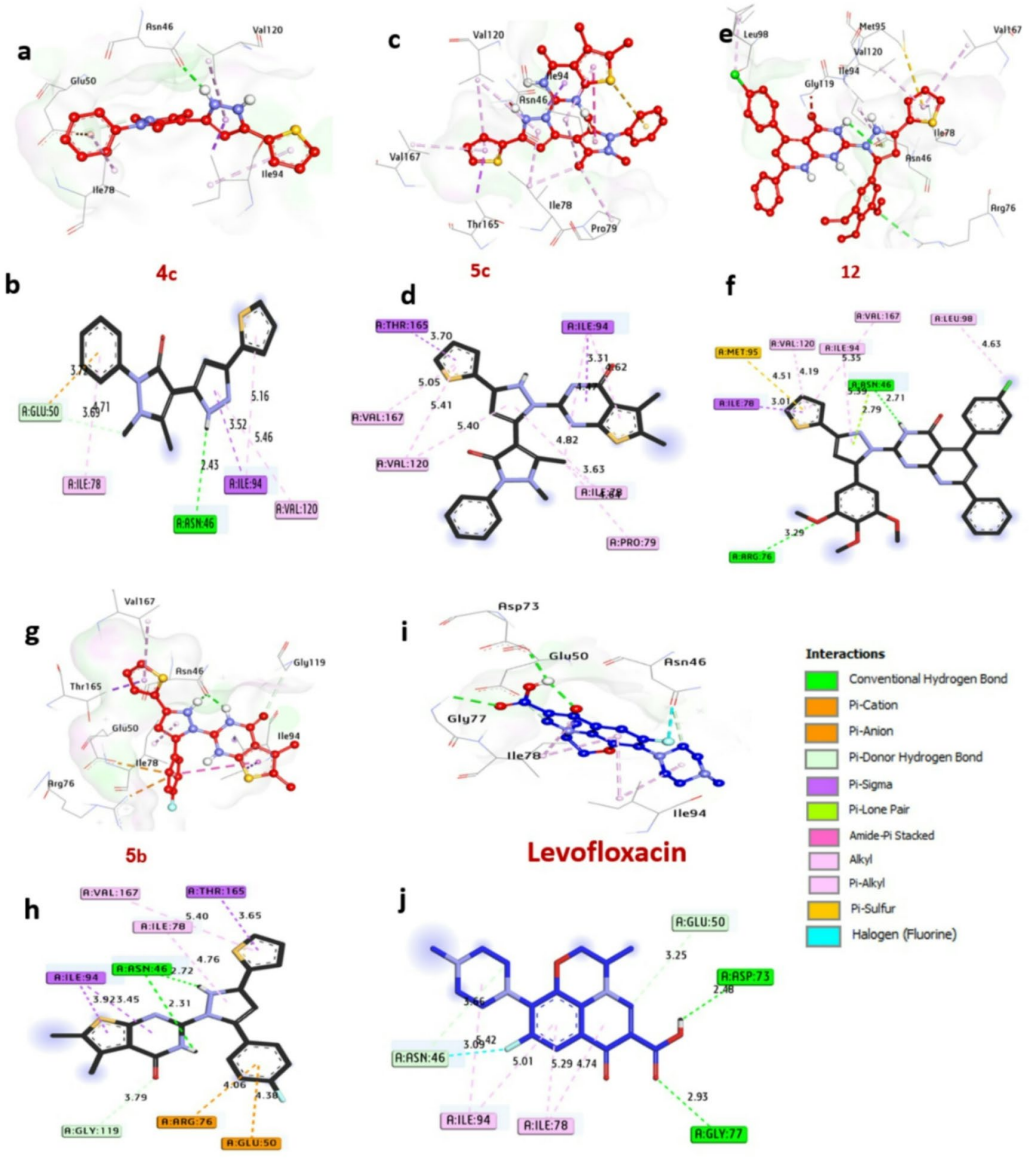
3D representations of compounds conformations at the binding pocket of GyrB24 of *E. coli* (PDB: ID 7P2M) : (**a**,**b**) **4c**, (**c**,**d**) **5c**, (**e**,**f**) **12**, (**g**,**h**) **5b**, (**i**,**j**) **Levofloxacin**.

DNA gyrase, a vital enzyme in bacteria such as Escherichia coli **7P2M**, is essential for DNA replication and transcription by managing topological stress during these processes. Molecular docking analysis **(**Table S3, Fig. 10**)** reveals that compounds **4c**,** 5c**,** 12**, and **5b** exhibit the strongest inhibitory potential, with binding affinities of − 7.70, − 7.80, − 8.10, and − 7.80 kcal/mol, respectively, outperforming the control drug Levofloxacin (-6.20 kcal/mol). Notably, compounds **4c**,** 12**, and **5b** form hydrogen bonds with catalytic residues Asn46 and Arg76, which are critical for stabilizing ligand-enzyme interactions. Hydrophobic interactions further enhance binding, including alkyl bonds with Val120, Ile78, Ile94, Val167, Pro79, and Leu98; sulfur interactions with Met95; carbon-hydrogen bonds with Glu50 and Gly119; Pi-sigma bonds with Ile94, Ile78, and Thr165; and Pi-cation bonds with Glu50 and Arg76. Key catalytic residues Asn46, Arg76, and Ile94 significantly contribute to the enhanced binding affinity observed in these compounds. Collectively, these findings highlight the potential of compounds **4c**,** 5c**,** 12**,** and 5b** as inhibitors of DNA Gyrase of *E.coli*. These findings align with the work of^54^ Sroor et al. (2024), who combined antimicrobial assays against *E. coli* with in-silico docking to validate the inhibition of DNA gyrase by small-molecule ligands, reinforcing the enzyme’s role as a therapeutic target.

#### Docking and interaction of KPC-2 carbapenemase of K. pneumoniae 2OV5

**Fig. 11:**
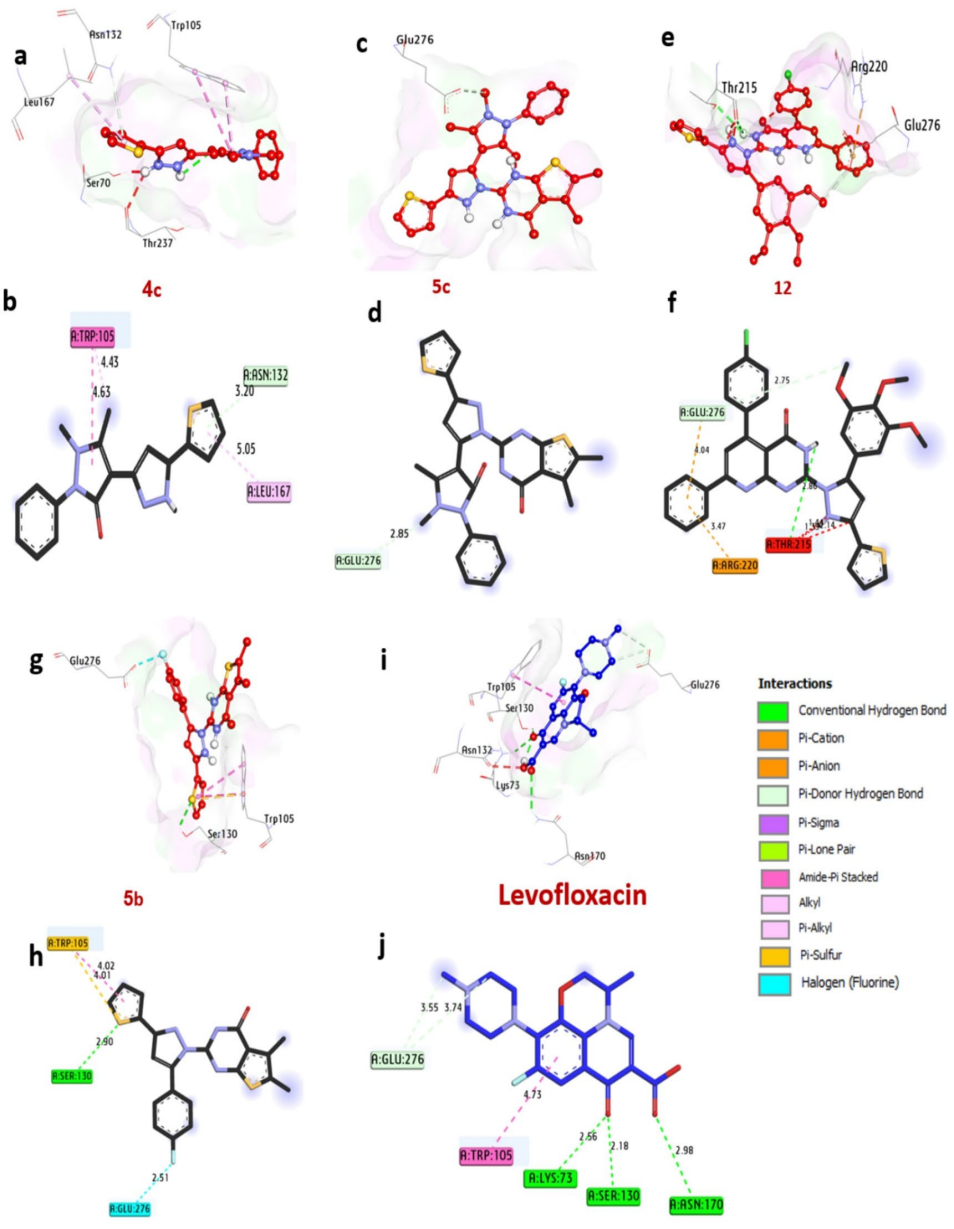
3D representations of compounds at the binding pocket of KPC-2 carbapenemase of *K. pneumoniae* (PDB: ID 2OV5): (**a**,**b**) **4c**, (**c**,**d**) **5c**, (**e**,**f**) **12**, (**g**,**h**) **5b**, (**i**,**j**) **Levofloxacin**.

KPC-2 carbapenemase, a key resistance determinant in *K. pneumoniae*, compromises the efficacy of carbapenem antibiotics, which are critical for treating multidrug-resistant infections. Molecular docking analysis (Table S4, Fig. 11) highlights compounds **4c**,** 5c**,** 12**,** and 5b** as potent inhibitors, exhibiting binding affinities of -7.30, -7.80, -7.30, and − 7.50 kcal/mol, respectively, outperforming the control drug Levofloxacin (-6.70 kcal/mol). Notably, compounds **12** and **5b** form hydrogen bonds with Ser130 and Thr215, residues essential for the enzyme’s catalytic activity. Additional stabilizing interactions include alkyl bonds with Trp105 and Leu167, halogen interactions with Glu276, sulfur bonds with Trp105, Pi-sigma bonds with Thr216, carbon-hydrogen bonds with Asn132 and Glu276, and Pi-cation bonds with Glu276 and Arg220. The catalytic residues Thr215, Thr235, Glu276, and Trp105 are central to enhancing ligand binding, underscoring their role in the enzyme’s functional architecture. These computational insights, supported by in vitro antibacterial activity data, position these compounds as promising candidates for inhibiting KPC-2 carbapenemase **2OV5**. The findings align with^55^ Mukhtar et al. (2024), who validated analogous compounds against *K. pneumoniae*, reinforcing the synergy between computational predictions and experimental validation in antibiotic development.

#### Docking and interaction with dihydropteroate synthase of S. aureus 1AD4

**Fig. 12:**
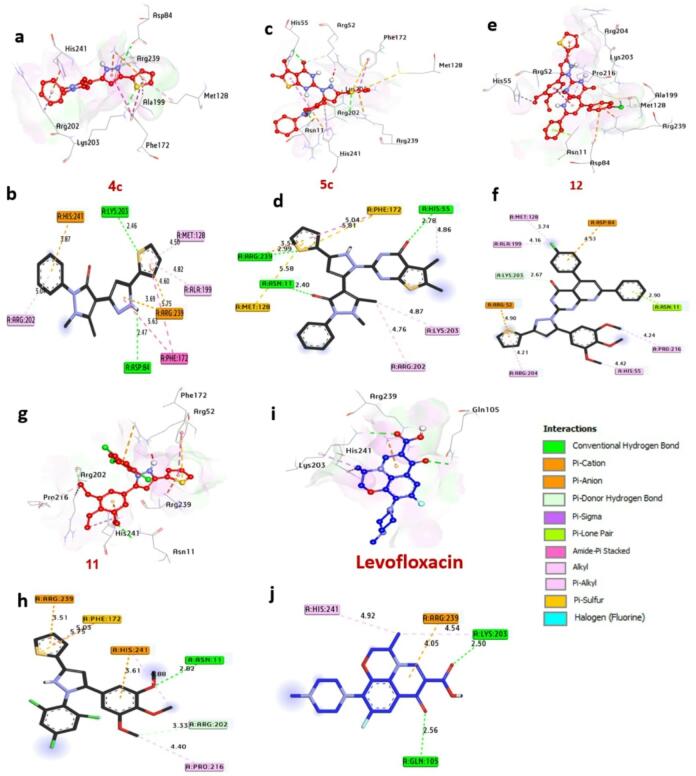
3D representations of compound conformations at the binding pocket of dihydropteroate synthase of *S. aureus* (PDB: ID 1AD4): (**a**,**b**) **4c**, (**c**,**d**) **5c**, (**e**,**f**) **12**, (**g**,**h**) **5b**, (**i**,**j**) **Levofloxacin**.

Dihydropteroate synthase (DHPS), a key enzyme in the bacterial folate synthesis pathway, is a validated target for antimicrobial agents, as its inhibition disrupts nucleotide synthesis and halts bacterial proliferation. Computational docking analysis (Table S5, Fig. 12) identifies compounds **4c**,** 5c**,** 12**, and **11** as potent DHPS inhibitors, with binding energies of -7.20, -7.30, -7.00 and − 7.10 kcal/mol, respectively, significantly surpassing Levofloxacin (-6.40 kcal/mol). These compounds engage in hydrogen bonding with catalytic residues Lys203, Asp84, His55, Arg239, and Asn11, critical for substrate coordination. Hydrophobic interactions further stabilize binding, including alkyl bonds with Ala199, Met128, Arg202, His55, Lys203, Arg204, Pro216, and His241; Pi-Pi stacked interactions with Phe172; Pi-cation bonds with Arg239, His241, Asp84, and Arg52; Pi-sulfur bonds with Phe172 and Met128; and carbon-hydrogen bonds with Arg202 and Lys203. Residues Lys203, Asn11, and His241 in the catalytic site are pivotal for enhancing ligand affinity. Collectively, these interactions suggest that the compounds exert antibacterial effects by blocking DHPS activity in Staphylococcus aureus. These findings align with^45^ Khidre et al. (2023), who employed molecular docking to identify small-molecule inhibitors of S. aureus DHPS, reinforcing the enzyme’s role as a therapeutic target.

#### Docking and interaction with Streptococcus pneumonia sortase A (spySrtA) (8T8G)

**Fig. 13:**
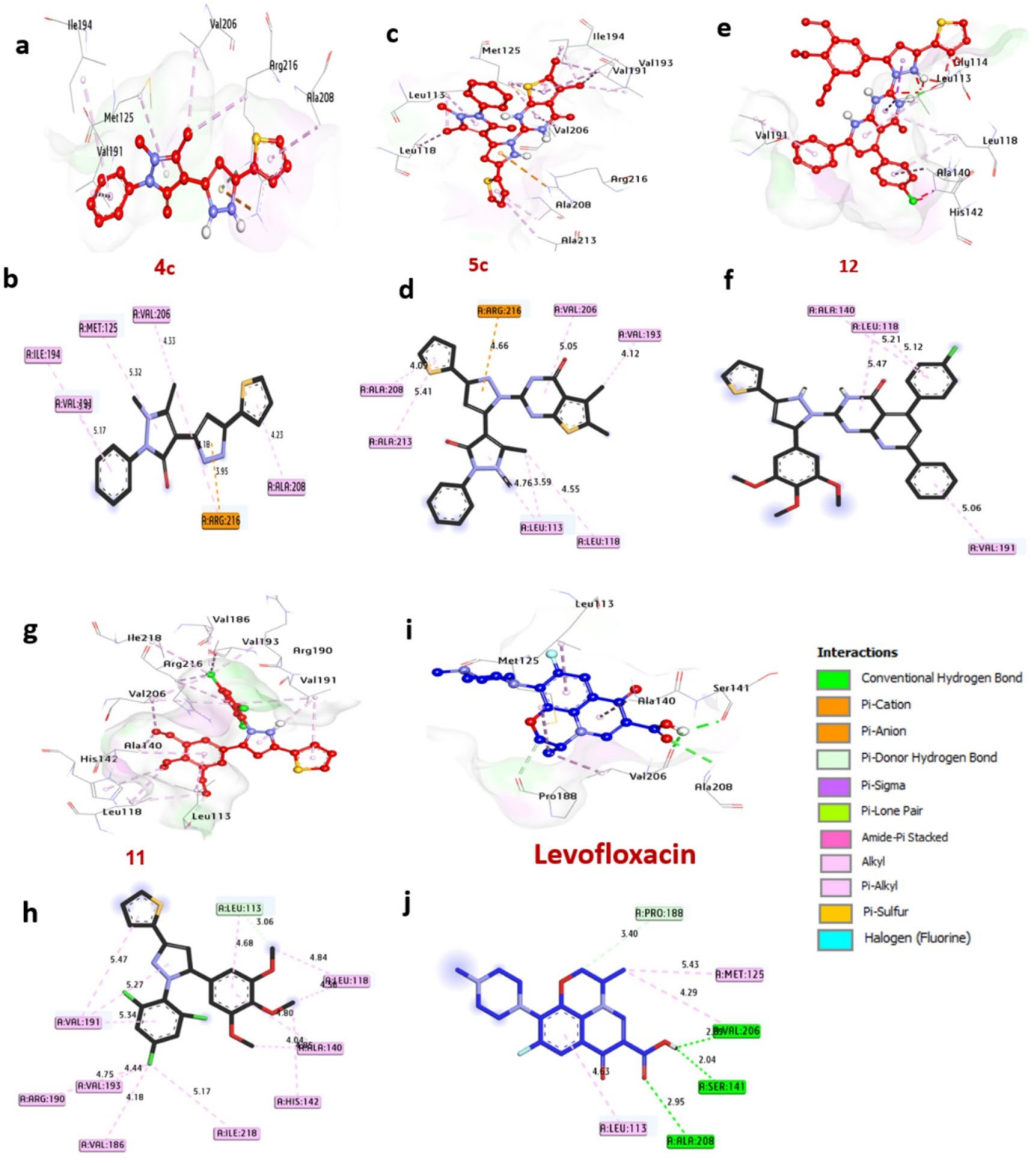
3D representations of compounds conformations at the binding pocket of Streptococcus pneumonia Sortase A (spySrtA) (8T8G): (**a**,**b**) **4c**, (**c**,**d**) **5c**, (**e**,**f**) **12**, (**g**,**h**) **5b**, (**i**,**j**) **Levofloxacin**.

Sortase A, a virulence factor critical to Streptococcus pneumoniae pathogenicity, facilitates the anchoring of surface proteins essential for host colonization, immune evasion, and nutrient acquisition. Targeting this enzyme represents a strategic avenue for developing anti-infectives that attenuate bacterial virulence. Molecular docking analysis (Table S6, Fig. 13) identifies compounds **4c**,** 5c**,** 12**, and **11** as potential Sortase A inhibitors, with binding affinities of -6.40, -6.70, -6.60, and − 6.70 kcal/mol, respectively, surpassing the reference drug Levofloxacin (-6.30 kcal/mol). While none of the compounds form hydrogen bonds with catalytic residues, they stabilize binding through hydrophobic interactions: alkyl bonds with Arg216, Ala208, Val206, Met125, Ile194, Val191, Val193, Ala213, Leu113, Leu118, Ala140, His142, Ile218, Val186, and Arg190; a Pi-cation bond with Arg216; and carbon-hydrogen bonds with Leu113. Key residues Leu118, Ala208, and Val191 within the catalytic pocket enhance ligand affinity, suggesting these compounds disrupt Sortase A’s substrate recognition or catalytic activity. Collectively, these interactions suggest that the compounds exert antibacterial effects by blocking Sortase A activity in Streptococcus pneumonia. These findings align with^45^ Jawhari et al. (2023), who identified small molecules with strong Sortase A interactions, and^48^ Sroor et al. (2024), who leveraged docking to design novel inhibitors, underscoring the enzyme’s tractability as a therapeutic target.

#### Docking and interaction with neuraminidase of S. typhimurium 2SIL

**Fig. 14:**
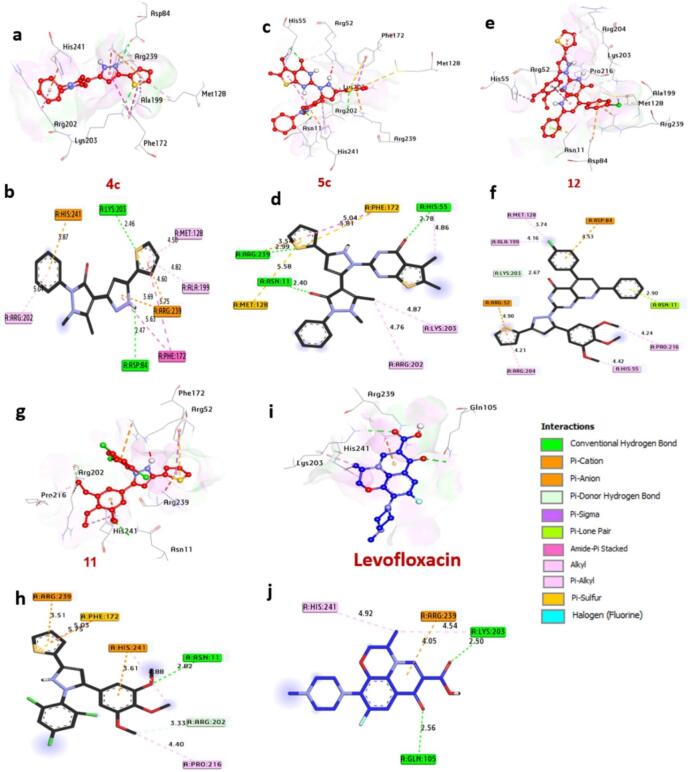
3D representations of compound conformations at the binding pocket of dihydropteroate synthase of *S. aureus* (PDB: ID 1AD4): (**a**,**b**) **4c**, (**c**,**d**) **5c**, (**e**,**f**) **12**, (**g**,**h**) **5b**, (**i**,**j**) **Levofloxacin**.

Neuraminidase in Salmonella typhimurium is a pivotal virulent enzyme, enabling bacterial survival through nutrient acquisition, host tissue invasion, and immune evasion. Targeting this enzyme offers a strategic approach to attenuate pathogenicity. Molecular docking analysis (Table S7, Fig. 14) identifies compounds **4c**,** 5c**,** 12**, and **5b** as potent Neuraminidase inhibitors, with binding affinities of -7.50, -7.60, -6.90, and − 6.90 kcal/mol, respectively, outperforming Levofloxacin (-6.20 kcal/mol). Notably, compounds **4c**, **5c**, and **12** form hydrogen bonds with catalytic residues Trp128, Thr127, Gln63, and Arg246, which are critical for substrate recognition and enzymatic activity. Additional stabilizing interactions include alkyl bonds with Met99, Trp128, Leu205, Val196, and Leu175; sulfur bonds with Met99 and Tyr307; amide-pi stacking with Trp128; Pi-sigma bonds with Leu205; Pi-cation bonds with Glu231, Arg246, Asp62, and Asp100; and carbon-hydrogen bonds with Glu129, Glu231, and Asp100. Catalytic residues Arg309, Arg246, Asp100, and Thr127 significantly enhance ligand binding, underscoring their role in the enzyme’s functional architecture. These findings align with^47^ Khaled et al. (2023), who similarly employed molecular docking to identify neuraminidase inhibitors, reinforcing the potential of these compounds as anti-bacterial for *S. typhimurium* agents.

### In silico pharmacokinetics ADME prediction of synthesized compounds

**Fig. 15:**
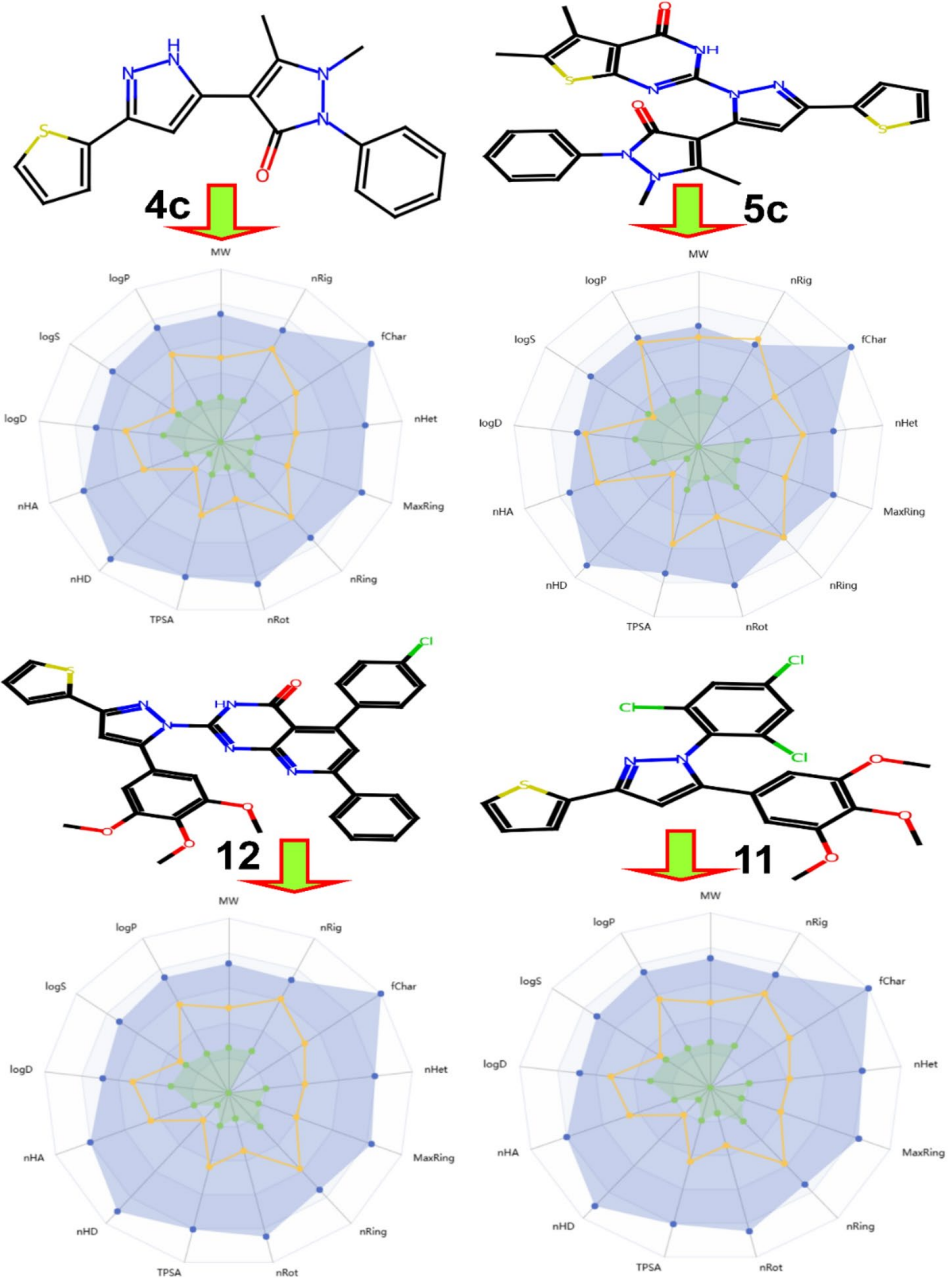
Oral bio-availability graph for compounds.

The results presented in (Table S8 and Fig. 15) presents pharmacokinetic, physicochemical, toxicity, and oral bioavailability properties of four compounds **4c**,** 5c**,** 12**, and **11**. For physicochemical properties, compound **4c** has a molecular weight (MW) of 336.10 g/mol, a logP of 1.56, and a topological polar surface area (TPSA) of 80.47 Å², while **5c** shows a higher MW of 514.0 g/mol, a logP of 3.76, and a TPSA of 139.3 Å². Compound **12** has an MW of 647.14 g/mol, a logP of 7.69, and a TPSA of 128.1 Å², and compound **11** has the highest MW at 494.0 g/mol, a logP of 5.96, and a TPSA of 73.75 Å². These values indicate that **4c** is the smallest and least lipophilic, while **12** is the largest and most lipophilic, which could impact their solubility and membrane permeability. For solubility (logS), **4c** is moderately soluble at -2.74, whereas **5c**, **12**, and **11** show poorer solubility with logS values of -6.00, -10.4, and − 7.09, respectively, suggesting potential challenges in dissolution for the latter three compounds. For absorption, all compounds exhibit (HIA), but **4c** has the highest Caco-2 permeability at -4.77, indicating better intestinal absorption compared to **5c** (-4.76), 12 (-4.90), and 11 (-4.96). For distribution, **4c** has the lowest volume of distribution (VDss) at -1.931 log L/kg, while **12** has the highest at -8.680 log L/kg, suggesting **12** may have more extensive tissue distribution. Blood-brain barrier (BBB) permeability is low for all compounds, with **4c** at -0.287, **5c** at -0.087, **12** at -0.934, and **11** at -0.987, indicating limited central nervous system penetration, which could be advantageous for avoiding CNS-related side effects. Regarding metabolism, all compounds inhibit CYPIA2 and CYP2C19, but only **12** inhibits CYP2D6, which may lead to drug-drug interactions. The low predicted BBB permeability for all compounds suggests a reduced risk of CNS-related side effects, which is advantageous for non-neurological indications, and the predicted inhibition of CYP enzymes for some compounds highlights a potential for drug-drug interactions that would need to be monitored in future development. For toxicity, all compounds are negative for AMES toxicity, but **12** shows potential skin sensitization (0.8570 probability), and **11** has a higher hERG II inhibition risk (0.8299 probability), indicating possible cardiotoxic concerns. Overall, **4c** appears to have the most favorable pharmacokinetic and physicochemical profile with better solubility, absorption, and lower toxicity risks, while **12** and **11** may face challenges due to poor solubility and higher toxicity risks. Table S9 focuses on oral toxicity predictions for all compounds (**4c**,** 5c**,** 12**, and **11**), all are predicted to be non-mutagenic, non-tumorigenic, non-irritant, and without reproductive effects, as indicated by the negative signs in these categories. This suggests a low likelihood of causing genetic mutations, tumor formation, irritation, or reproductive toxicity, which is a positive attribute for potential drug candidates. Moving to physicochemical properties relevant to oral toxicity, compound **4c** has a drug-likeness score of 0.53, **5c** has 0.25, **12** has 0.15, and **11** has the lowest at 0.10. These scores, combined with their logP, solubility, and TPSA values, are used to predict oral toxicity. The predicted LD50 (lethal dose for 50% of subjects) values for oral administration are as follows: **4c** has an LD50 of 694 mg/kg, **5c** has 679 mg/kg, **12** has 941 mg/kg, and **11** has 623 mg/kg. These values suggest that **12** is the least orally toxic (higher LD50 indicates lower toxicity), while **11** is the most toxic among the four. Overall, while all compounds show no major mutagenic, tumorigenic, irritant, or reproductive concerns, their oral toxicity varies, with **4c** appearing the safest and **11** the most concerning in this aspect. In summary, compound **4c** stands out as the most promising candidate with low tumorigenic, irritant, and reproductive risks, coupled with a high drug score (0.53) due to its balanced a molecular weight of 336.10 g/mol, ClogP (1.56), moderate solubility (-2.74 LogS), and acceptable TPSA (80.47 Å²), indicating good solubility and absorption potential, as well as high gastrointestinal absorption and low toxicity risks. The conclusion of the ADMET section now more clearly ranks the compounds based on their overall profile, explicitly positioning **4c** as the most promising candidate from a pharmacokinetic and toxicity standpoint.

### Pharmacophore modelling

**Fig. 16:**
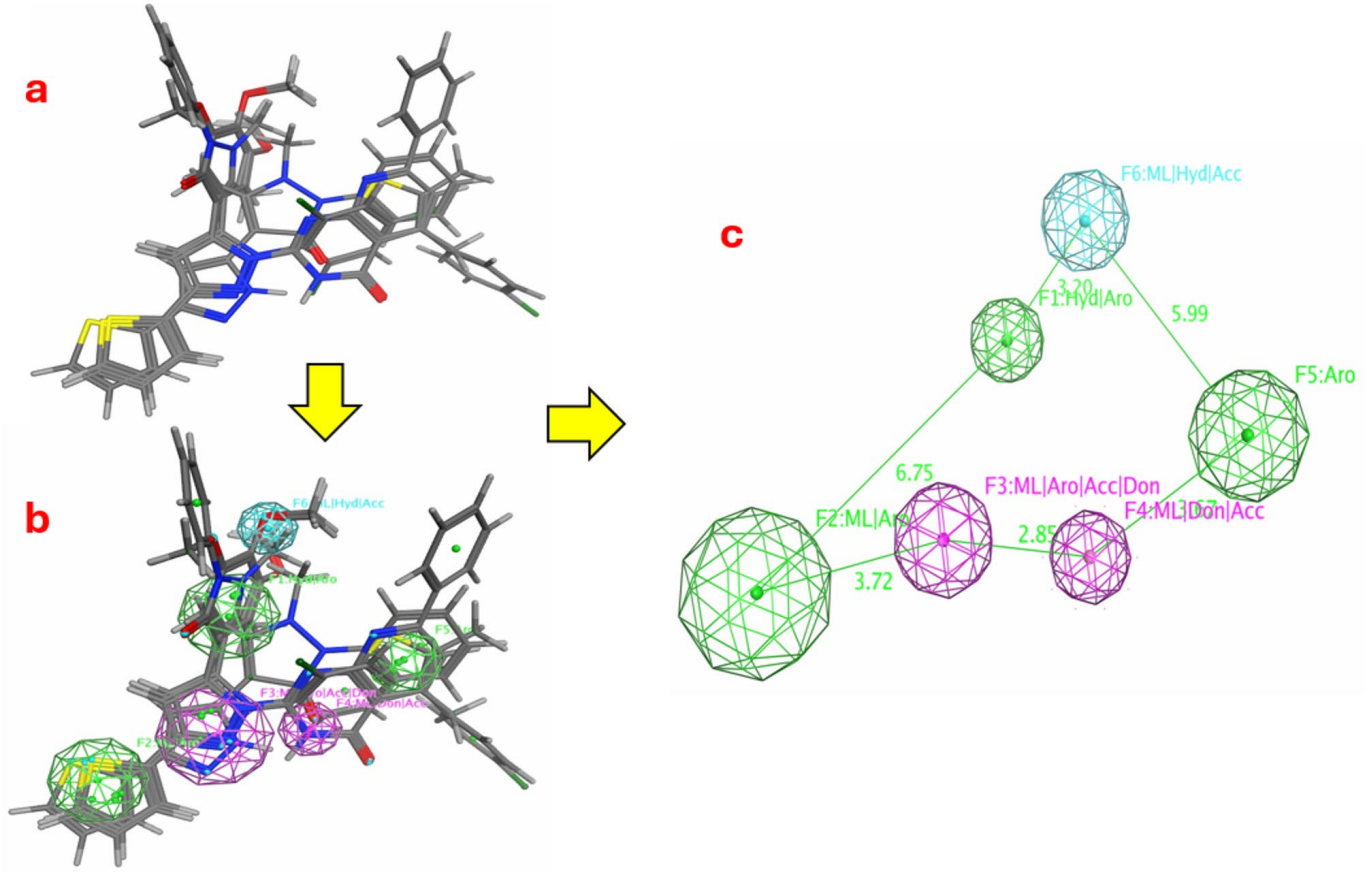
The best Pharmacophore model. (**a**) Illustrates the flexible alignment of compounds set. (**b**) chemical attributes found in Hypo1. (**c**) Displays the three-dimensional configuration and distances calculated between these chemical features.

The data from (Table S10 and Fig. 16) present a comprehensive analysis of pharmacophore models developed to identify critical chemical and spatial features governing a compound’s biological activity. Fourteen hypotheses (F1–F14) were generated, each characterized by specific chemical attributes, scores reflecting model reliability, configurations per molecule, and spatial radii. Top-performing models include F1 (hydrophobic [Hyd] and aromatic [Aro] features) and F2 (metal ligand [ML] and Aro), both achieving 100% scores with 4 configurations per molecule and a compact radius of 1.48 Å, indicative of highly reliable and spatially precise models. F3, which integrates ML, Aro, hydrogen bond acceptor (Acc), and donor (Don) features, also scores 100% but exhibits a larger radius (1.76 Å), suggesting enhanced flexibility to accommodate structural variations while maintaining specificity. Moderately scoring models, such as F4 (ML, Acc, Don) and F5 (Aro), achieve 75% with 3 configurations and smaller radii (0.96–1.15 Å), reflecting narrower spatial tolerance. Lower-scoring hypotheses (F7–F14), scoring 50% with only 2 configurations and radii ranging from 0.53 to 0.71 Å, lack multi-feature complexity, limiting their predictive utility. (Fig. 16) complements these findings by visualizing the compound’s molecular structure with color-coded pharmacophoric features: hydrophobic regions (yellow), aromatic rings (blue), hydrogen bond acceptors (green), and donors (red). Part (b) illustrates the 3D arrangement of these features as interconnected spheres, emphasizing their spatial alignment for optimal target binding. Part (c) aligns with F3, detailing critical distances between pharmacophore elements: 6.75 Å (F3 to F5), 3.72 Å (F3 to F4), and 5.99 Å (F5 to F4), which highlight a geometric precision likely essential for receptor engagement.

The inclusion of multiple interaction types in F3 hydrophobic, aromatic, hydrogen bonding, and metal coordination coupled with its balanced spatial flexibility, positions it as the most robust model (Hypo1) for predicting activity. These results underscore the importance of integrating diverse chemical features and allowing moderate spatial adaptability in pharmacophore design, providing a strategic framework for optimizing drug candidates to enhance binding affinity and selectivity.

### Molecular dynamics simulation (MDS)

**Fig. 17:**
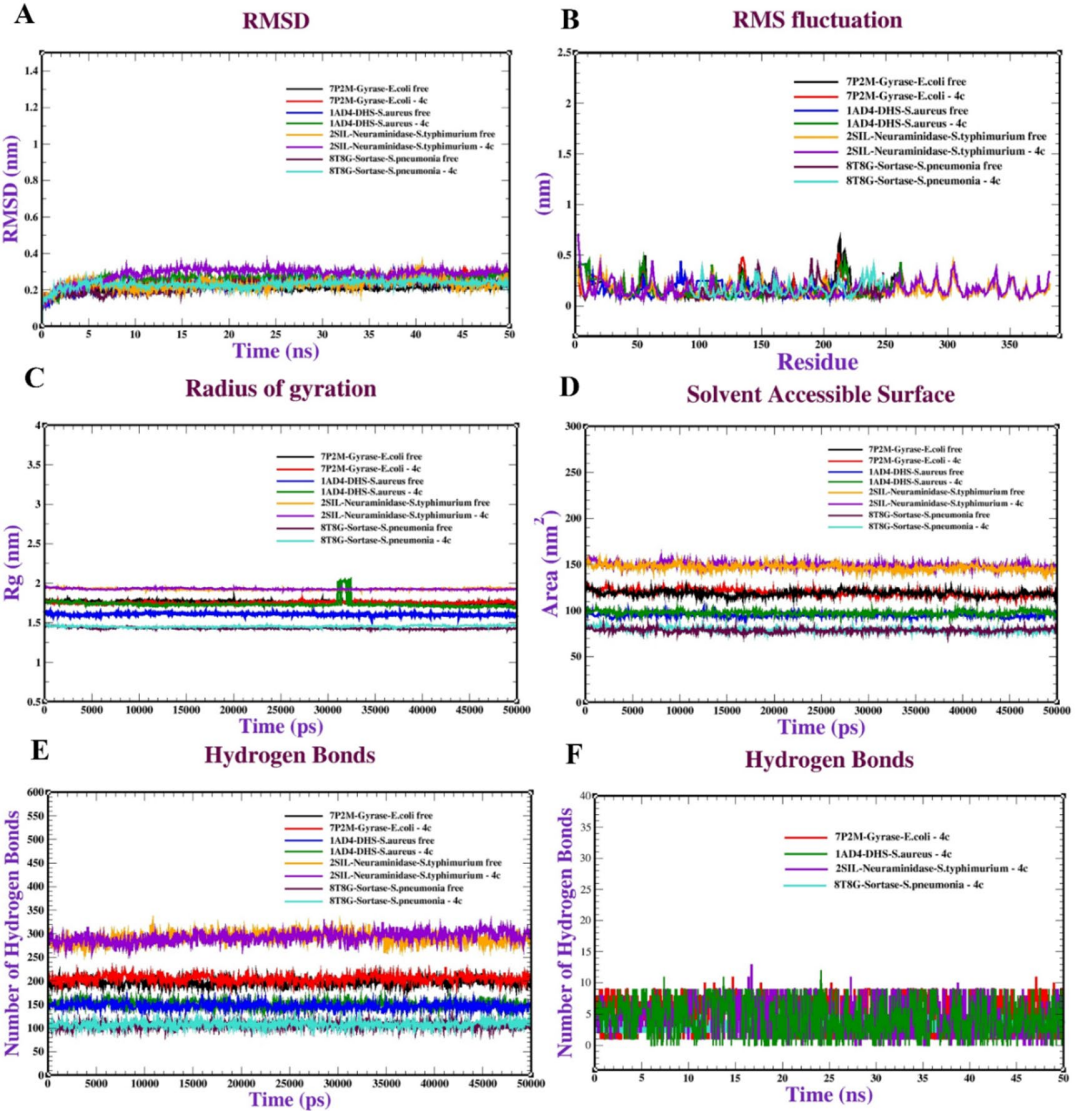
Molecular dynamics of TP2M-Gyrase-*E.coli*, 1AD4-DH5-*S.aureus*, 2SHL-Neuraminidase-*S.typhimurium*, 8TRG-Sortase-*S.pneumonia* proteins complexed with 4c: (**A**) RMSD, (**B**) RMSF, (**C**) Radius of gyration (Rg), (**D**) SASA, (**E**) Intramolecular hydrogen bonds and (**F**) Intermolecular hydrogen bonds.

Molecular dynamics (MD) simulations were conducted on several systems, including TP2M-Gyrase-E.coli, 1AD4-DH5-S.aureus, 2SHL-Neuraminidase-*S*.typhimurium, 8TRG-Sortase-*S*.pneumonia, and their respective free and **4c**-bound forms. The RMSD of these protein-ligand complexes and their unbound states was analyzed over a 50 ns period to assess structural stability. The TP2M-Gyrase-*E*.coli system (both free and **4c**-bound) exhibits an RMSD ranging from 0.2 to 0.4 nm, stabilizing after 10 ns, which suggests strong structural consistency with minimal changes. In contrast, the 1AD4-DHS-*S*.aureus system (free and **4c**-bound) displays a slightly elevated RMSD between 0.3 and 0.5 nm, with the **4c**-bound form showing a noticeable rise after 30 ns, hinting that **4c** binding may cause subtle structural alterations. The 2SHL-Neuraminidase-*S*.typhimurium system (free and **4c**-bound) maintains a steady RMSD of 0.2 to 0.4 nm throughout the simulation, implying that **4c** binding has little impact on its structure. Likewise, the 8TRG-Sortase-*S*.pneumonia system (free and **4c**-bound) shows an RMSD of 0.2 to 0.4 nm with small variations, indicating sustained stability during the simulation. Collectively, all systems exhibit acceptable stability, as their RMSD values remain below 0.5 nm, suggesting that **4c** binding does not significantly disrupt the protein structures over 50 ns (Fig. 17A). The RMSF analysis across the protein systems reveals the flexibility of individual residues during the simulation. For all complexes (free and 4c-bound), RMSF values are generally below 0.5 nm, with occasional spikes reaching 0.6 nm in certain residues, pointing to localized flexibility, likely in loop regions (Fig. 17B). The radius of gyration (Rg) was used to evaluate the compactness of the protein structures. The TP2M-Gyrase-*E*.coli system (free and **4c**-bound) sustains an Rg of about 1.9 to 2.0 nm with slight variations, reflecting a compact and stable conformation over time. The 1AD4-DH5*-S.aureus* system shows a marginally higher Rg of 2.0 to 2.1 nm, with the **4c**-bound state exhibiting a slight increase after 30 ns, indicating a minor structural expansion due to **4c** binding. The 2SHL-Neuraminidase-*S*.typhimurium system maintains an Rg of 1.8 to 1.9 nm, showing consistent compactness throughout. The 8TRG-Sortase-*S.pneumonia* system has a lower Rg of 1.4 to 1.6 nm, also demonstrating stability with minimal changes (Fig. 17C). The solvent accessible surface area (SASA) was analyzed to assess the protein’s exposure to the solvent. The 8TRG-Sortase-**S**.pneumonia, 1AD4-DH5-*S*.aureus, and TP2M-Gyrase-*E*.coli systems (free and **4c**-bound) show SASA values of approximately 70–90 nm², 90–110 nm², and 120–130 nm², respectively, with slight fluctuations, suggesting stable solvent exposure. The 2SHL-Neuraminidase-*S*.typhimurium system, however, has a higher SASA of 140–160 nm², with the **4c**-bound state showing a gradual increase over time, indicating that **4c** binding may enhance the protein’s surface exposure to the solvent (Fig. 17D). The number of hydrogen bonds, which reflects the stability of intra-protein interactions, was also examined. The 8TRG-Sortase-*S*.pneumonia, 1AD4-DH5-*S*.aureus, and TP2M-Gyrase-*E*.coli systems (free and **4c**-bound) maintain approximately 90–100, 145–155, and 190–220 hydrogen bonds, respectively, with minor variations, indicating stable internal interactions. The 2SHL-Neuraminidase-*S*.typhimurium system shows a higher count of 270–300 hydrogen bonds, with the **4c**-bound state slightly increasing over time, suggesting that **4c** binding may strengthen internal hydrogen bonding (Fig. 17E). Intermolecular hydrogen bonds between the protein and **4c** ligand were evaluated over 50 ns to assess the stability of the protein-ligand complex. All systems maintain 0–10 hydrogen bonds with fluctuations, reflecting a stable interaction between the protein and ligand (Fig. 17F). These observations align with the findings of^56^ Abd-Elhalim et al. (2025), who also performed MD simulations to explore the stability and molecular interactions of antimicrobial protein-compound complexes, employing similar metrics such as RMSD, RMSF, SASA, and hydrogen bonds to evaluate their behavior.

### Structure-activity relationships (SAR’s)

Compounds **4a-c**, **5a-c**, **6–12** were evaluated for their antibacterial activity against Gram-positive and Gram-negative bacteria. Substitution patterns involve strategic placement of groups such as (alkyl, halogen or polar moieties) and heterocyclic fusions refer to the integration of additional rings (such as antipyrine, thieno[2,3-*d*]pyrimidine derivative, pyrido[2,3-*d*]pyrimidine derivative or fused imidiazole and triazoles) creating rigid, planar systems. This contrasts with existing linear or monocyclic analogues, gave highly antibacterial efficacy against most types of bacterial strain used providing structural rigidity and multifunctionality. Table 1 showed the results obtained which revealed that Compounds **4c** with (antipyrine attached to the pyrazole backbone), **5c** (with thieno[2,3-*d*]pyrimidine derivative attached to the pyrazole backbone) and **12** (with pyrido[2,3-*d*]pyrimidine derivative attached to the pyrazole backbone) gave highly antibacterial efficacy against most types of bacterial strain used compared to Levofloxacin. Also compound **4c** gave equipotent activity with MIC (8, 6 µmol L^− 1^) as Levofloxacin against Streptococcus pneumonia and Klebsiella pneumonia. Compound **12** gave equipotent activity with MIC (6, 8 µmol L^− 1^) as Levofloxacin against Klebsiella pneumonia and Salmonella typhimurium respectively. Furthermore, compounds **4b**, **9**, and **11** have good activities against all bacteria this was attributed to the presence of (*p*-fluoro) substituent on phenyl attached to the pyrazole moiety **4b**, benzimidiazole attached to the pyrazole derivatives **9** and electron with drawing group (tri-chloro) substituent on phenyl pyrazole derivatives **11**, while compound **5b** has good activity against gram positive and nearly active against gram negative bacteria compared to Levofloxacin this was attributed to the presence of electron withdrawing group (*p*-fluoro) substituent on phenyl and thieno[2,3-*d*]pyrimidine derivatives which attached to the pyrazole. In addition to compounds **4a**, **5a**, **6**, **7**, **8** and **10** exhibited moderate activities toward all bacteria strains.

Compounds **4a-c**, **5a-c**, **6–12** were also evaluated for their anti-inflammatory activity and the results are shown in Table 2. The obtained pharmacological results revealed that most of the test compounds showed significant anti-inflammatory activity with inhibition ranging from 55% to 93%. Compounds 5**c** and **12** have the strongest anti-inflammatory activity compared to the standard drug celecoxib (90%). It was clear that compounds bearing thieno[2,3-*d*]pyrimidine derivatives and antipyrine moieties attached to pyrazole nucleus **5c** displayed excellent activity (93%). Also, for compound **12** the presence of pyrido[2,3-*d*]pyrimidine derivative attached to the pyrazole backbone increases the anti-inflammatory activity (91%). Compound **9** exhibited anti-inflammatory activity (88%) as near as the standard (90%) this may due to the presence of benzimidazole attached to the pyrazole derivatives. Furthermore, compound **5b** exhibited high anti-inflammatory activity (85%) but still less than the standard drug celecoxib (90%) which have electron withdrawing group (*p*-fluoro) substituent on phenyl which has high electronegativity that increases the activity of **5b**, and the presence of thieno[2,3-*d*]pyrimidine moieties which have excellent anti-inflammatory activity according to previous studies^49^. However, compounds **4a-c**, **5a** and **11** exhibited good activity ranging from (62–80%) and, the remaining compounds **7**, **8** and **10** moderate anti-inflammatory activity while, compound **6** showed low activity (55%).

The incorporation of antipyrine moiety, particularly in compound **4c**, significantly boosts antibacterial potency by increasing electron density, facilitating better π-π stacking and hydrogen bonding with bacterial targets like GyraseB and Neuraminidase. Compound **4c** exhibits MIC values of 6–8 µmol/L against Gram-negative bacteria (E. coli, K. pneumoniae, S. typhimurium), outperforming **4a** (trimethoxyphenyl, MIC 12–15 µmol/L) and analogues with chlorine substituent on phenyl. Literature supports that antipyrine moiety enhance antimicrobial efficacy in heterocyclic compounds, as seen in pyrimidine derivatives as in compound **5c** and **12**. Where antipyrine increases activity against Gram-positive strains. For anti-inflammatory activity, compound **5c** contribute to 60–93% edema reduction over 5 h surpassing celecoxib (50–90%).

Halogens, in compound **11** (4-chlorophenyl) derived from halogenated hydrazines, enhance lipophilicity (cLogP 5.96 for **11**) and enable halogen bonding, improving membrane penetration and antibacterial activity (MIC 8–13 µmol/L). The –Cl in **11** provides moderate potency against S. pneumoniae (10 µmol/L), better than non-halogenated analogues, aligning with reports that halogens like Cl increase anti-inflammatory effects in pyrazoles by modulating electron withdrawal and steric fit in enzyme pockets. In anti-inflammatory assays, halogenated derivatives **11** show 50–80% edema reduction, due to improved binding to inflammatory mediators, which is high potent than methoxy variants. The thienopyrimidine core in 5c imparts sulfur-mediated hydrophobic interactions, enhancing antibacterial potency (MIC 5–8 µmol/L) over simple pyrimidines by better inhibiting folate synthesis and DNA replication pathways. Pyridopyrimidine fusion in **12** extends conjugation, yielding the lowest MIC (4–8 µmol/L) and highest anti-inflammatory activity (80–91% reduction), attributed to nitrogen atoms facilitating additional H-bonding. Compared to unfused pyrazoles, these fusions reduce toxicity (low ulcer index 0.44) and improve ADMET profiles, as thienopyrimidines are known for metabolic stability and broad-spectrum activity against resistant strains.

The ulcerogenic activity was screened on the compounds, which exhibited high anti-inflammatory activity **5b**, **5c**, **9**, **10**,** 11** and **12** Table 3. Compounds **12** and **9** exhibited high reduction in ulcerogenic activity was (0.4 ± 0.0) also, compounds **5c** and **5b** exhibited high reduction in ulcerogenic activity was (0.6 ± 0.0), (0.8 ± 0.2) respectively. Finally, compounds **10** and **11** showed moderate severity indexes.

Traditional pyrimidine derivatives often exhibit moderate antibacterial activity (MIC > 10 µmol/L against E. coli and S. aureus), but our fused thienopyrimidine and pyridopyrimidine systems enhance rigidity and lipophilicity, leading to stronger enzyme inhibition (DNA gyrase, DHPS) and lower MIC values (4–8 µmol/L). This is supported by thienopyrimidines and pyridopyrimidine derivatives that provide broader antimicrobial spectra and better bioavailability than unfused pyrimidines due to the sulfur atom contribution to hydrophobic interactions and metabolic stability. Similarly, *N*-pyrazolyl modifications improve selectivity and reduce ulcerogenic liability compared to celecoxib analogues, with compounds **5c** and **12** showing up to 65% edema reduction (vs. 50% for celecoxib) and ulcer indices of 0.34–0.48 (vs. 0.9 for celecoxib). These enhancements address limitations in existing analogues, such as poor solubility or narrow activity spectra, positioning our compounds as promising leads for multidrug-resistant infections and inflammation.

The Table 4 includes compound key substituent/structural feature (e.g., trimethoxyphenyl, Cl, thienopyrimidine fusion), average MIC (µmol/L) against Gram-positive and Gram-negative bacteria, %edema reduction at 5 h, and ulcer index. This table correlates how electron-withdrawing groups and heterocyclic fusions lower MIC and improve anti-inflammatory efficacy, while electron donating groups (–OCH_3_) provide moderate enhancements. These additions provide a more comprehensive SAR analysis, strengthening scientific depth and translational relevance.

**Table 4:** Summary correlating substituent type with observed biological activity.

Compound	Substituent/feature	Avg. MIC G(+ ve) (µmol/L)	Avg. MIC G(-ve) (µmol/L)	% Edema reduction (5 h)	Ulcer INDEX
**4a**	3,4,5-Trimethoxyphenyl (electron-donating)	13.3	13.7	41.5	–
**4c**	antipyrine	8.0	6.7	65.7	–
**5c**	Thienopyrimidine	7.0	6.3	93.0	0.34
**11**	2,4,6 trichloro-phenyl(halogen)	9.0	11.3	80.0	0.53
**12**	Pyridopyrimidine	4.7	7.0	91.0	0.44
**Levofloxacin** **Celecoxib**	–	6.7	8.0	90.0	0.90

## Experimental

### Chemistry

All melting points were measured on an Electrothermal 9100 series digital melting point apparatus (Shimadzu, Japan). Microanalytical data were gathered with a Vario Elementar apparatus (Shimadzu). Elemental analyses of all compounds were within ± 0.4% of the theoretical values. The IR spectra (KBr) were recorded on a Perkin Elmer 1650 spectrometer (USA). ^1^H NMR and ^13^C NMR spectra were recorded on a JEOL ECA-500 (Shimadzu) instruments. Chemical shifts were expressed in ppm relative to SiMe_4_ as internal standard in DMSO-*d*_*6*_ as a solvent. Mass spectra were recorded on a 70 eV Finnigan SSQ 7000 spectrometer (Thermo-Instrument System Incorporation, USA). The purity of the compounds was checked on aluminum plates coated with silica gel (Merck, Germany). Chemicals and solvents (Analar ≥ 99%) were purchased from Sigma-Aldrich (Saint Lewis, USA). Levofloxacin was supplied by Hoechst-Roussel Pharmaceuticals Inc., Somerville, New Jersey, USA.

### General procedure for syntheses

Starting material chalcones **3a-c** was prepared according to literature procedures^57^, stirring of 2-acetyl-thiophene **1** with various aldehydes **2a-c** namely (3,4,5-trimethoxy-benzaldhyde, 4-fluorobenzaldhyde and 4-antipyrine carboxaldehyde) in ethanol and sodium hydroxide solution afforded the chalcones **3a-c** The solid formed was collected by filtration, washed with water (30 mL), dried and recrystallized from ethanol.

#### Synthesis of 1-(thiophen-2-yl)-3-(3,4,5-trimethoxyphenyl)prop-2-en-1-one **3a**

Pale yellow powder, Yield: 80%, mp. 151–152 °C; IR (KBr, cm^− 1^); 3091 (CH aryl), 2927, 2868 (CH aliphatic) 1701 (C = O), 1635 (C = C); ^1^H NMR (500 MHz, CDCl_3_, *δ*, ppm); 3.89, 3.91 (2s, 9 H, 3OCH_3_), 6.19–6.29 (d, 1H, =CH, *J =* 9.20 Hz), 6.85 (s, 2 H, Ar-*H*), 7.16–7.18 (t, 1H, thiophene ), 7.30 (d, 1H, CH-thiophene, *J =* 5.02 Hz), 7.67 (d, 1H, =CH, *J =* 9.20 Hz), 7.86–7.87 (d, 1H, CH-thiophene, *J =* 4.90 Hz),^13^C-NMR (CDCl_3_ *δ*, ppm): 56.46, 60.27, 60.46, 103.11, 103.55, 125.36, 127.11, 128.57, 131.47, 131.54, 136.36, 140.25, 150.70, 153.64, 153.90, 182.12. Elemental analysis for C_16_H_16_O_4_S (304.36); Anal. Calcd.; C, 63.14; H, 5.30; S, 10.53%, found C, 63.10; H, 5.28; S, 10.48%.

#### Synthesis of (3-(4-fluorophenyl)-1-(thiophen-2-yl) prop-2-en-1-one **3b**

Pale brown powder, Yield: 73%, mp. 120–122 °C; IR (KBr, cm^− 1^); 3061(CH aryl), 2960(CH aliphatic) 1695 (C = O), 1635 (C = C); ^1^H NMR (500 MHz, CDCl_3_, *δ*, ppm); 6.13–6.17 (d, 1H, *J =* 7.40 Hz, =CH), 7.10–7.11(d, 2 H, *J =* 8.2 Hz, Ar-*H*), 7.12–7.19 (m, 2 H, CH-thiophene + = CH), 7.90–7.91 (d, 2 H, *J =* 8.2 Hz, Ar-*H*), 8.18–8.19 (d, 1H, *J =* 5.20 Hz, CH-thiophene), 8.36–8.39 (d, 1H, *J =* 4.90 Hz, CH-thiophene),^13^C-NMR (CDCl_3_ *δ*, ppm): 115.45, 128.92, 129.75, 133.55, 134.84, 139.17, 152.37, 163.18, 182.12. Elemental analysis for C_13_H_9_FOS (232.27); Anal. Calcd.; C, 67.22; H, 3.91 S, 13.80%, found C, 67.20; H, 3.88; S, 13.77%.

#### Synthesis of 1,5-dimethyl-4-(3-oxo-3-(thiophen-2-yl) prop-1-en-1-yl)-2-phenyl-1,2-dihydro-3H-pyrazol-3-one **3c**

Deep yellow powder, Yield: 60%, mp. 210–213 °C; IR (KBr, cm^− 1^); 3061(CH aryl), 2918(CH aliphatic) 1695, 1701 (2 C = O), 1643 (C = C); ^1^H NMR (500 MHz, DMSO, *δ*, ppm); 2.64 (s, 3 H, CH_3_), 2.99 (s, 3 H, NCH_3_), 7.20–7.25 (t, 1H, *J =* 5.10 Hz, CH-thiophene),7.33–7.34 (d, 2 H, Ar-*H +* CH = CH), 7.40–7.42 (m, 1H, CH = CH), 7.50–7.55 (m, 3 H, Ar-*H*), 7.77–7.78 (d, 1H, *J =* 5.10 Hz, CH-thiophene), 7.90–7.93 (m, 2 H, CH-thiophene + Ar-*H*), ^13^C-NMR (DMSO *δ*, ppm): 10.95, 35.00, 102.09, 116.66, 126.59, 129.00, 129.30, 129.80, 132.80, 134.41, 134.51, 146.37, 154.34, 163.18 (O = C-N), 182.12 (C = O). Elemental analysis for C_18_H_16_N_2_O_2_S (324.40); Anal. Calcd.; C, 66.65; H, 4.97; N, 8.64, S, 9.88%, found C, 66.61; H, 4.96; N, 8.60; S, 9.84%.

### General procedure for syntheses of **4a-c**

A mixture of compounds **3a-c** (5 mmol) and hydrazine hydrate (2 mL) in absolute ethanol was heated under reflux for 10 h. The progress of the reaction was followed by TLC. After cooling to room temperature, the solid formed was filtered off, washed with ethanol, dried, and crystallized from a proper solvent.

#### 3-(thiophen-2-yl)-5-(3,4,5-trimethoxyphenyl)-1H-pyrazole **4a**

Pale brown powder, crystallized from dioxane, Yield: 70%, mp. 183–184 C°; IR (KBr, cm^− 1^); 3382 (NH), 3055 (CH aryl), 2912 (CH aliphatic), 1573 (C = N); ^1^H NMR (500 MHz, DMSO-*d*_6_, δ, ppm); 3.76, 3.79, 3.82 (3s, 9 H, 3OCH_3_), 5.46 (s, 1H, CH-pyrazole) 6.69 (s, 2 H, Ar-*H*), 7.11 (d, 1H, *J =* 5.00 Hz, CH- thiophene), 7.40–7.49 (t, 1H, *J =* 5.10 Hz, CH-thiophene), 7.69–7.70 (d, 1H, *J =* 5.20 Hz, CH-thiophene), 11.21 (s, 1H, NH), (NH, D_2_O exchangeable); ^13^C-NMR (DMSO-d_6_) δ ppm: 56.46, 60.27, 60.47, 103.43, 123.07,127.62, 127.99, 128.58, 129.11, 130.32, 135.42, 136.63, 140.38, 146.20, 151.75, 157.73. Elemental analysis for C_16_H_16_N_2_O_3_S (316.38); Anal. Calcd.; C, 60.74; H, 5.10; N, 8.85; S, 10.13%, found C, 60.70; H, 4.96; N, 8.83; S, 10.11%.

#### 5-(4-fluorophenyl)-3-(thiophen-2-yl)-1H-pyrazole **4b**

White crystal, crystallized from cyclohexane, Yield: 64%, mp. 192–193 C°; IR (KBr, cm^− 1^); 3380(NH), 3060 (CH aryl), 2900 (CH aliphatic), 1639 (C = N); ^1^H NMR (500 MHz, DMSO-*d*_6_, δ, ppm); 6.06 (s, 1H, CH-pyrazole), 6.69–6.78 (t, 1H *J =* 4.90 Hz, CH- thiophene), 7.10–7.14 (dd, 2 H, *J* = 7.10 Hz, Ar-H), 7.45–7.50 (d, 1H *J =* 5.00 Hz, CH-thiophene), 7.90–7.93(dd, 2 H, *J* = 7.10 Hz, Ar-H), 8.38–8.45 (d, 1H *J =* 5.20 Hz, CH thiophene), 11.88 (s, 1H, NH), (NH, D_2_O exchangeable); (NH, D_2_O exchangeable); ^13^C-NMR (DMSO-d_6_) δ ppm: 99.55, 106.69, 112.18, 116.14, 116.32, 127.75, 127.82, 137.45, 143.06, 161.40, 163.35. Elemental analysis for C_13_H_9_FN_2_S (244.29); Anal. Calcd.; C, 63.92; H, 3.71; N, 11.47; S, 13.12%, found C, 63.90; H, 3.68; N, 11.44; S, 13.09%.

#### 1’,5’-dimethyl-2’-phenyl-5-(thiophen-2-yl)-1’,2’-dihydro-2 H,3’H-[3,4’-bipyrazol]-3’-one **4c**

Pale yellow powder, crystallized from DMF, Yield: 59%, mp. 215–217 C°; IR (KBr, cm^− 1^); 3381(NH), 3057 (CH aryl), 2921, 2851 (CH aliphatic), 1706 (C = O), 1596 (C = N); ^1^H NMR (500 MHz, DMSO-*d*_6_, δ, ppm); 2.22 (s, 3 H, CH_3_), 3.55 (s, 3 H, NCH_3_), 6.13 (s, 1H, CH-pyrazole), 6.40–6.71 (m, 2 H, Ar-H ), 7.19–7.21 (d, 1H *J =* 4.90 Hz, CH- thiophene), 7.22–7.36 (m, 1H, CH- thiophene), 7.62–7.63 (d, 1H *J =* 5.10 Hz, CH thiophene) 7.90–7.92 (d, 2 H *J* = 8.00 Hz, Ar-H), 8.19 (s, 1H, Ar-H), 12.73 (s, 1H, NH), (NH, D_2_O exchangeable); ^13^C-NMR (DMSO-d_6_) δ ppm: 15.35, 39.30, 92.32, 100.00, 102.95, 115.84, 128.36, 130.03, 133.57, 136.77, 134.02, 136.36, 143.95, 158.67, 159.08, 159.44, 161.71, 165.42. Elemental analysis for C_18_H_16_N_4_OS (336.41); Anal. Calcd.; C, 64.27; H, 4.79; N, 16.65; O, 4.76; S, 9.53%, found 64.24; H, 4.75; N, 16.62; O, 4.74; S, 9.50%.

### General procedure for syntheses of **5a-c**

A mixture of compounds **3a-c** (5 mmol) and 2-hydrazineyl-5,6-dimethylthieno[2,3-*d*] pyrimidin-4(3*H*)-one (1.10 g, 5mmol) was heated under reflux for 10 h in ethanol/piperdine. The precipitate was filtered off and washed several times with cold EtOH. The solid was recrystallized from DMF.

#### 5,6-dimethyl-2-(3-(thiophen-2-yl)-5-(3,4,5-trimethoxyphenyl)-1 H-pyrazol-1-yl)thieno [2,3-d]pyrimidin-4(3H)-one **5a**

Orange powder, Yield: 77%, mp. 241–242 C°; IR (KBr, cm^− 1^); 3361 (NH), 3055 (CH aryl), 2960, 2870 (CH aliphatic), 1695 (C = O); 1635 (C = N); ^1^H NMR (500 MHz, CDCl_3_, δ, ppm); 2.22, 2.34 (2s, 6 H, 2CH_3_) 3.77, 3.85, 3.89 (3s, 9 H, 3OCH_3_), 5.72 (s, 1H, CH-pyrazole), 6.49 (s, 2 H, Ar-H), 7.04–7.10 (m, 1H, CH-thiophene), 7.22–7.25 (d, 1H *J =* 5.00 Hz, CH-thiophene), 7.43–7.44 (d, 1H *J =* 5.00 Hz,, CH-thiophene) 9.86 (s, 1H, NH), (NH, D_2_O exchangeable);^13^C-NMR (CDCl_3_) δ ppm: 12.65, 13.05, 56.36, 60.81, 61.73, 103.15, 103.29, 112.18, 117.14, 125.36, 127.92, 128.86, 129.42, 129.64, 134.02, 136.36, 146.29, 150.12, 153.63, 158.19, 163.20. Elemental analysis for C_24_H_22_N_4_O_4_S_2_ (494.58); Anal. Calcd.; C, 58.28; H, 4.48; N, 11.33; S, 12.96%, found C, 58.24; H, 4.45; N, 11.30; S, 12.92%.

#### 2-(5-(4-fluorophenyl)-3-(thiophen-2-yl)-1 H-pyrazol-1-yl)-5,6-dimethylthieno[2,3-d] pyrimidin-4(3 H)-one **5b**

Gray powder, Yield: 68%, mp. 212–213 C°; IR (KBr, cm^− 1^); IR (KBr, cm^− 1^); 3327 (NH), 3055 (CH aryl), 2958, 2870 (CH aliphatic), 1672 (C = O); 1643 (C = N); 2.13, 2.35 (2s, 6 H, 2CH_3_), 6.34 (s, 1 H, CH-pyrazole), 7.19–7.38 (dd, 2 H, *J* = 7.80 Hz, Ar-H), 7.69–7.78 (m, 3 H, CH-thiophene+ 2 H, Ar-H) 7.85–7.87 (d, 1 H, *J* = 5.10 Hz CH-thiophene), 8.02–8.10 (d, 1 H *J* = 5.20 Hz, CH-thiophene), 11.21 (s, 1 H, NH), (NH, D_2_O exchangeable); ^13^C-NMR (DMSO-*d*_6_) δ ppm: 10.95, 12.67, 90.54, 100.89, 103.29, 115.65, 121.74, 123.43, 125.88, 127.00, 130.16, 133.84, 135.74, 136.97, 138.83, 154.77, 157.98, 158.09, 161.35. Elemental analysis for C_21_H_15_FN_4_OS_2_ (422.50); Anal. Calcd.; C, 59.70; H, 3.58; N, 13.26; S, 15.18%, found C, 59.68; H, 3.55; N, 13.24; S, 15.16%.

#### 2-(1’,5’-dimethyl-3’-oxo-2’-phenyl-5-(thiophen-2-yl)-2’,3’-dihydro-1’H,2H-[3,4’-bipyrazol]-2-yl)-5,6-dimethylthieno[2,3-d]pyrimidin-4(3 H)-one **5c**

Pale yellow powder, Yield: 68%, mp. 260–262 C°; IR (KBr, cm^− 1^); IR (KBr, cm^− 1^); 3219 (NH), 3051 (CH aryl), 2958, 2870 (CH aliphatic), 1701, 1672 (2 C = O); 1579 (C = N); 2.18 (s, 3 H, CH_3_), 2.46–2.62 (m, 6 H, 2CH_3_), 3.16 (s, 3 H, NCH_3_) 5.72 (s, 1 H, pyrazole), 7.32–7.37 (m, 2 H, CH-thiophene + Ar-H ), 7.43–7.47 (m, 3 H, 2CH-thiophene + Ar-H ), 8.16–8.17 (m, 3 H, Ar-H) 11.86 (s, 1 H, NH), (NH, D_2_O exchangeable); ^13^C-NMR (DMSO-*d*_6_) δ ppm: 12.68, 13.85, 15.35, 32.04, 101.61, 106.69, 110.42, 122.90, 125.94, 126.04, 126.39, 126.65, 126.96, 128.92, 129.75, 133.55, 134.84, 139.13, 150.81, 152.06, 153.41, 158.16, 160.10, 164.00. Elemental analysis for C_26_H_22_N_6_O_2_S_2_ (514.62); Anal. Calcd.; C, 60.68; H, 4.31; N, 16.33; S, 12.46%, found C, 60.65; H, 4.29; N, 16.30; S, 12.42%.

#### Thiophen-2-yl(3-(3,4,5-trimethoxyphenyl) oxiran-2-yl) methanone **6**

To a solution of compound **3a** (1.5 g, 5 mmol) in mixture of dry acetone (30 mL), methanol (15mL), H_2_O_2_ (20%, 10mL) and anhydrous NaOH (1 g) were added portion-wise. The resulting mixture was stirred in an ice bath until the yellow color is disappeared; then, the mixture was allowed to stir for 1 h. at 40 °C until clear solution was achieved, then stirring was continued at room temperature for 3 h. crushed ice was added and the solid product was filtered and re-crystallized from ethanol.

White powder, Yield: 65%, mp. 241–242 C°; IR (KBr, cm^− 1^); 3061 (CH aryl), 2960, 2868(CH aliphatic), 1695 (C = O),^1^H NMR (500 MHz, DMSO-*d*_6_, δ, ppm); 3.89–3.91 (m, 11 H, 3OCH_3_+ 2OCH), 7.25 (s, 2 H, Ar-H), 7.67–7.70 (t, 1H *J* = 5.10 Hz, CH-thiophene), 7.74–7.77 (d, 1H *J* = 5.40 Hz, CH-thiophene), 7.86–7.94 (d, 1H *J* = 4.90 Hz, CH-thiophene), ^13^C-NMR (CDCl_3_) δ ppm: 56.22, 59.66, 60.91, 61.91, 102.60, 128.58, 130.95, 133.72, 135.38, 138.60, 141.04,153.72, 186.31. Elemental analysis for C_16_H_16_O_5_S (320.36); Anal. Calcd.; C, 59.99; H, 5.03; S, 10.01%, found C, 59.96; H, 5.00; S, 10.01%.

#### 1-(3-(thiophen-2-yl)-5-(3,4,5-trimethoxyphenyl)-1 H-pyrazol-1-yl)ethan-1-one **7**

A mixture of compounds **3a** (1.5 g, 5 mmol) and hydrazine hydrate (100%, 3mL)/acetic acid (25mL) mixture was allowed to heat under reflux for 12 h. The precipitate was filtered off and washed several times with water. The solid product was recrystallized from dioxane. White powder, Yield: 80%, mp. 139–140 C°; IR (KBr, cm^− 1^); 3089 (CH aryl), 2954, 2881 (CH aliphatic), 1712 (C = O), 1635 (C = N); ^1^H NMR (500 MHz, DMSO-*d*_6_, δ, ppm); 2.24 (s, 3 H, CH_3_), 3.76, 3.79, 3.82 (3s, 9 H, 3OCH_3_), 5.96 (s, 1H, pyrazole), 6.42 (s, 2 H, Ar-H), 7.11–7.12 (m, 1H, CH-thiophene), 7.40–7.42 (d, 1H *J =* 4.90 Hz, CH-thiophene), 7.69–7.70 (d, 1H *J =* 5.10 Hz, CH-thiophene), ^13^C-NMR (DMSO-*d*_6_) δ ppm: 22.22, 56.46, 60.27, 60.47, 100.02, 128.19, 128.57, 129.99, 130.72, 137.21, 138.51, 145.87, 150.70, 153.64, 153.90, 170.13. Elemental analysis for C_18_H_18_N_2_O_4_S (358.41); Anal. Calcd.; C, 60.32; H, 5.06; N, 7.82; S, 8.95%, found C, 60.29; H, 5.04; N, 7.79; S, 8.93%.

#### 1-(3-(thiophen-2-yl)-5-(3,4,5-trimethoxyphenyl)-1 H-pyrazol-1-yl) ethan-1-one oxime **8**

Compound **7** (1.79 g, 5 mmol) was added into a solution of potassium hydroxide (10 mmol), hydroxylamine hydrochloride (15 mmol) in ethanol (30 mL) at the room temperature. Then the reaction mixture was heated under reflux for 15 h and poured into ice water (50 mL), the precipitate was filtered off washed by water then dried, and crystallized from DMF. Gray powder, Yield: 60%, mp. 253–255 C°; IR (KBr, cm^− 1^); 3434 (OH), 3092 (CH aryl), 2969, 2921 (CH aliphatic), 1640 (C = N-OH), ^1^H NMR (500 MHz, DMSO-*d*_6_, δ, ppm); 2.18 (s, 3 H, CH_3_), 3.88, 3.90 (2s, 9 H, 3OCH_3_) 6.76 (s, 1H, 1H pyrazole), 7.08–7.12 (m, 2 H, Ar-H), 7.25–7.47 (t, 1H *J =* 4.90 Hz, CH-thiophene), 7.56–7.70 (d, 1H *J =* 5.00 Hz, CH-thiophene), 7.78–7.85 (s, 1H *J =* 5.00 Hz, CH-thiophene), 11.02 (s, 1H, OH) (OH, D_2_O exchangeable); ^13^C-NMR (DMSO-*d*_6_) δ ppm: 27.27, 56.46, 60.27, 60.47, 102.20, 121.74, 123.43, 125.88, 127.00, 128.57, 133.35, 135.08, 135.53, 136.97, 138.83, 152.89, 155.36. Elemental analysis for C_18_H_19_N_3_O_4_S (373.43); Anal. Calcd.; C, 57.90; H, 5.13; N, 11.25; S, 8.59%, found C, 57.87; H, 5.10; N, 11.21; S, 8.55%.

#### N-(1 H-benzo[d]imidazol-2-yl)-1-(3-(thiophen-2-yl)-5-(3,4,5-trimethoxyphenyl)-1H-pyrazol-1-yl)ethan-1-imine **9**

Compound **7** (1.79 g, 5 mmol) and 2-aminobenzimidiazole (0.65 g, 5 mmol) was heated under reflux in ethanol (25mL)/acetic acid (3mL) for 8 h. the reaction mixture was poured onto ice and the solid product was filtered off and washed several times with water. The solid product was recrystallized from dioxane.

Brown powder, Yield: 64%, mp. 292–294 C°; IR (KBr, cm^− 1^); 3361 (NH), 3097 (CH aryl), 2932, 2849(CH aliphatic), 1592(C = N), ^1^H NMR (500 MHz, DMSO-*d*_6_, δ, ppm); 1.25 (s, 3 H, CH_3_), 3.79, 3.92, 3.93 (3s, 9 H, 3OCH_3_) 6.58 (s, 1H, pyrazole), 6.85 (s, 2 H, Ar-H) 7.19–7.38 (m, 2 H, CH-thiophene + Ar-H), 7.69–7.78 (m, 2 H, Ar-H), 7.78–7.85 (d, 1H *J =* 5.00 Hz, CH-thiophene), 7.87–7.90 (d, 1H, Ar-H), 7.90–8.02 (d, 1H *J =* 5.00 Hz, CH-thiophene), 12.57 (s, 1H, NH) (NH, D_2_O exchangeable); ^13^C-NMR (DMSO-*d*_6_) δ ppm: 13.04, 56.36, 60.82, 61.73, 101.61, 103.38, 107.20, 110.42, 115.45, 124.13, 126.82, 128.92, 129.75, 133.55, 134.84, 139.17, 144.78, 152.37, 153.58, 158.19. Elemental analysis for C_25_H_23_N_5_O_3_S (473.55); Anal. Calcd.; C, 63.41; H, 4.90; N, 14.79; S, 6.77%, found C, 63.38; H, 4.88; N, 14.75; S, 6.73%.

#### 3-(thiophen-2-yl)-5-(3,4,5-trimethoxyphenyl)-1 H-pyrazole-1-carbothioamide **10**

To a solution of **3a** (1.5 g, 5 mmol) in ethanolic sodium hydroxide (50 mL), thiosemicarbazide (10 mmol) was added and the reaction mixture was heated under reflux for 4 h. After cooling, the reaction mixture was poured onto ice/HCl mixture and the solid that separated was washed with cold water and filtered off and crystallized from dioxane.

Brown powder, Yield: 60%, mp. 180–182 C°; IR (KBr, cm^− 1^); 3417, 3327 (NH_2_), 3055 (CH aryl), 2958, 2870 (CH aliphatic),1575 (C = N); ^1^H NMR (500 MHz, DMSO-*d*_6_, δ, ppm); 3.83, 3.85, 3.89 (s, 9 H, 3OCH_3_), 6.07–6.15 (brs, 2 H, NH_2_ D_2_O exchangeable), 6.34 (s, 1H, CH pyrazole), 6.64 (s, 2 H, CH-Ar-H), 7.27–7.28 (d, 1H *J =* 5.10 Hz, CH-thiophene), 7.53–7.54 (t, 1H *J =* 4.90 Hz, CH-thiophene), 7.74–7.76 (d, 1H *J =* 5.10 Hz, CH-thiophene); ^13^C-NMR (DMSO-*d*_6_) δ ppm: 56.36, 60.81, 61.73, 101.61, 103.15, 103.29, 125.36, 127.92, 128.86, 129.42, 129.64, 134.02, 136.36, 150.12, 153.63, 176.03. Elemental analysis for C_17_H_17_N_3_O_3_S_2_ (375.46); Anal. Calcd.; C, 54.38; H, 4.56; N, 11.19; S, 17.08%, found C, 54.35; H, 4.54; N, 11.15; S, 17.08%.

#### 3-(thiophen-2-yl)-1-(2,4,6-trichlorophenyl)-5-(3,4,5-trimethoxyphenyl)-1H-pyrazole **11**

A mixture of compounds **3a** (1.5 g, 5 mmol) and 2,4,6-trichlorophenyl hydrazine (5 mmol) in ethanol/K_2_CO_3_ was allowed to heat under reflux for 10 h. The solution was filtered off and poured onto ice water. The solid product was collected dried and recrystallized from dioxane.

Deep green powder, Yield: 70%, mp. 232–234 C°; IR (KBr, cm^− 1^); 3097 (CH aryl), 2969, 2849 (CH aliphatic), 1620 (C = N); 3.77, 3.90, 3.93 (s, 9 H, 3OCH_3_), 6.57(s, 1 H, pyrazole), 7.19–7.25 (t, 1 H *J =* 4.95 Hz, CH-thiophene), 7.38–7.69 (m, 2 H, Ar-H), 7.78–7.85 (m, 3 H, Ar-H + CH-thiophene), 7.78–7.79 (d, 1 H *J =* 5.10 Hz, CH-thiophene), ^13^C-NMR (DMSO-*d*_6_) δ ppm: 56.46, 60.27, 60.47, 100.02, 102.88, 103.11, 103.55, 115.17, 117.14, 125.36, 127.92, 128.57, 129.99, 130.72, 130.84, 137.21, 138.51, 150.70, 153.64, 153.90. Elemental analysis for C_22_H_17_Cl_3_N_2_O_3_S (495.80); Anal. Calcd.; C, 53.30; H, 3.46; N, 5.65; S, 6.47%, found C, 53.27; H, 3.42; N, 5.61; S, 6.45%.

#### 5-(4-chlorophenyl)-7-phenyl-2-(3-(thiophen-2-yl)-5-(3,4,5-trimethoxyphenyl)-1H-pyrazol-1-yl) pyrido[2,3-d] pyrimidin-4(3 H)-one **12**

A mixture of compounds **3a** (1.5 g, 5 mmol) and 5-(4-chlorophenyl)-2-hydrazineyl-7-phenyl-pyrido[2,3-*d*]pyrimidin-4(3*H*)-one (1.82 g, 5 mmol) in ethanol/piperidine was allowed to heat under reflux for 12 h. The reaction mixture was allowed to cool at room temperature then poured onto ice/HCl mixture. The precipitate was filtered off and washed several times with ice water. The solid product was recrystallized from DMF. Deep yellow powder, Yield: 79%, mp. 257–259 C°; IR (KBr, cm^− 1^); 3361 (NH), 3061 (CH Ar.), 2960, 2868 (CH alph.), 1695 (C = O), 1635 (C = N); ^1^H NMR (500 MHz, DMSO-*d*_6_, δ, ppm); 3.89, 3.91 (s, 9 H, 3OCH_3_), 6.64 (s, 1H, pyrazole), 7.27–7.36 (m, 9 H [2 H CH-thiophene +7 H Ar-H]), 7.41–7.43 (d, 1H *J =* 4.95 Hz, thiophene), 7.91–7.93 (d, 2 H *J =* 7.50 Hz, Ar-H) 8.41–8.50 (d, 2 H *J =* 7.50 Hz, Ar-H), 8.95 (s, 1H, pyrimidine), 11.88 (s, 1H, NH) (NH, D_2_O exchangeable); ^13^C-NMR (DMSO-*d*_6_) δ ppm: 56.36, 60.81, 61.73, 100.02, 102.88, 103.11, 103.55, 109.40, 116.14, 122.90, 126.85, 128.57, 128. 60, 128.71, 129.30, 129.50, 129.98, 130.30, 130.45, 130.72, 130.84, 137.20, 138.51, 147.80, 150.70, 153.64, 153.90, 155.11, 160.96, 163.20, 164.00. Elemental analysis for C_35_H_26_ClN_5_O_4_S (648.13); Anal. Calcd.; C, 64.86; H, 4.04; N, 10.81; S, 4.95%, found C, 64.86; H, 4.04; N, 10.81; S, 4.92%.

### Biology

#### Antibacterial activity

The newly synthesized compounds was tested in vitro against the Gram-positive bacteria: *Streptococcus pneumonia* ATCC 17,691, *Staphylococcus aureus* ATCC 16,832, *Bacillus subtilis* ATCC18382 and Gram-negative bacteria: *Escherichia coli* ATCC 28,935, *Klebsiella pneumonia* ATCC 26,845 and *Salmonella typhimurium* ATCC 27,685 and all microorganisms were purchased from the American Type Culture Collection (Manassas, USA). The newly synthesized compounds were dissolved in DMSO and tested for antibacterial activity by the agar disk diffusion technique^58^, using a microplate-wells, 1 cm in diameter and a solution of 100 µg mL^-1^ of the test compound. Compound impregnated disks were placed on an agar plate containing a standard suspension of microorganisms. The plate was incubated for 24 h at 37 °C. Diameters of inhibition zones were measured with calipers or automated scanners and were compared with those of the standards. Levofloxacin (0.15 µmol L^-1^) was used as a reference drug. Serial plate dilution technique^59,60^ was used for assessment of (*MIC*) minimum inhibitory concentration. 5 mg of each test compound was dissolved in 1 mL of dimethylsulfoxide to prepare the stock solution. Serial dilutions were prepared from the stock solution. The plates were incubated at 37 °C for 24 h. *MIC* was the lowest concentration (µmol L^-1^) of the test compound that resulted in no visible growth on the plates. DMSO was used as a solvent control to ensure that the solvent had no effect on bacterial growth. The results of antibacterial activities are summarized in Table 1.

### Experimental animal protocol

This study utilized adult female albino rats 150–180 g. The animal house colony of the National Research Center, Cairo, Egypt, provided the rats. Animals were housed under standard environmental conditions (22 ± 3 °C, 55 ± 5% humidity, and 12 h light/dark cycles) and received standard pellet diet and water libitum. Prior to the experiment, both control (water) and experimental rats (given synthesized compounds) were fasted for 16 h. All experimental procedures were conducted according to the guidelines established in the “Guide for the Care and Use of Laboratory Animals” by the National Academy of Sciences^61^.

### Anti-inflammatory assay

The experiments adopted like essentially as described by Winter et al.^62^ Tween-80 (10%, *V/V*) was selected as vehicle to suspend the tested compounds and standard drug. Mice were divided into **15** groups each group containing 6 animals. The animals received 100 µl of vehicle or carrageenan (1% in saline) subcutaneous on the plantar surface of the left hind paw to induced edema. The first group was kept as control and was administered vehicle (1.0 mL), the second group (standard) was administrated celecoxib (50 mg/kg) orally as suspension in 10% Tween 80. The other remaining groups were administrated orally an aqueous suspension of the synthesized compounds (15 mg/kg) 1 h before carrageenan injection. The development of paw edema was assessed by measuring the changes in paw-volume with a mercury plethysmometer, before carrageenan injection and then hourly for 4 h post administration of the suspension of synthesized compound in 10% Tween 80. The right hind paw served as a reference of non-inflamed paw for comparison. Results are expressed as paw volume change (mL). Edema values, expressed as mean standard deviation SD, were compared statistically using one-way-ANOVA followed by Tukey-Kramer post hoc test and the *p* < 0.05 values were considered significant Table 2.

### Ulcerogenic liability study

The most biologically active synthesized compounds undergo ulcerogenic liability (**5b**, **5c**, **9**,** 11** and **12**) and celecoxib was evaluated^63^. Adult male albino rats weigh between 120 and 150 g were used in this study and divided into 7 groups. The animals were fasted for 20 h before drug administration. The first group control rats received P.O. the vehicle (suspension of 1% carboxy methylcellulose). The group from 2nd to 6th the synthesized compounds (**5b**, **5c**, **9**,** 11** and **12**) in a dose 200 mg/ kg suspended. The last group-received celecoxib used as a reference drug in a dose 200 mg/kg. Treatment was continued once daily for 3 successive days in all groups. Two hours after the last dose, rats were sacrificed under general anesthesia by using isoflurane used as inhalant anesthesia agent, and then the stomachs were removed, opened along the greater curvature and rinsed with saline. The gastric mucosa was examined for lesions in the form of hemorrhages or linear breaks and erosions. Stomachs exhibiting one or more ulcers were considered positive. The degree of ulcerogenic effect was expressed by dividing the percentage incidence of ulcers in each group of animals by ten.

### Statistical analysis

All statistical analyses were carried out using GraphPad Prism version 20 (GraphPad Software, La Jolla, CA, USA). Data were expresses as mean ± standard deviation (SD) One -way analysis of variance (ANOVA) utilized for multiple group comparisons by Tukey-Kramer post hoc test for multiple group comparisons. *P* < 0.05 was provided for statistical significant.

### Computational methods

All protein receptors were sourced from the RCSB database, as detailed in Table 5. The target protein structures were preprocessed with PyMOL software, which included the removal of water molecules, ions, and pre-existing ligands. The structures of the compounds were created using BIOVIA Draw. Subsequently, Open Babel^64^ was utilized to convert each compound into the mol2 format, followed by the use of Autodock tools to transform the molecules into the pdbqt format. Before docking, ligand-centered maps were produced using AutoDock Vina^65^. The 2D interactions between the targets and ligands were analyzed using the Discovery Studio program. The physicochemical parameters and ADMET compounds were calculated using the ADMETLab 2.0: server tool^66^.

**Table 5:** Targets of anticancer proteins, PDB IDs, active site coordinates, and reference ligands.

Microorganism	Protein targets	PDB ID	Resolutions	Active sit coordinates:			Reference
				X	Y	Z	
*B. subtilis*	GyraseB	**4URM**	2.94 Å	19.96	5.08	20.12	Levofloxacin
*E.coli*	DNA Gyrase	**7P2M**	1.90 Å	27.52	63.35	-2.29	Levofloxacin
*K. pneumoniae*	KPC-2 carbapenemase	**2OV5**	1.80 Å	72.07	36.33	11.54	Levofloxacin
*S. aureus*	dihydropteroate synthase	**1AD4**	2.00 Å	19.96	5.08	20.12	Levofloxacin
*S. pneumonia*	Sortase A (spySrtA)	**8T8G**	1.50 Å	27.52	63.35	-2.29	Levofloxacin
*S. typhimurium*	Neuraminidase	**2SIL**	1.60 Å	72.07	36.33	11.54	Levofloxacin

### Molecular dynamics (MD) simulation

Molecular dynamics (MD) simulations are widely used to investigate the binding interactions and affinities within protein-ligand complexes. In this research, MD simulations were carried out using GROMACS 2018 software to confirm the accuracy and consistency of the docking results. Protein topology was developed with the CHARMM36 force field parameters, and the compound topologies were created via the Geoff server. Ligands were constrained at their coordinate positions. NVT and NPT equilibrations were conducted for 1000 ps at 300 K and 1.0 bar pressure. After the MD simulations, key metrics such as Root Mean Square Deviation (RMSD), Root Mean Square Fluctuation (RMSF), and radius of gyration (Rg) were analyzed^67^.

## Conclusion

This study presents a novel series of pyrazole-chalcone hybrids characterized by their dual antibacterial and anti-inflammatory activities. The key novelty lies in the strategic integration of antipyrine, thieno– and pyridopyrimidine moieties with the pyrazole core, yielding compounds **4c**, **5c**, and **12** that not only surpass the potency of standard drugs like Levofloxacin and Celecoxib in specific assays but also exhibit a significantly improved safety profile, as evidenced by reduced ulcerogenicity. The consistent biological potency is robustly supported by in-silico studies, which confirm strong target binding and favorable drug-like properties, particularly for lead compound **4c**. These findings underscore the translational potential of these hybrids as promising candidates for further development as multi-target therapeutic agents against drug-resistant infections and associated inflammatory conditions. Future work will focus on in vivo efficacy studies and further lead optimization based on the established SAR and ADMET profiles.

## Supplementary Information

Below is the link to the electronic supplementary material.

Supplementary Material 1

Supplementary Material 2

## Data Availability

The data that support the finding of this study are available in the supplementary materials of this article.
