# Supplementary material for: Design, synthesis, characterization, pharmacological evaluation and in silico ADMET and molecular docking and dynamics simulations of a novel series of N-substituted pyrazole from chalcone derivatives
Source: Sci Rep. 2026 Mar 1;16:7931. doi: 10.1038/s41598-026-38237-9 (PMC12953870; doi:10.1038/s41598-026-38237-9)
Supplement: Supplementary file 1 — Supplementary Material 1 [file 41598_2026_38237_MOESM1_ESM.pdf]

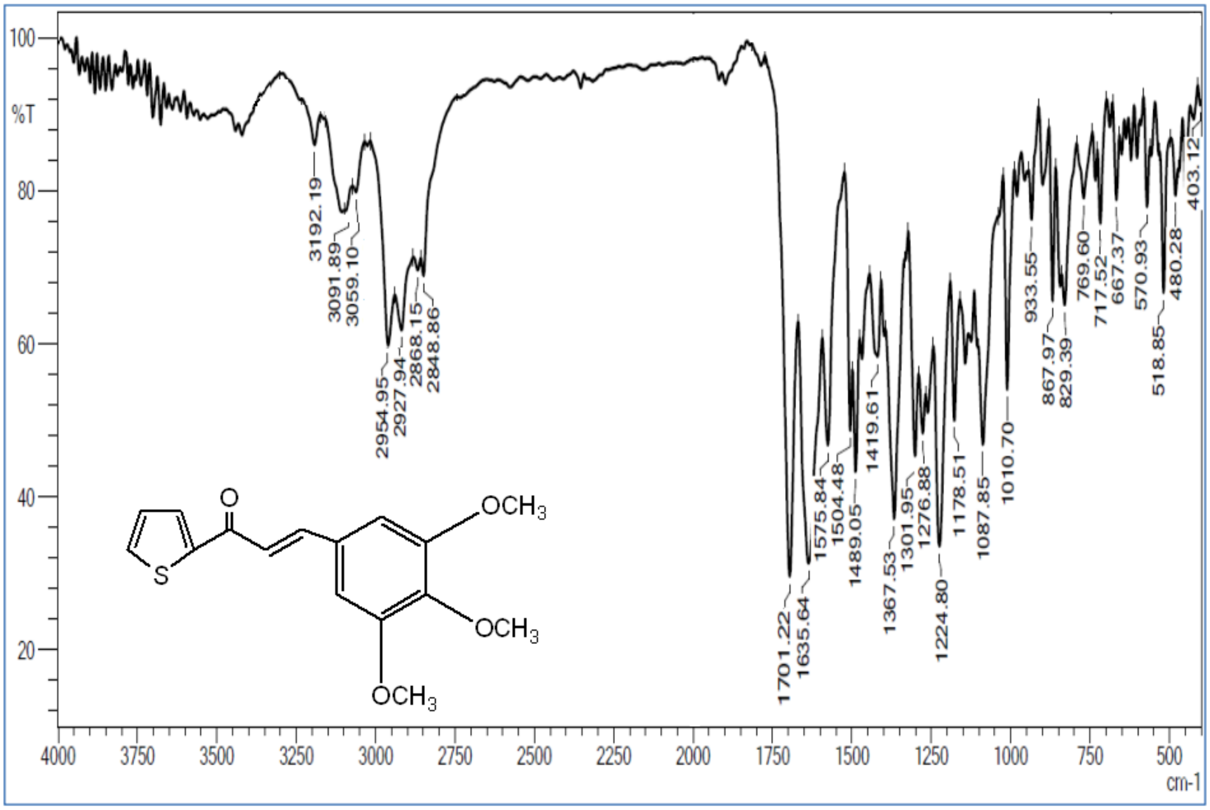

Fig.(1a). IR spectrum of compound **(3a)**

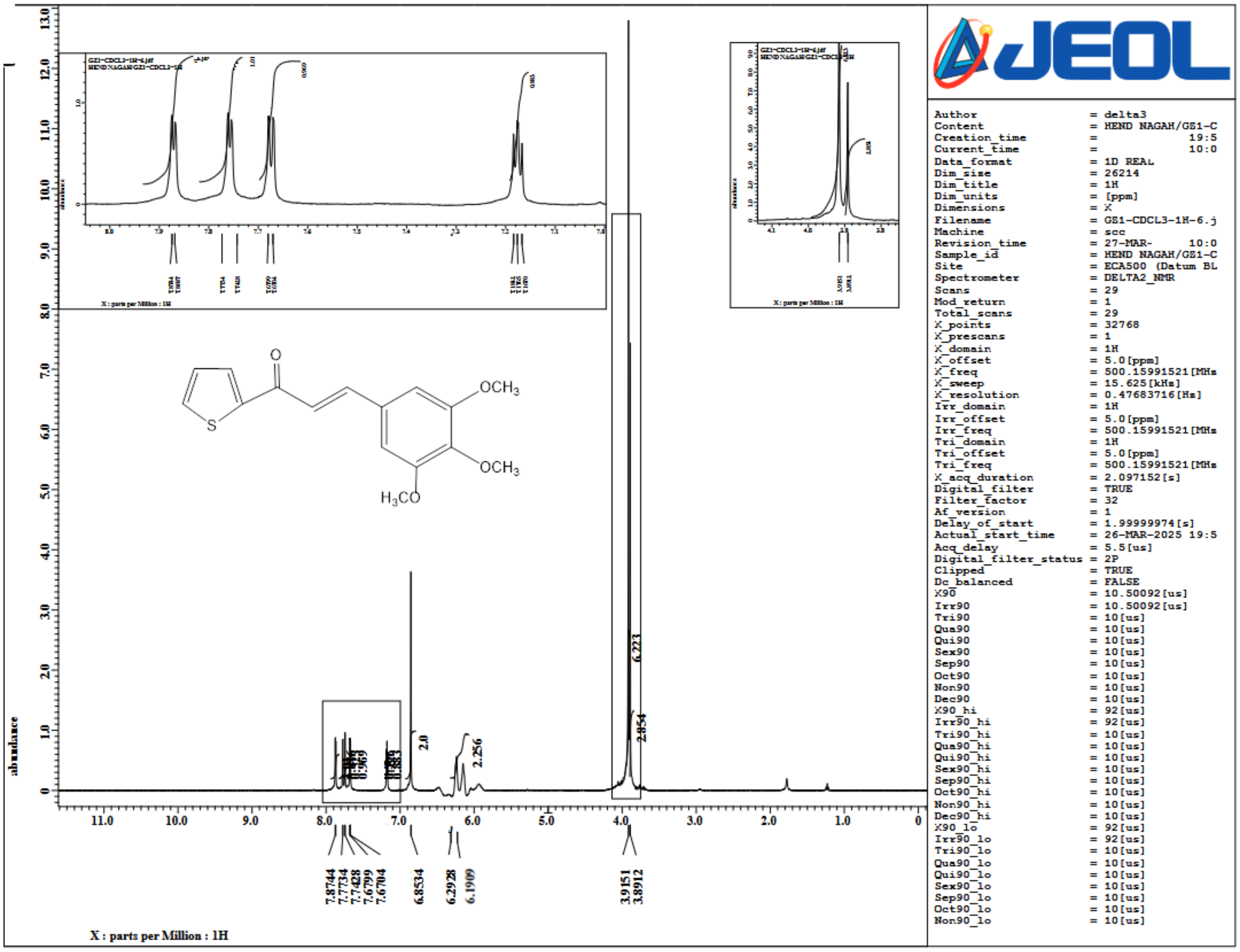

Fig. (1b) ^{1}H NMR spectrum of compound (3a)

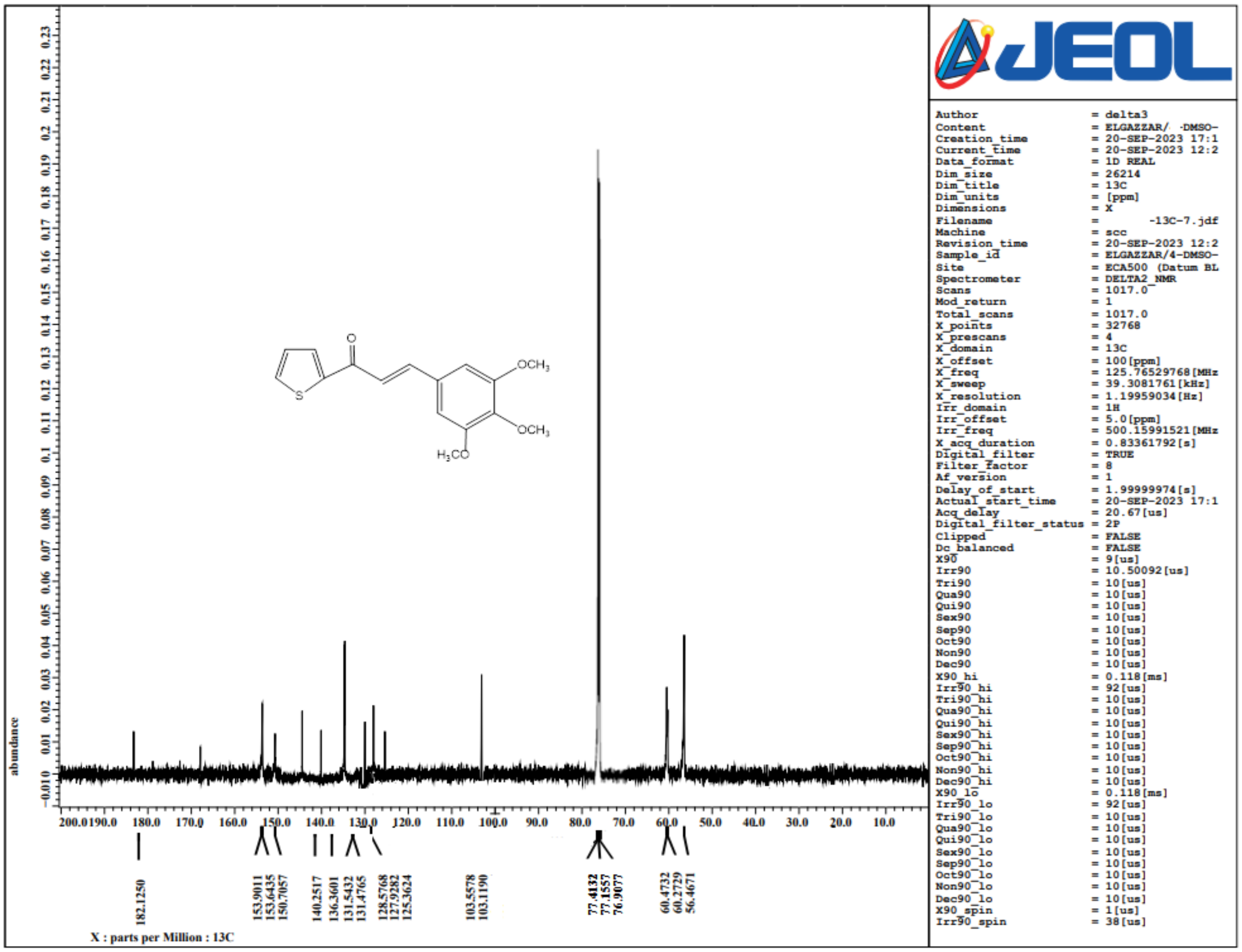

Fig. (1c). ^{13}C- NMR spectrum of compound **(3a)**

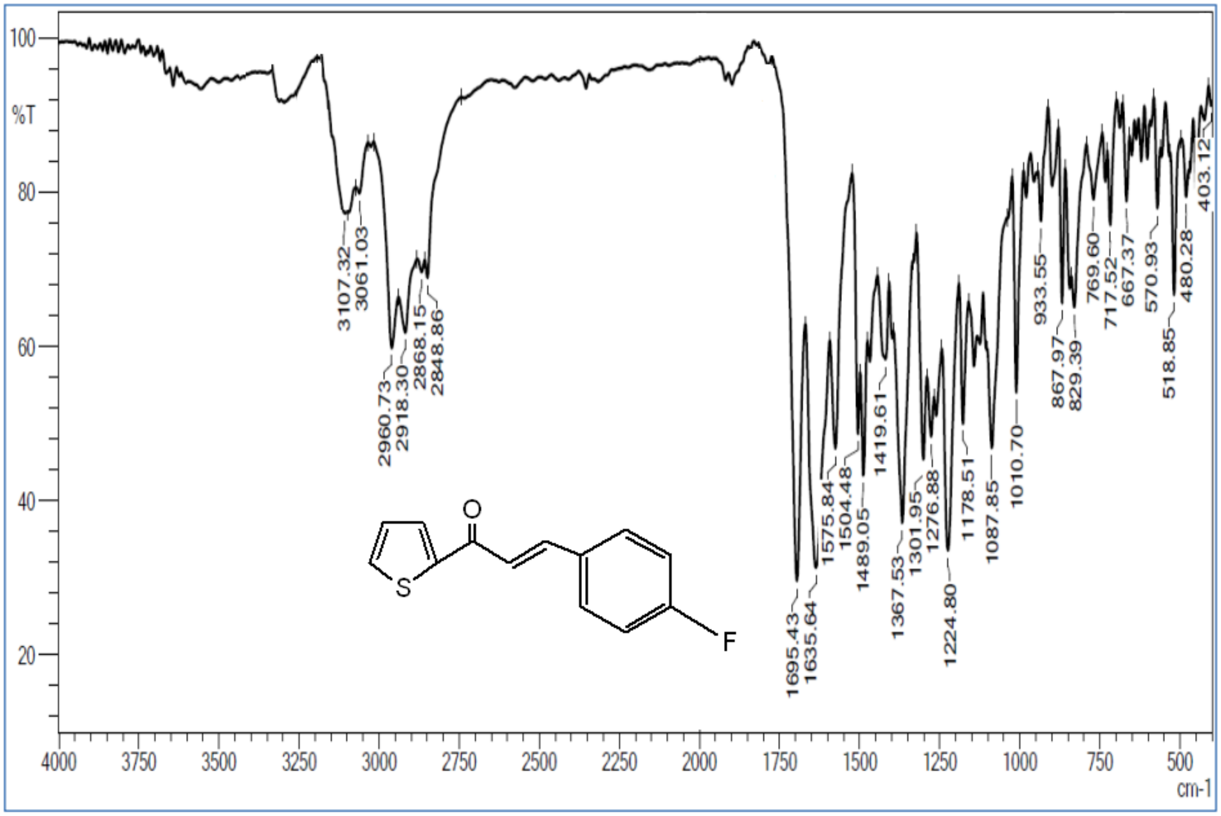

Fig.(2a). IR spectrum of compound **(3b)**

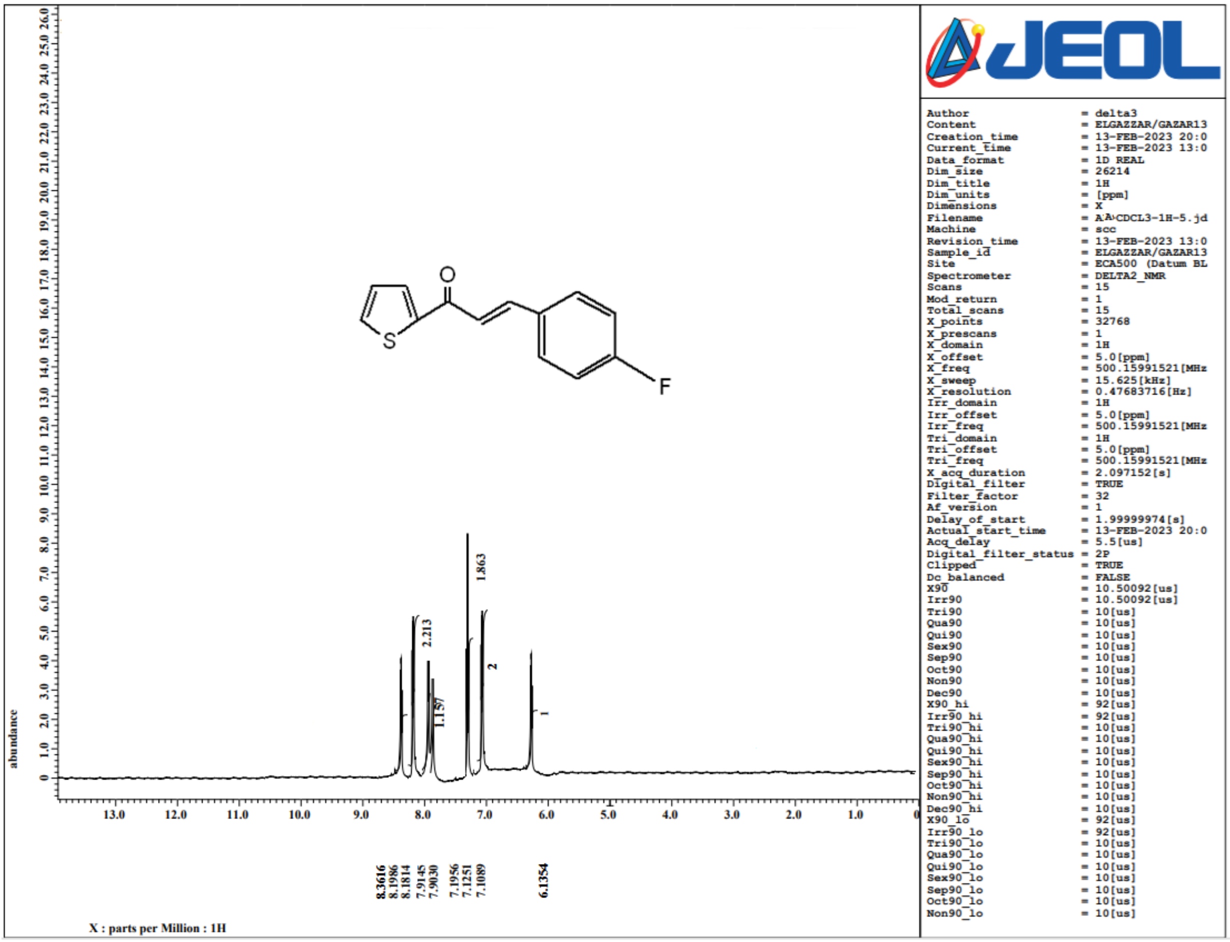

Fig.(2b). **^{1}H NMR** spectrum of compound **(3b)**

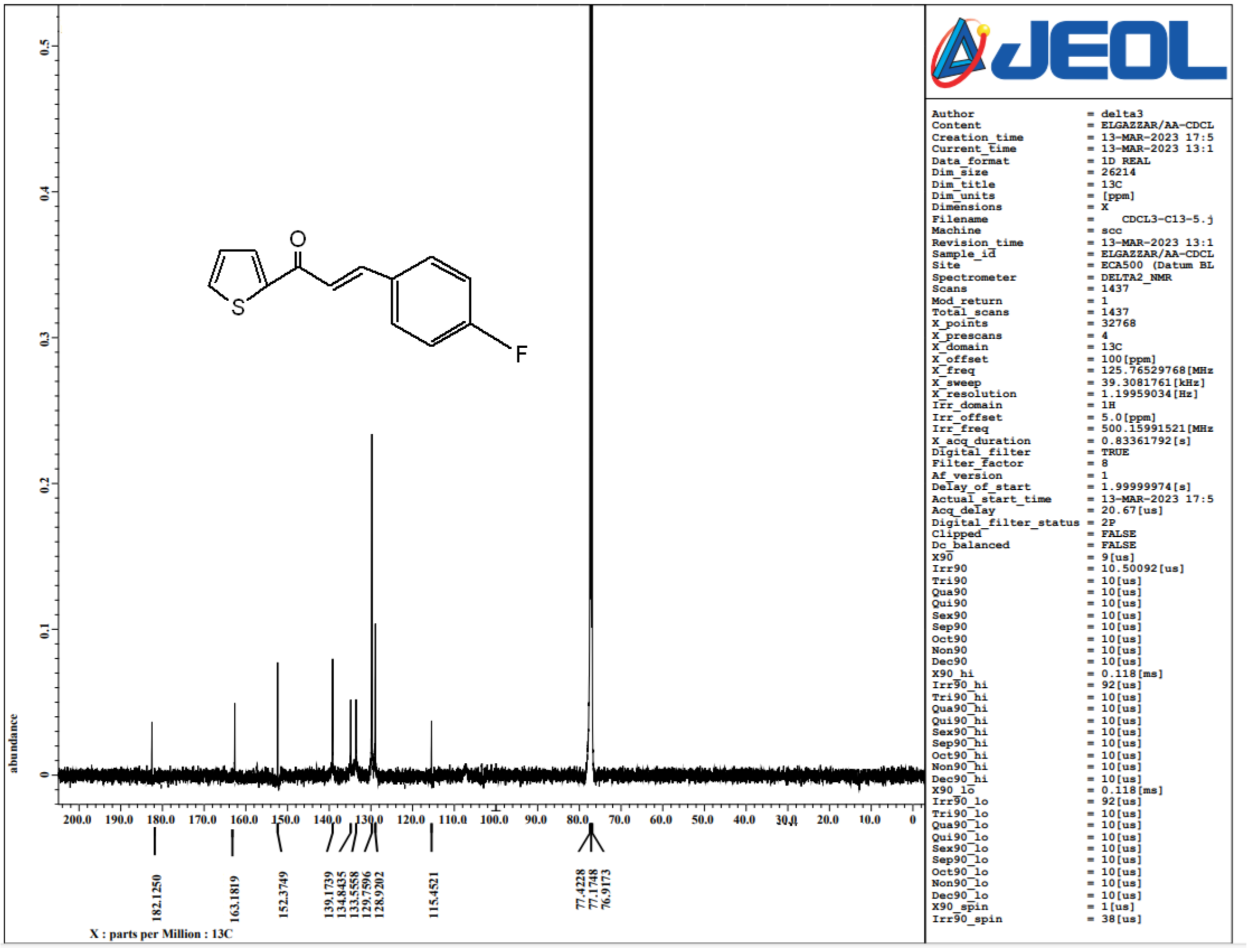

Fig.(2b). ^{13}C- NMR spectrum of compound **(3b)**

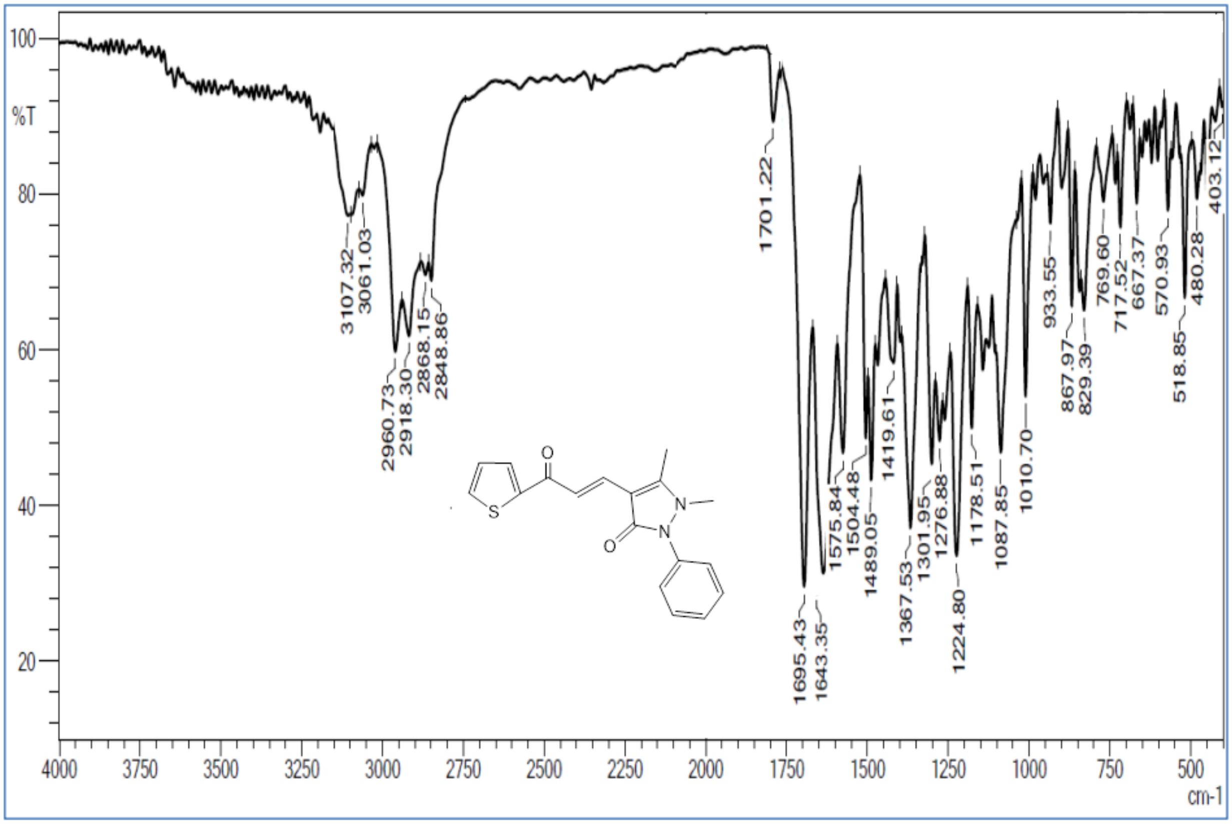

Fig.(3a). IR spectrum of compound **(3c)**

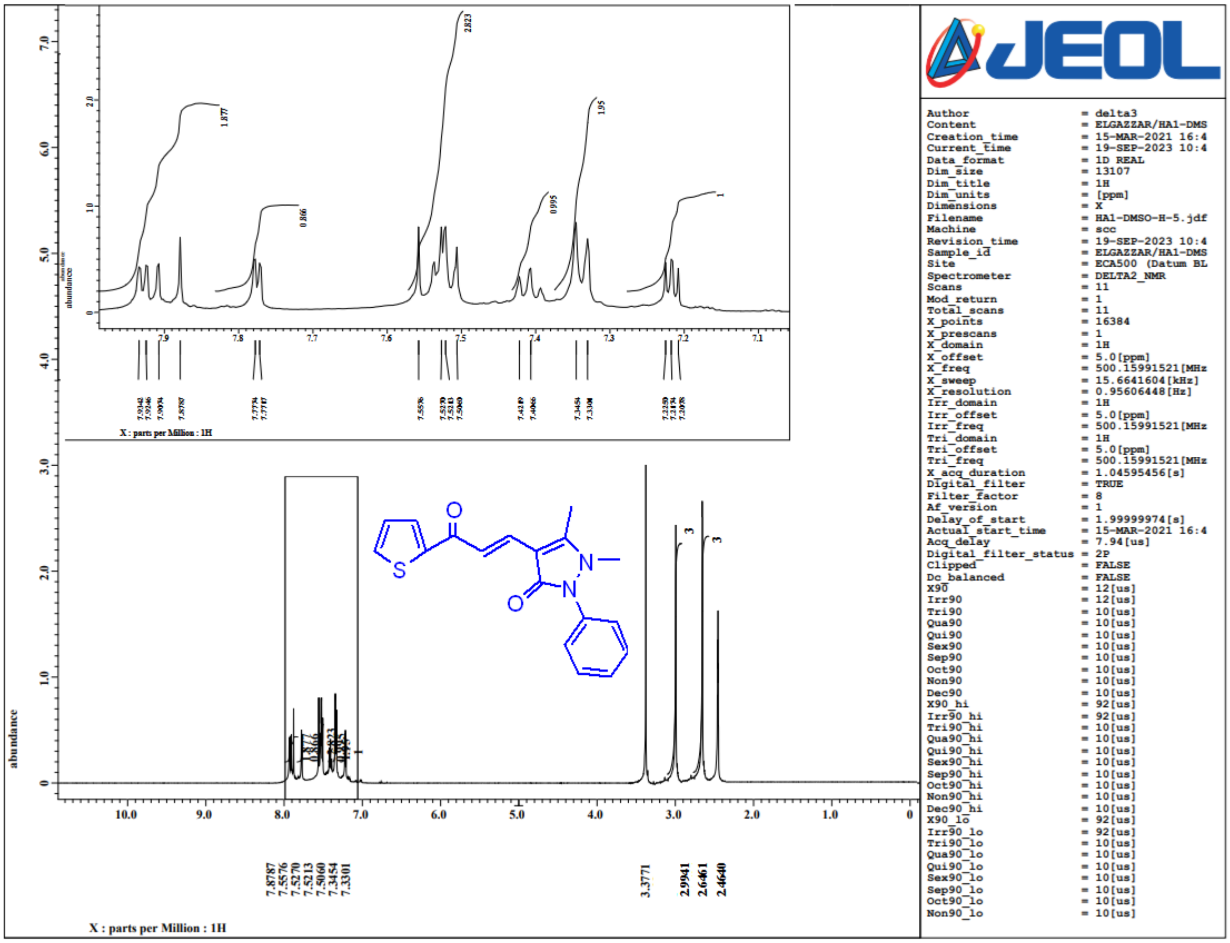

Fig.(3b). **^{1}H NMR** spectrum of compound **(3c)**

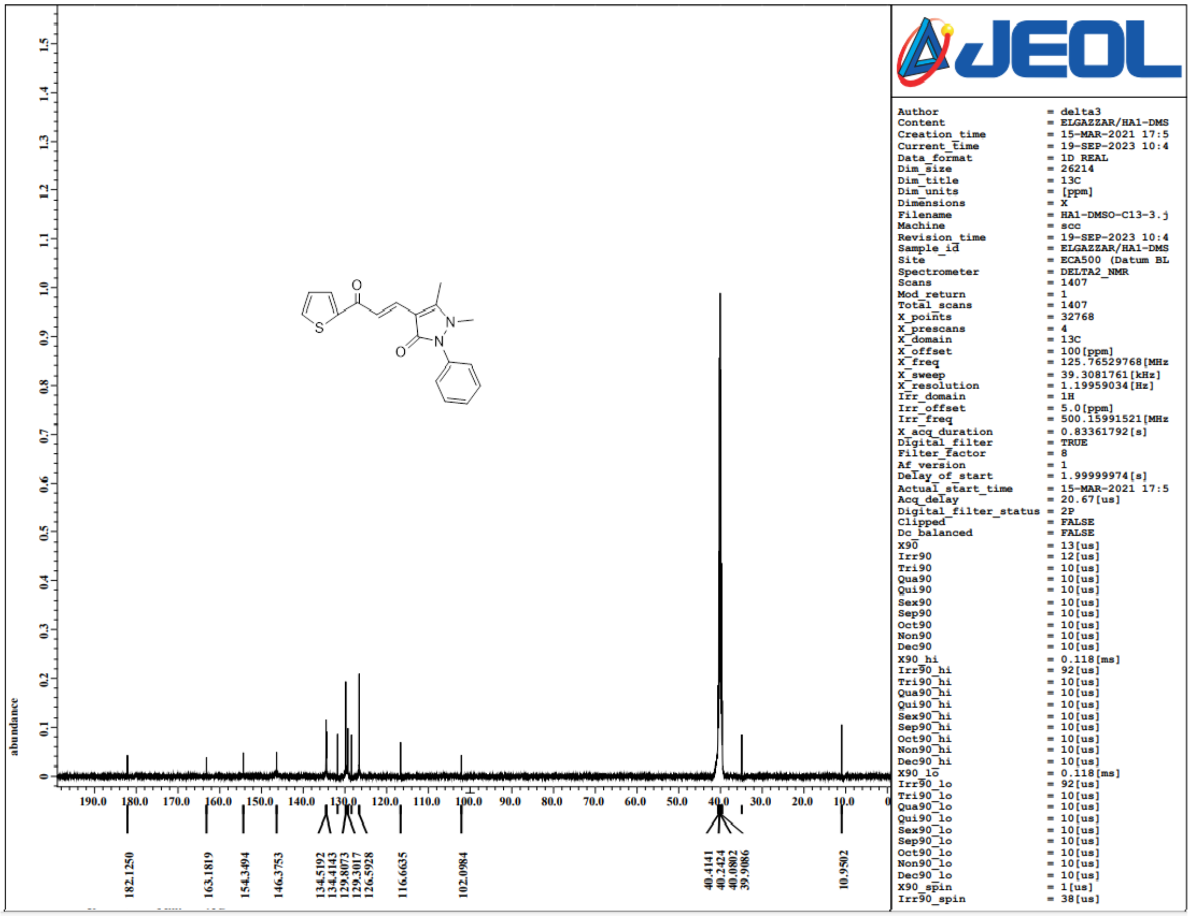

Fig.(3c). ^{13}C- NMR spectrum of compound **(3c)**

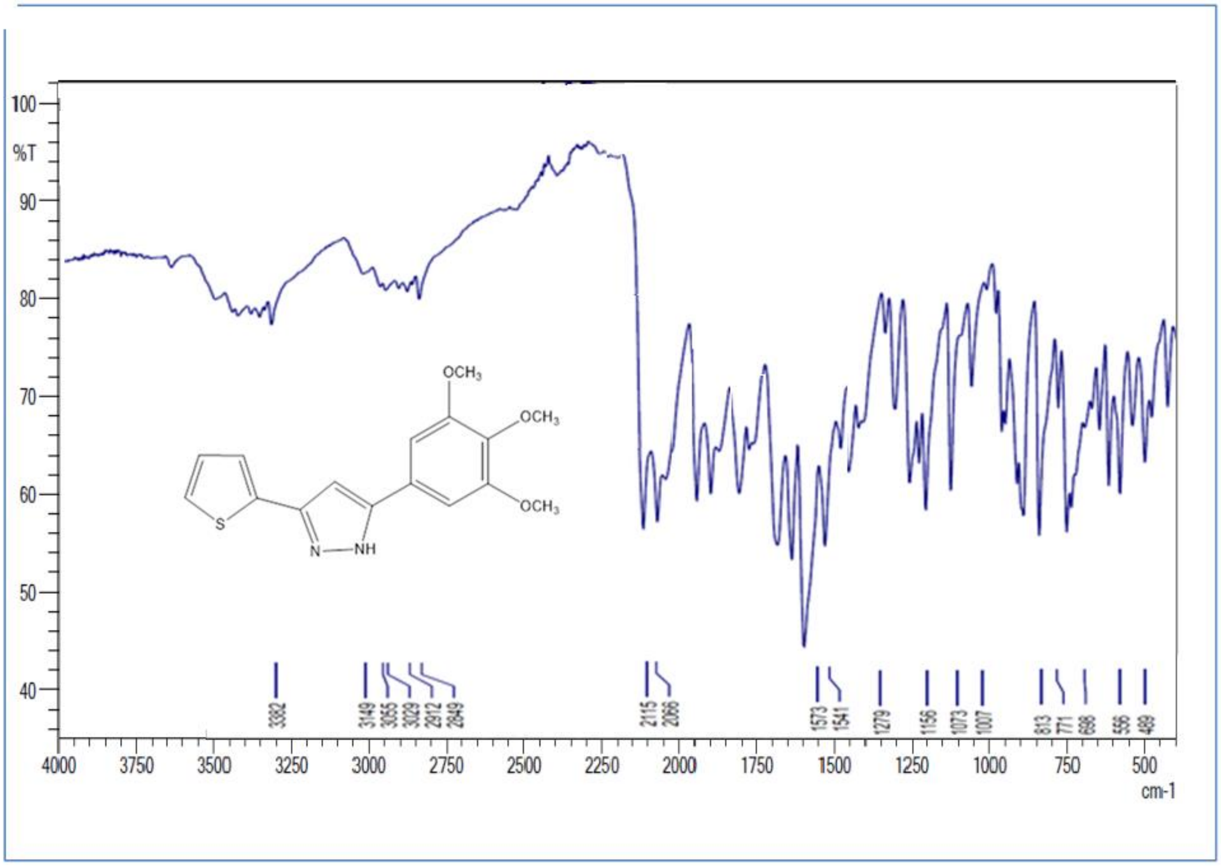

Fig.(4a). IR spectrum of compound **(4a)**

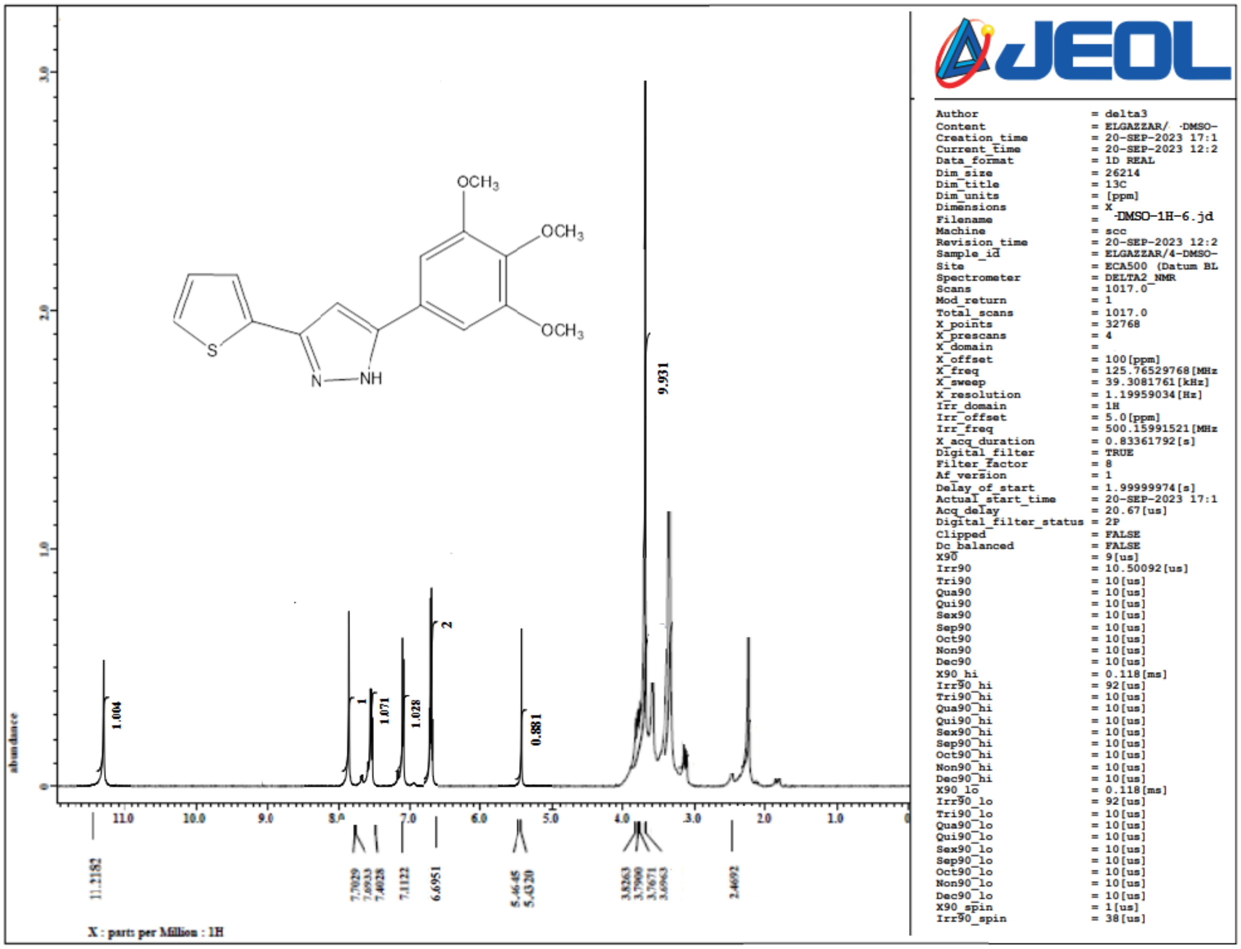

Fig.(4b). [1]**H NMR** spectrum of compound **(4a)**

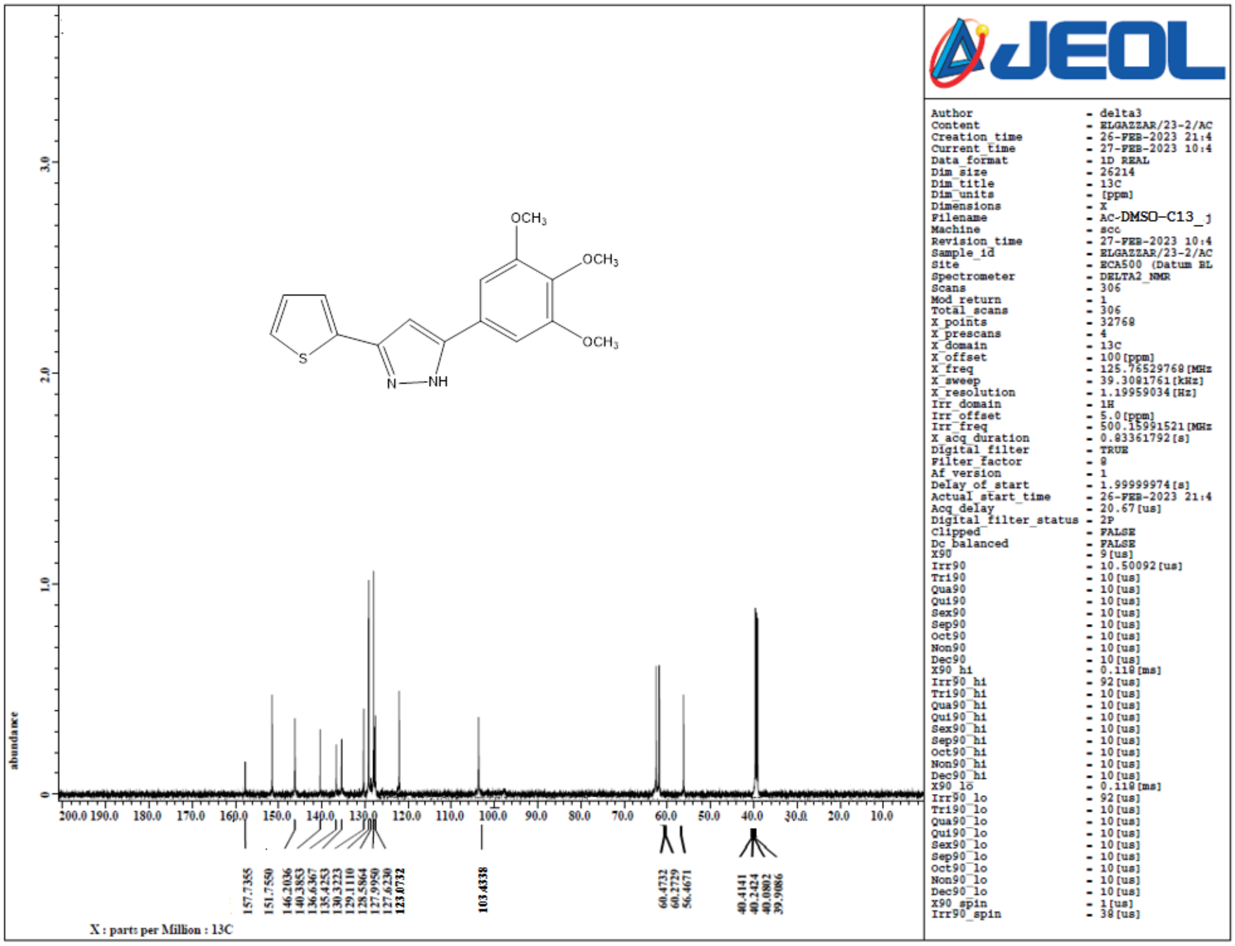

Fig.(4c). ^{13}C- NMR spectrum of compound **(4a)**

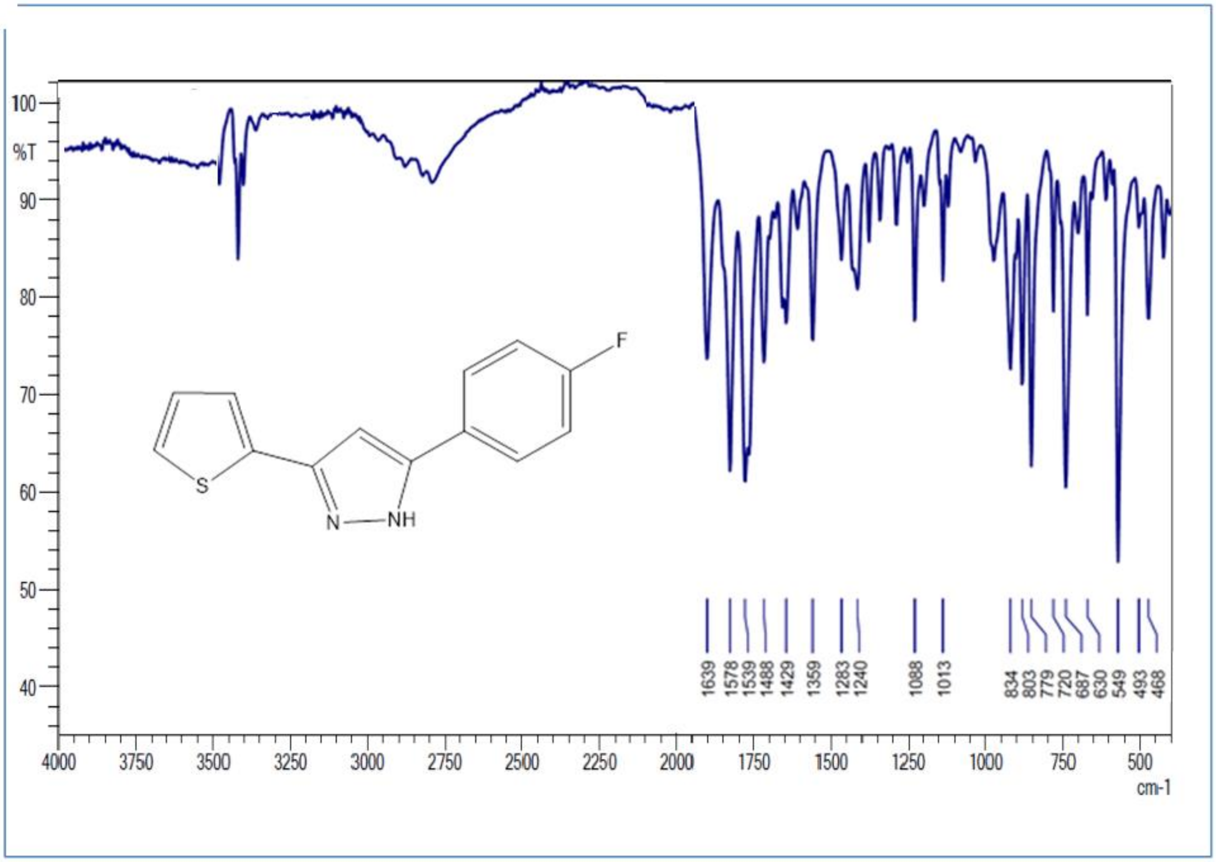

Fig.(5a). IR spectrum of compound **(4b)**

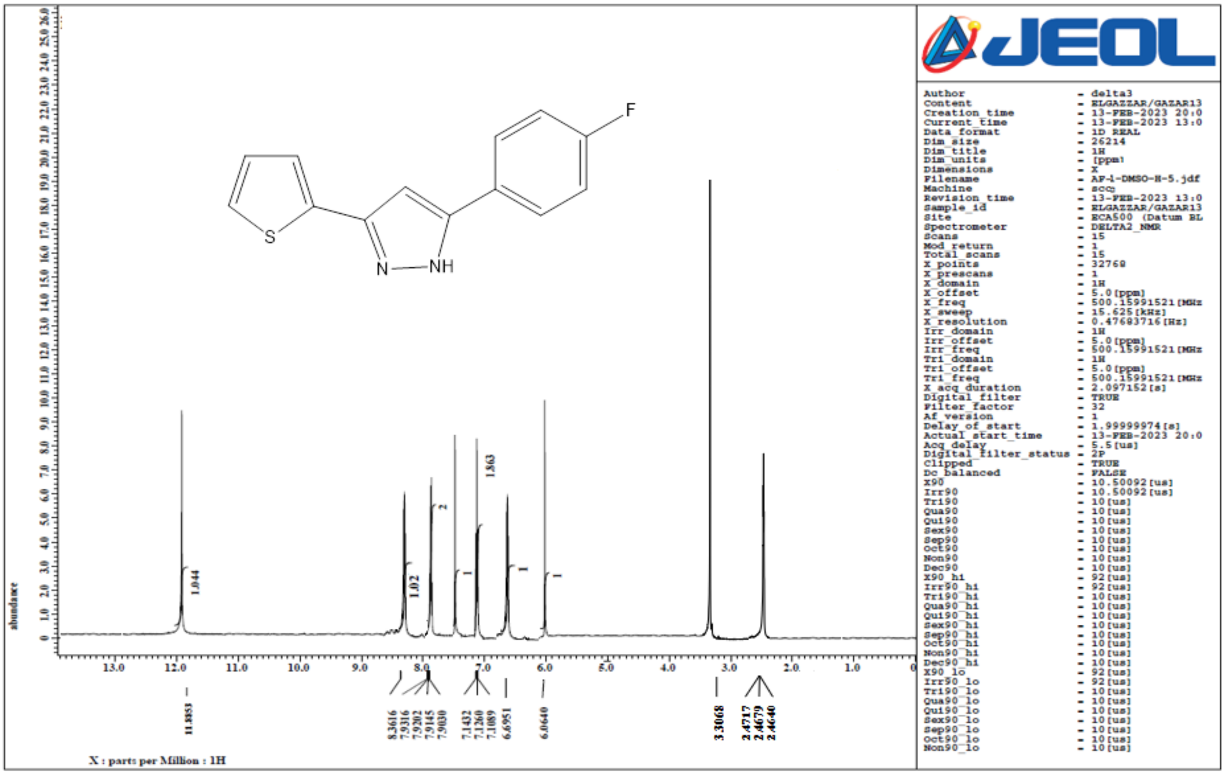

Fig.(5b). **¹H NMR** spectrum of compound **(4b)**

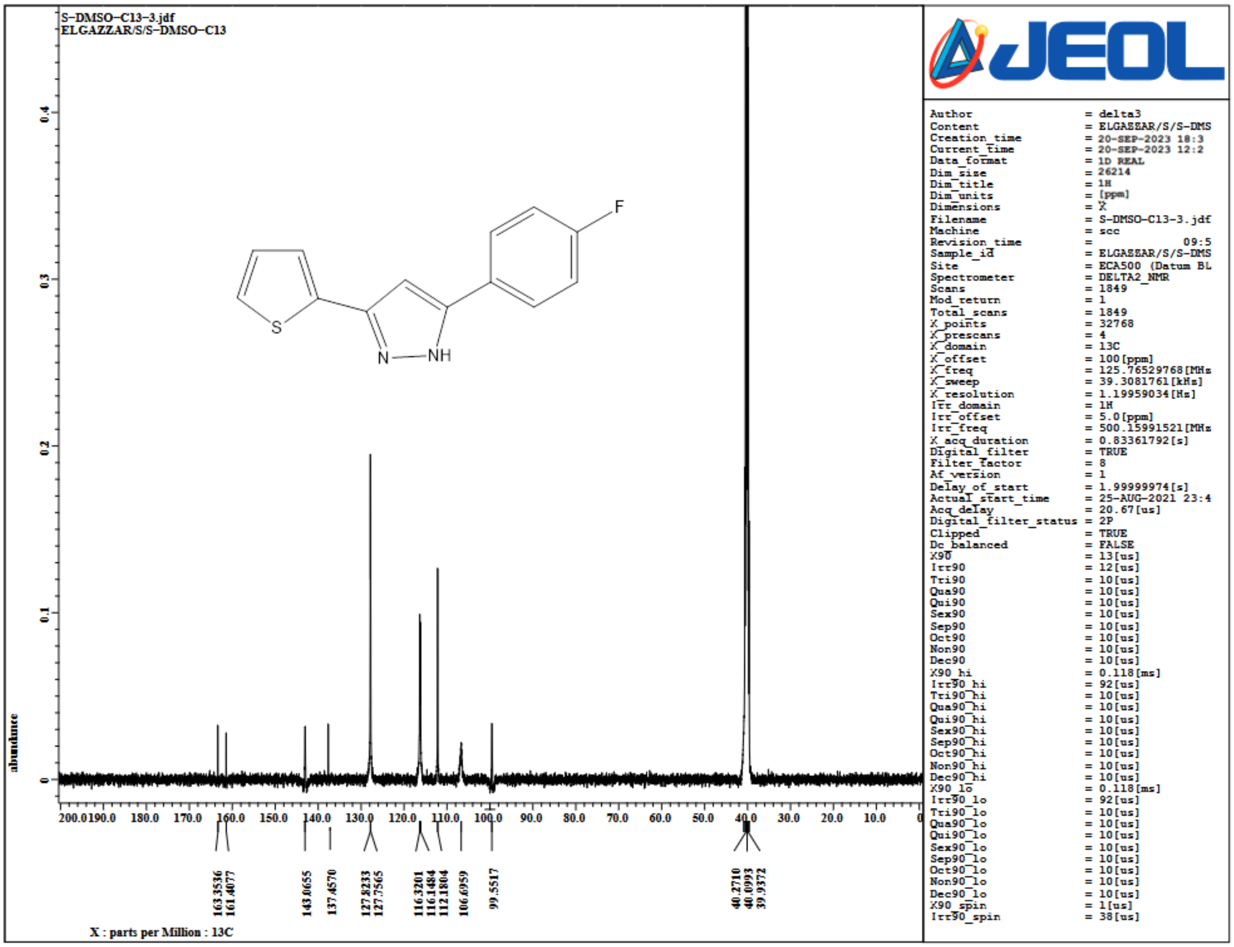

Fig.(5c). ^{13}C- NMR spectrum of compound **(4b)**

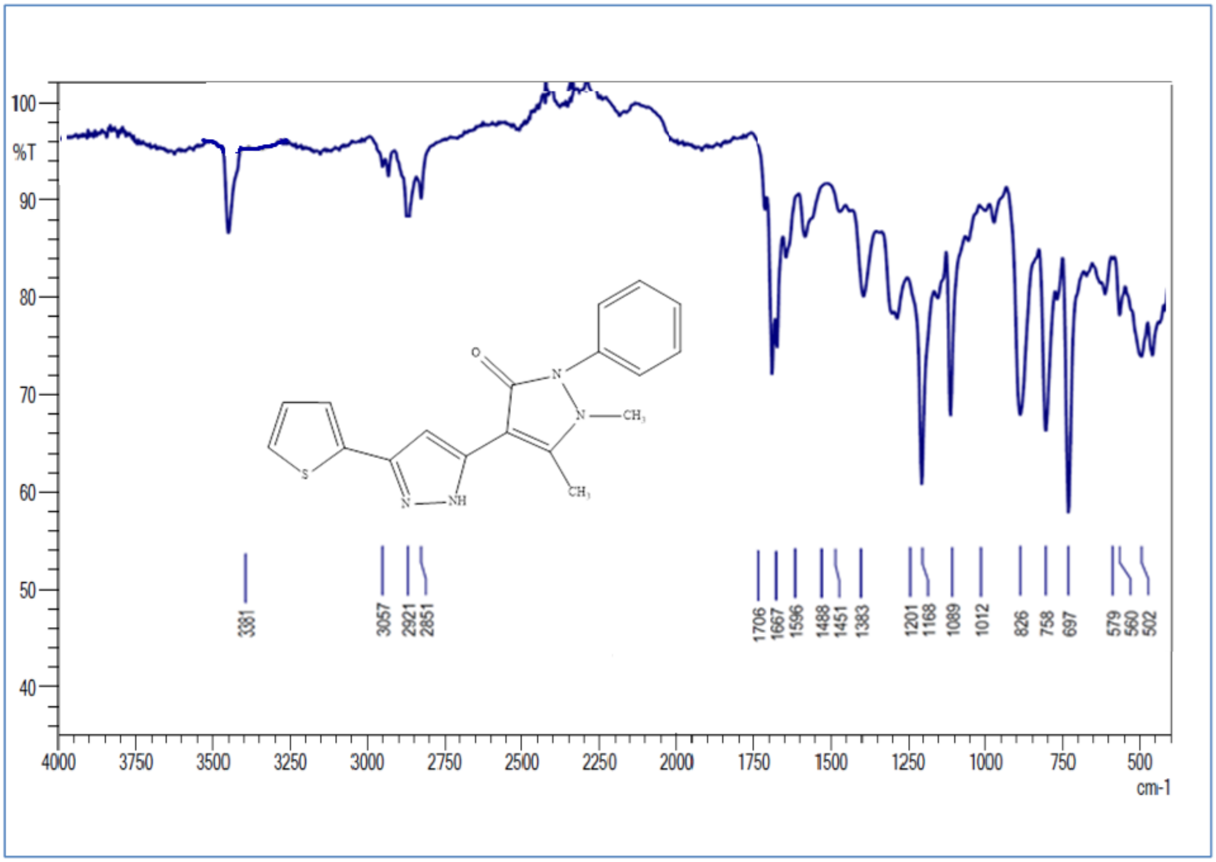

Fig.(6a). IR spectrum of compound **(4c)**

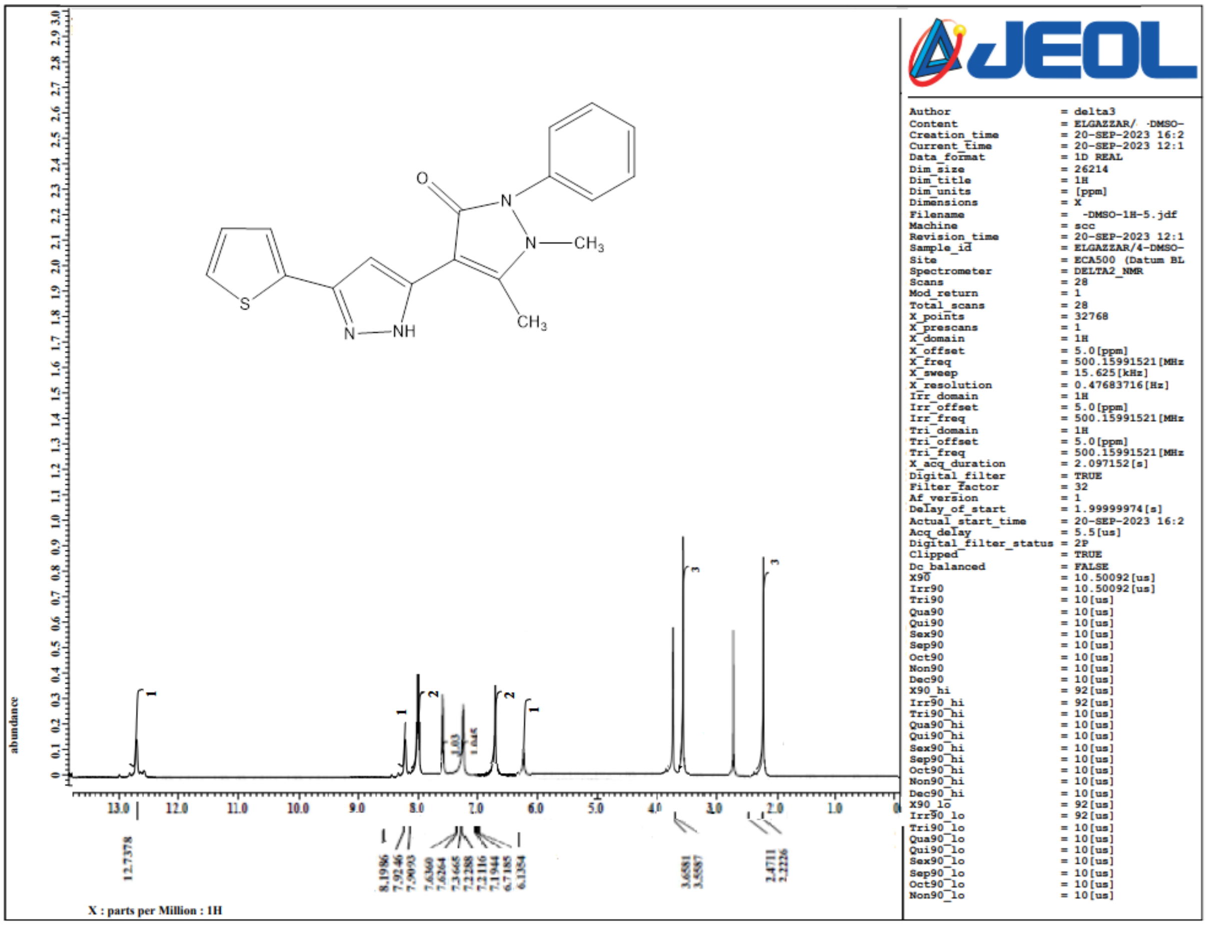

Fig.(6b). [1]**H NMR** spectrum of compound **(4c)**

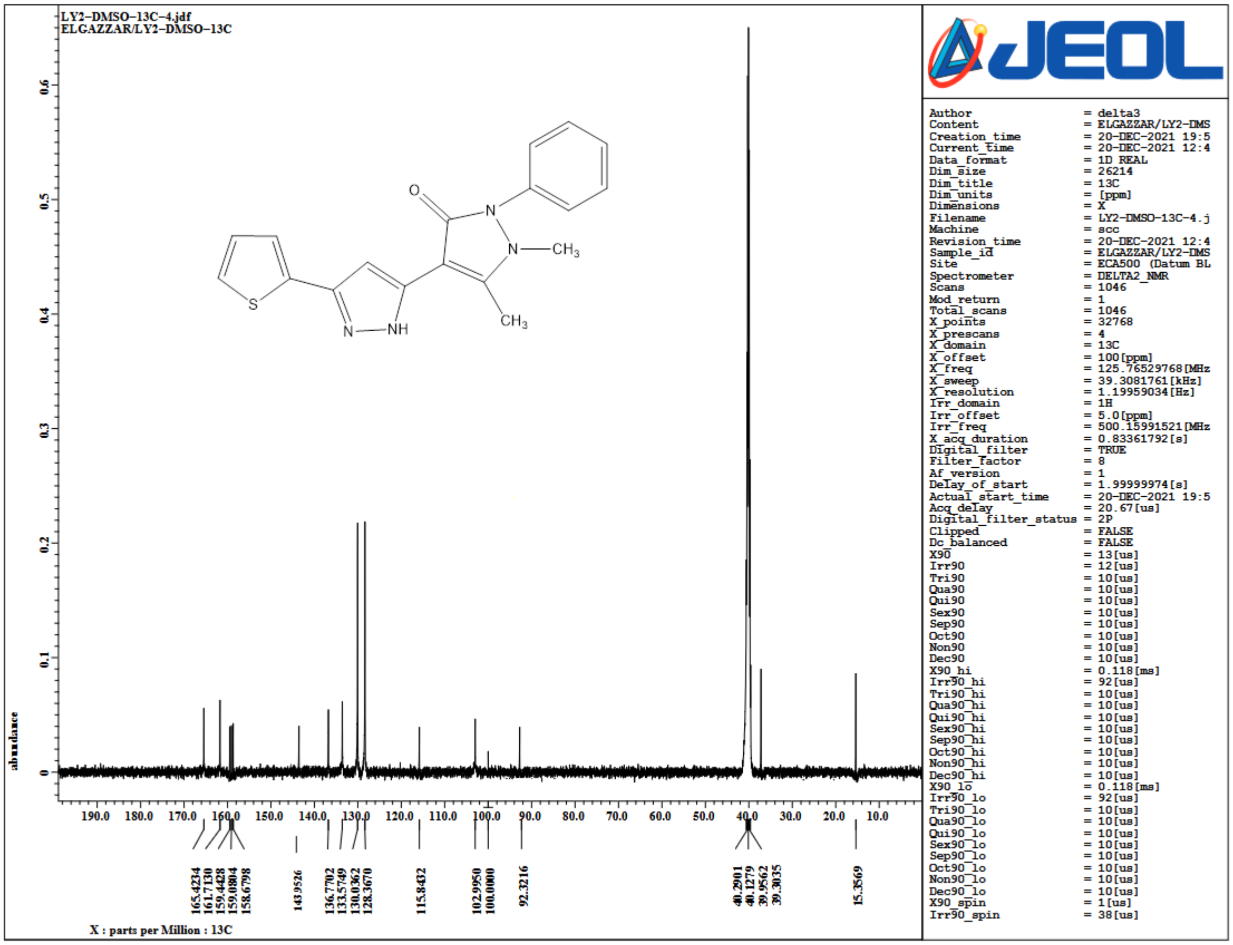

Fig.(6c). ^{13}C- NMR spectrum of compound **(4c)**

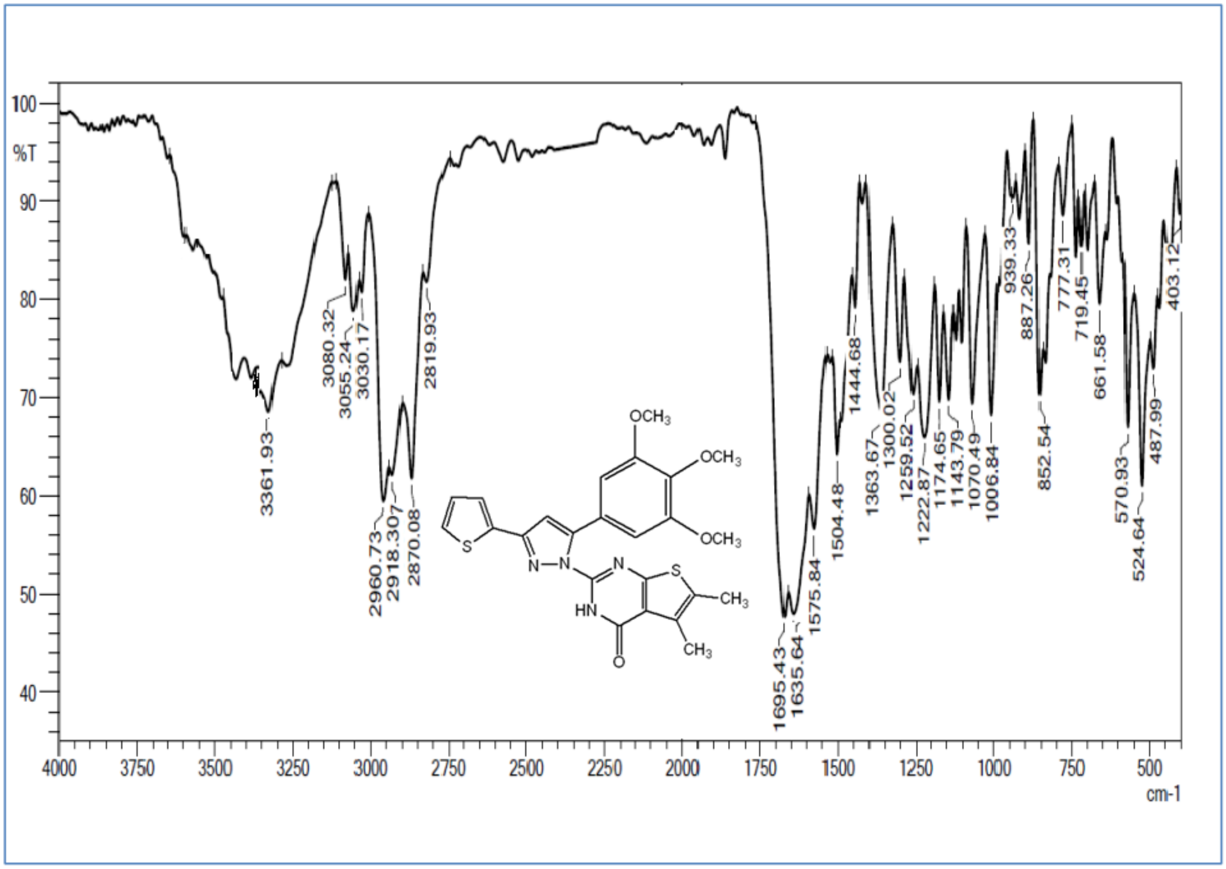

Fig.(7a). IR spectrum of compound **(5a)**

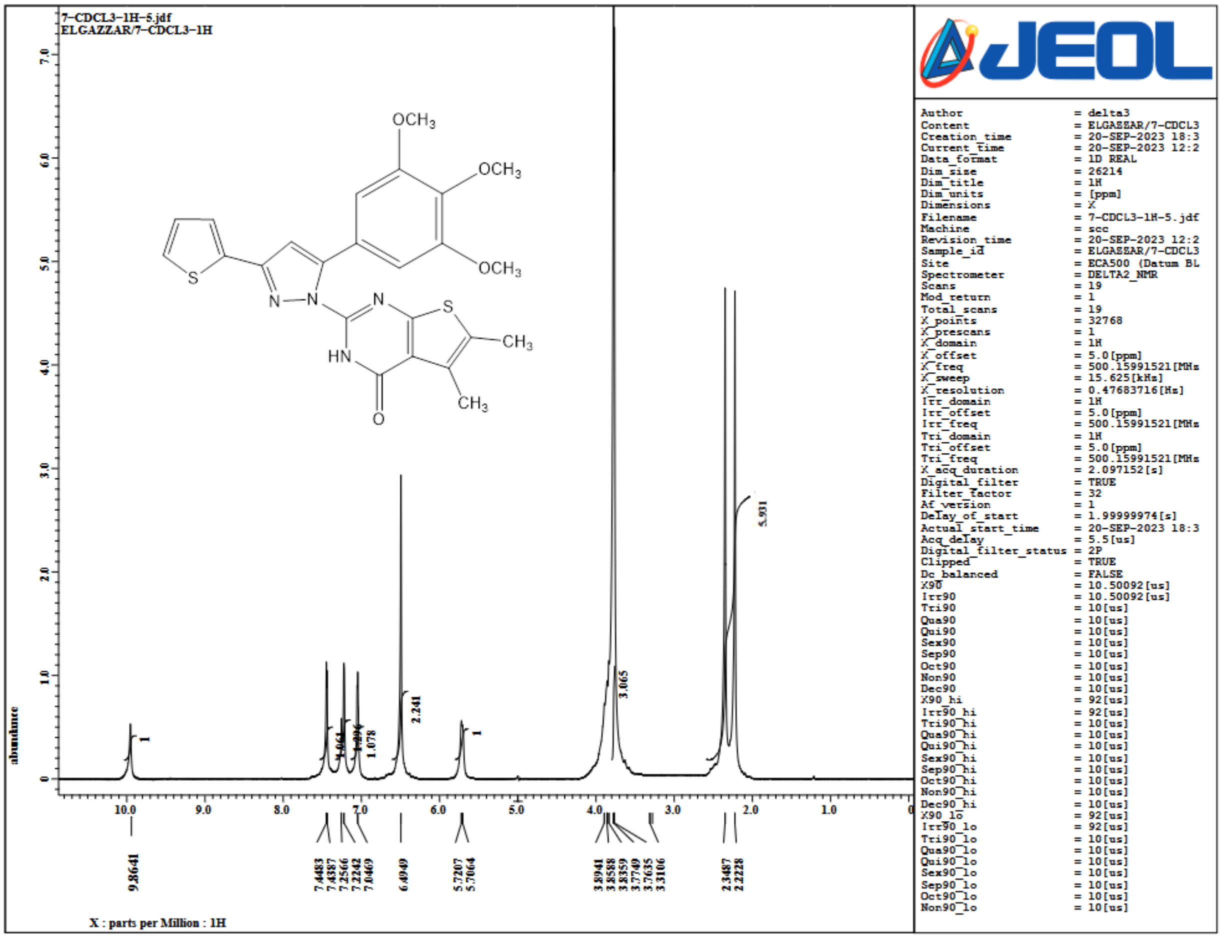

Fig.(7b). ^{1}**H NMR** spectrum of compound **(5a)**

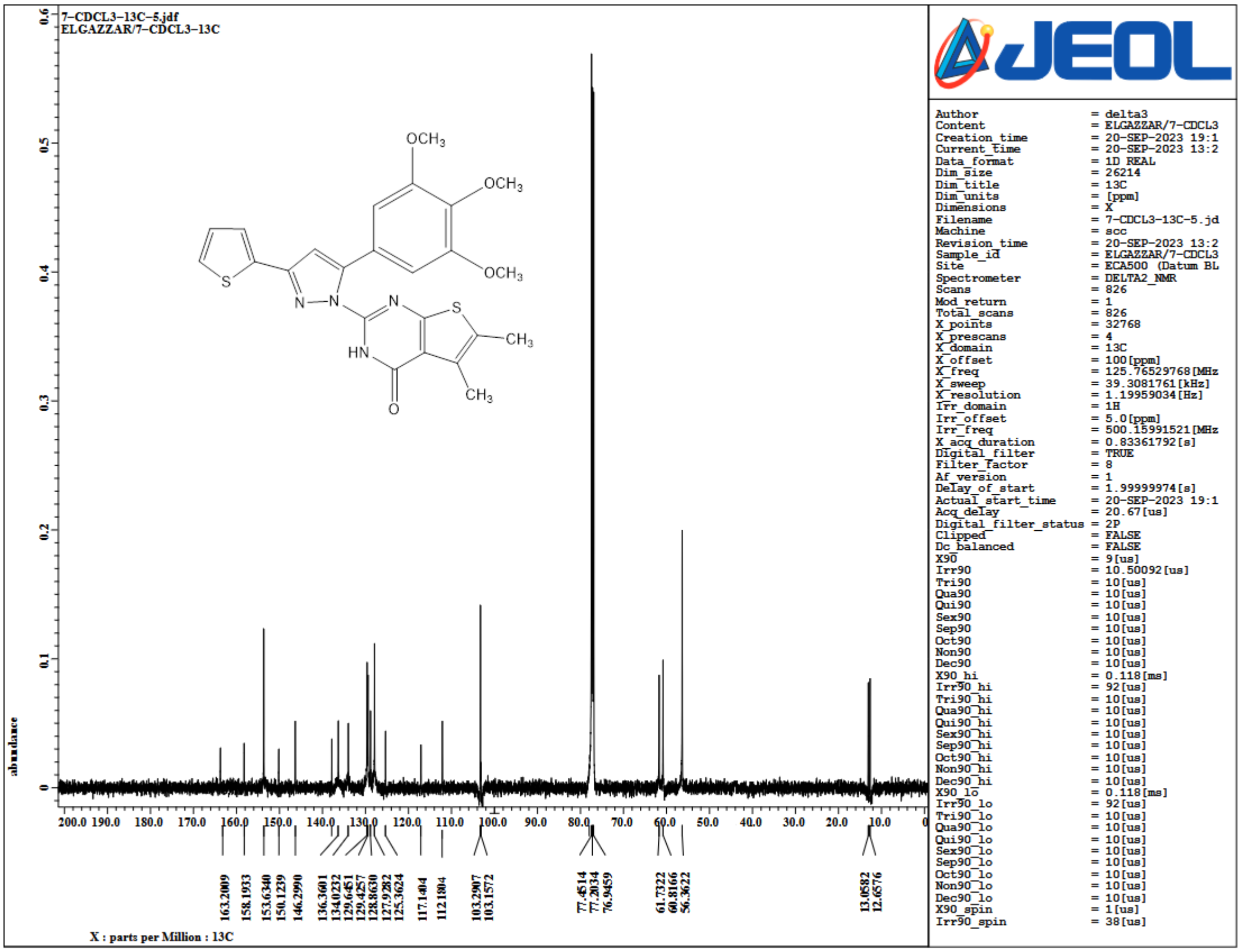

Fig.(7c). ^{13}C- NMR spectrum of compound **(5a)**

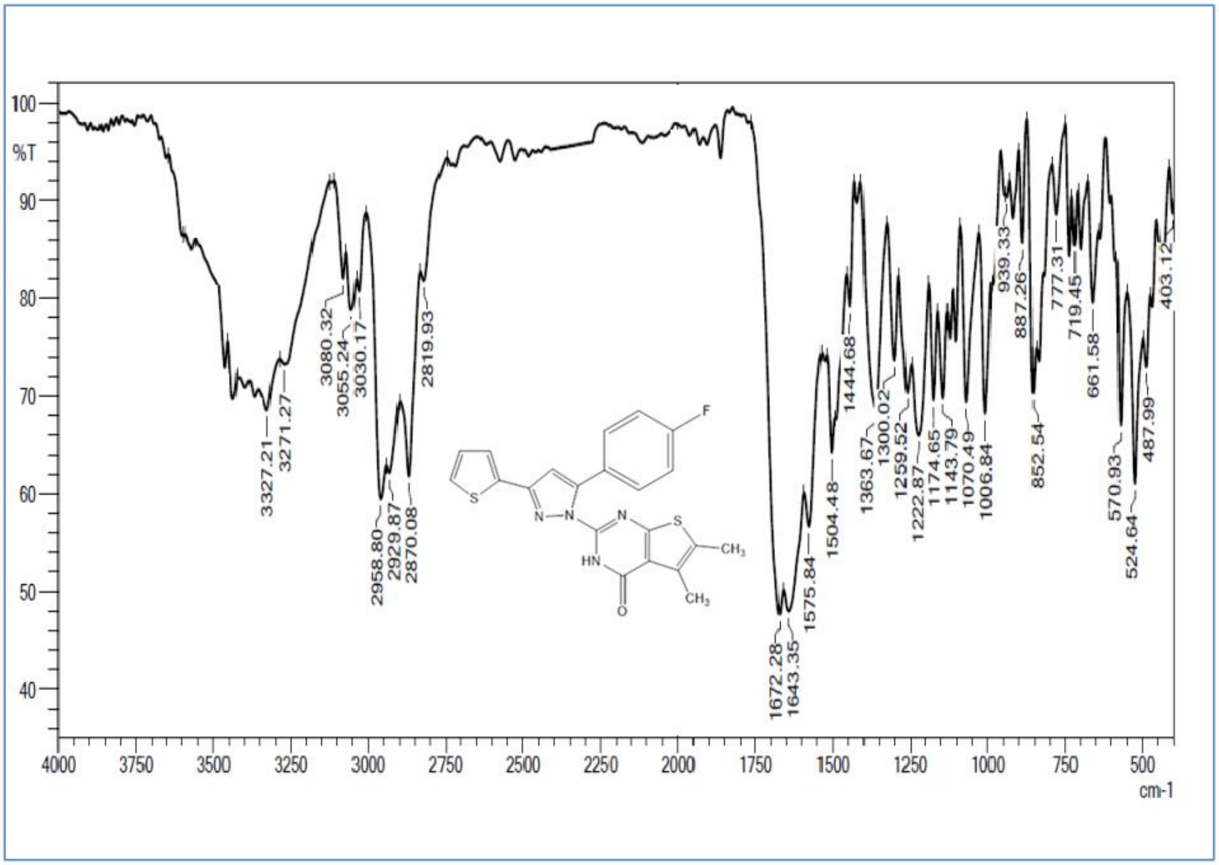

Fig.(8a). IR spectrum of compound **(5b)**

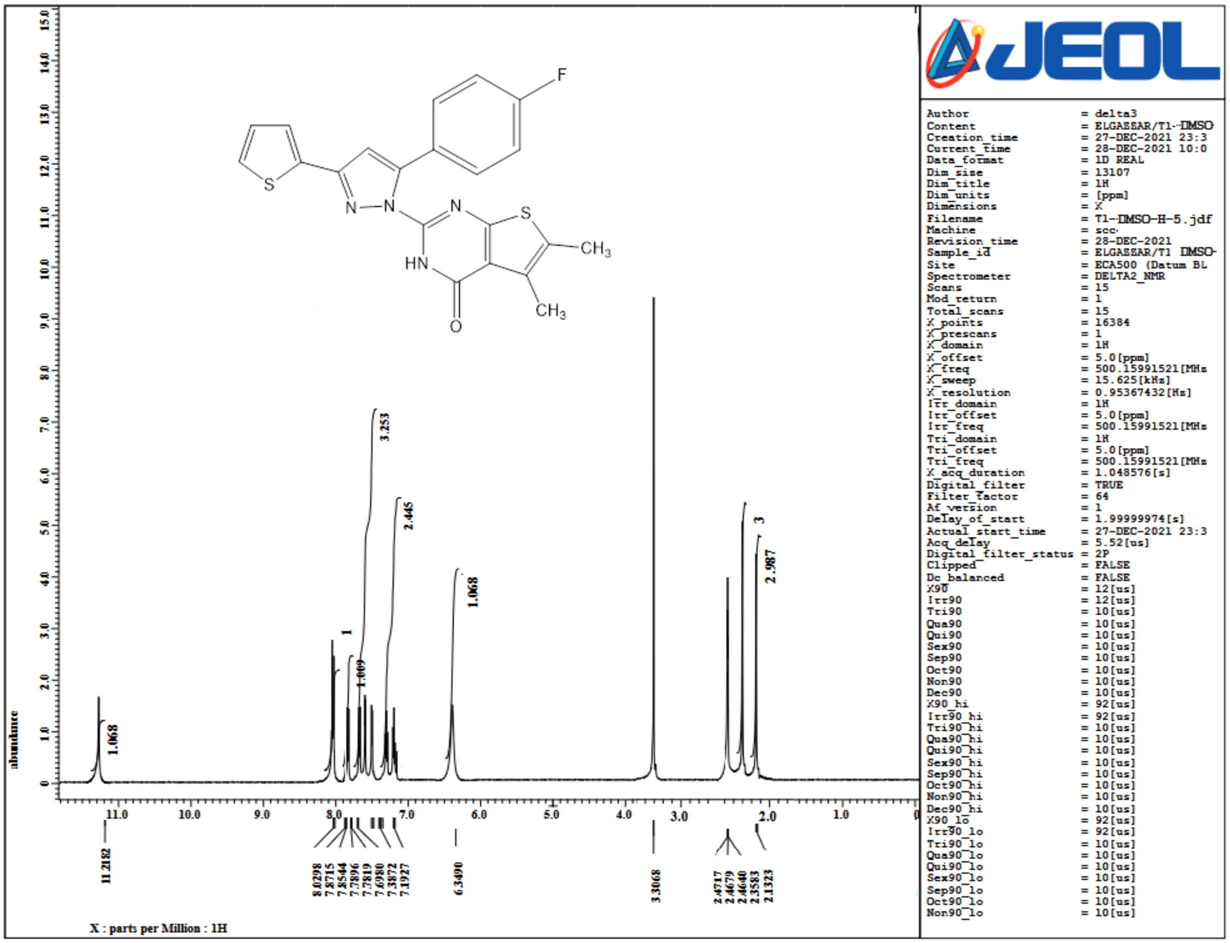

Fig.(8b). ^{1}H NMR spectrum of compound **(5b)**

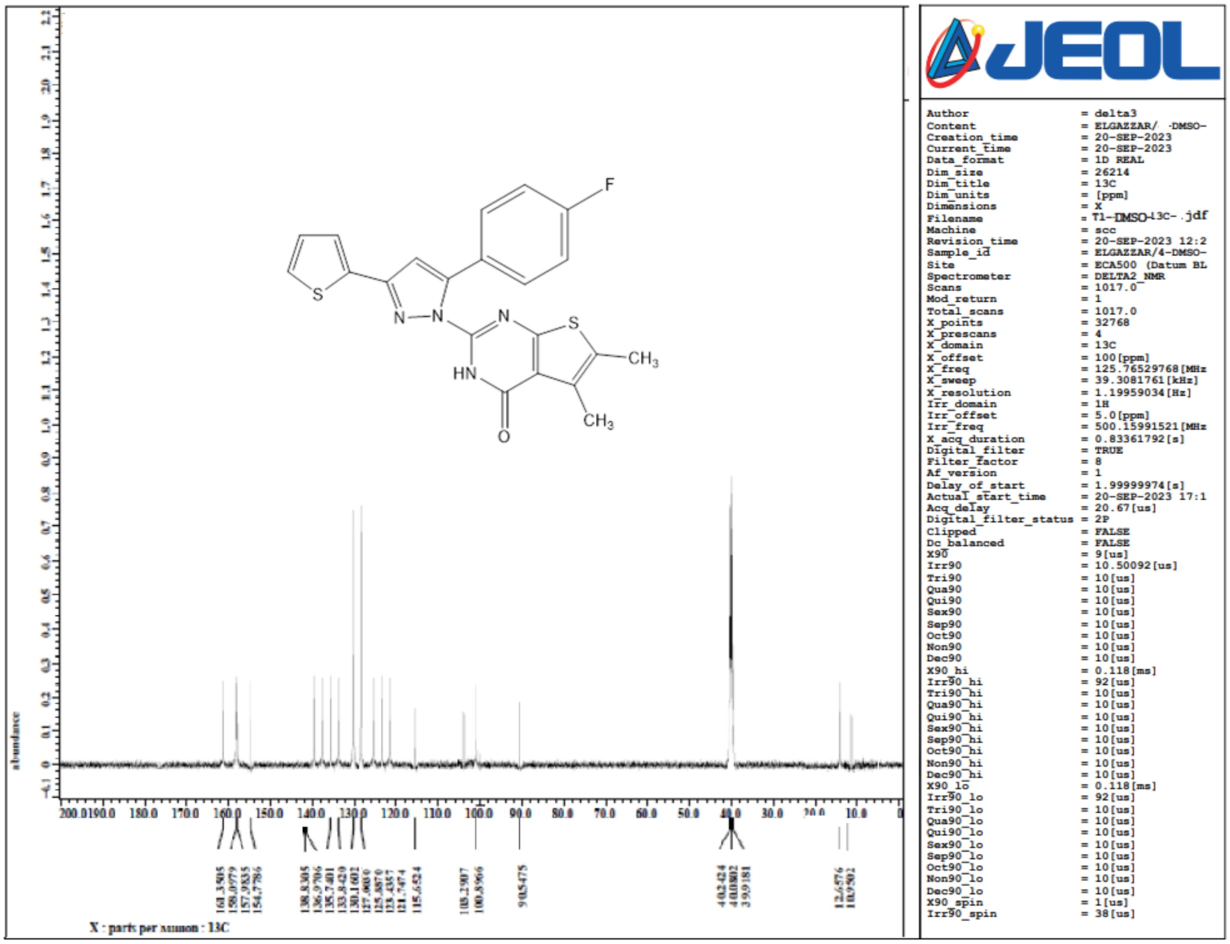

Fig.(8c). ^{13}C- NMR spectrum of compound **(5b)**

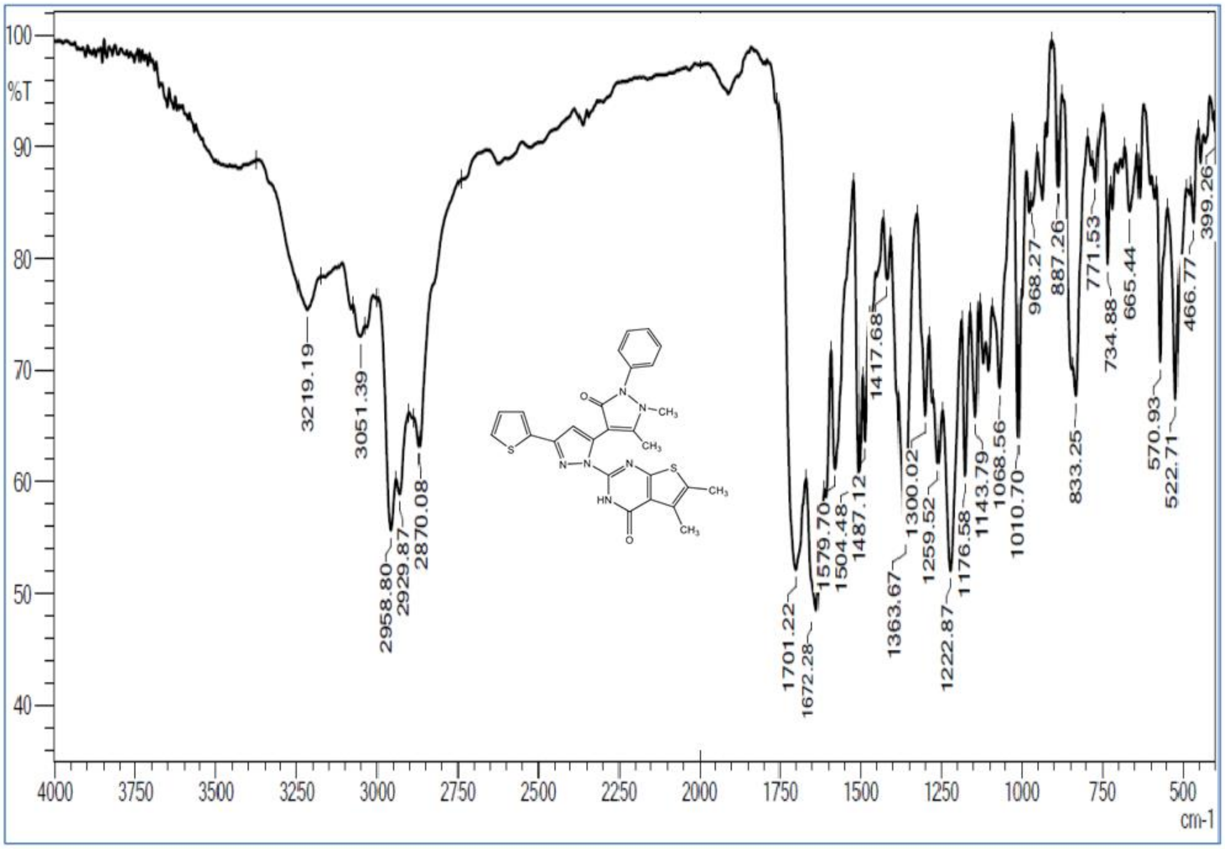

Fig.(9a). IR spectrum of compound **(5c)**

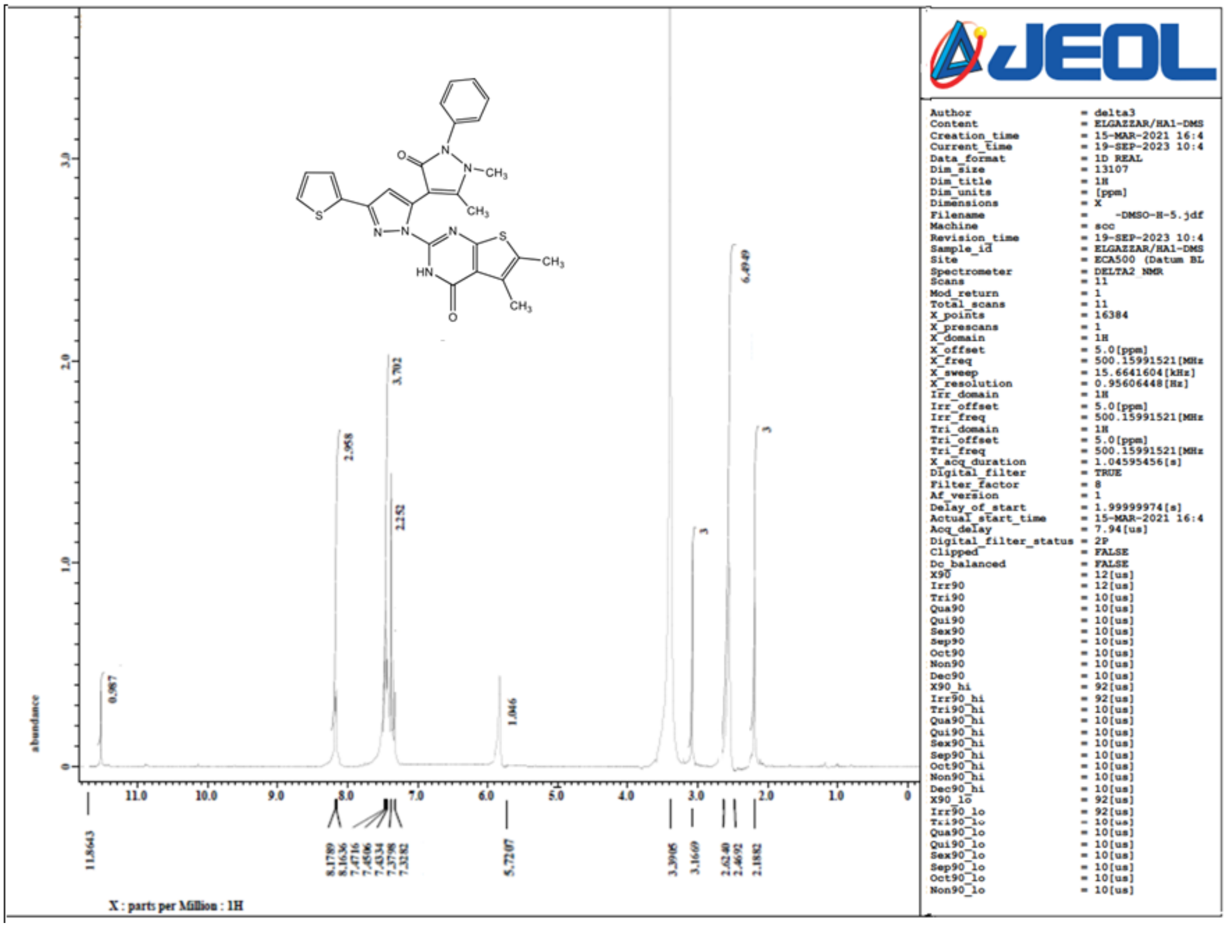

Fig.(9b). ^{1}H NMR spectrum of compound **(5c)**

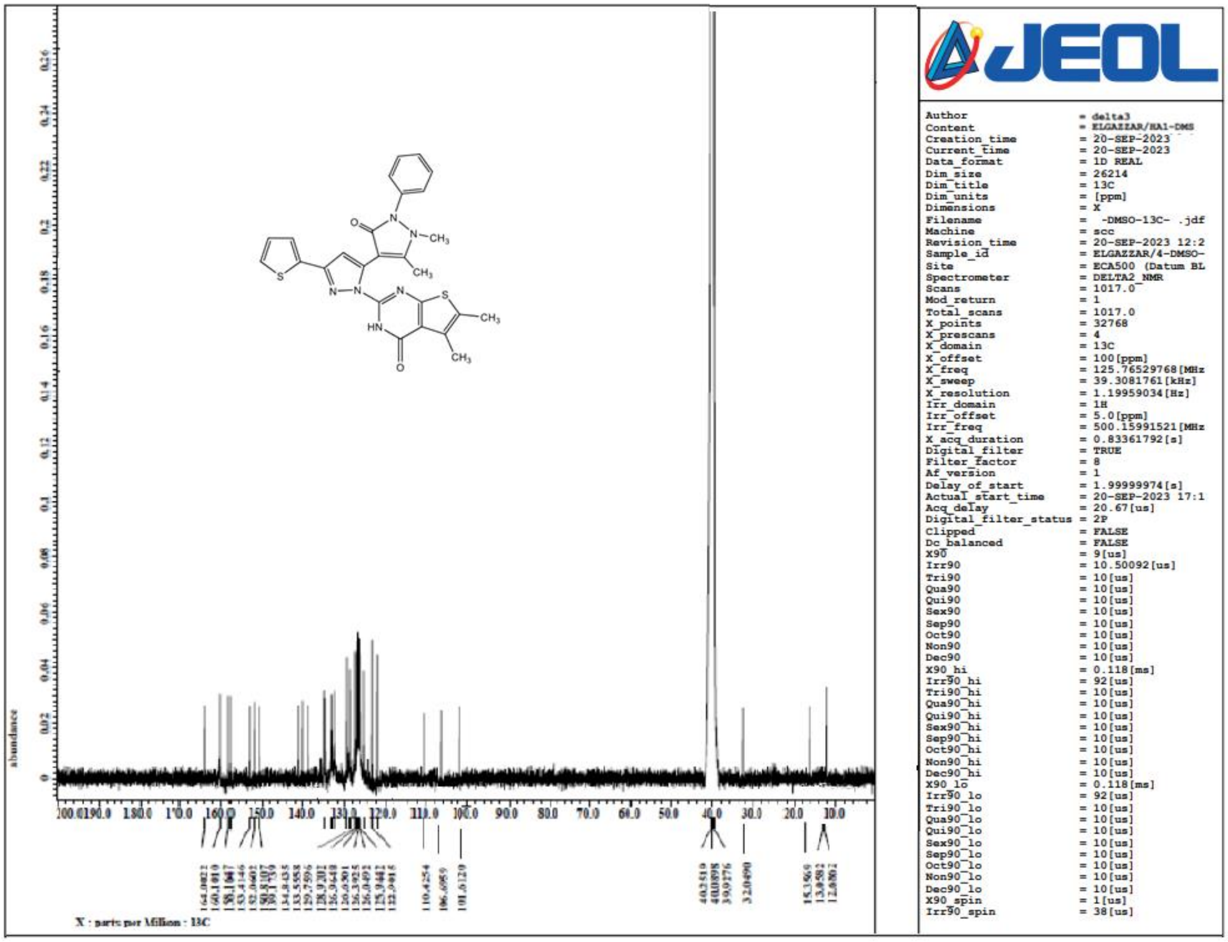

Fig.(9c). ^{13}C- NMR spectrum of compound **(5c)**

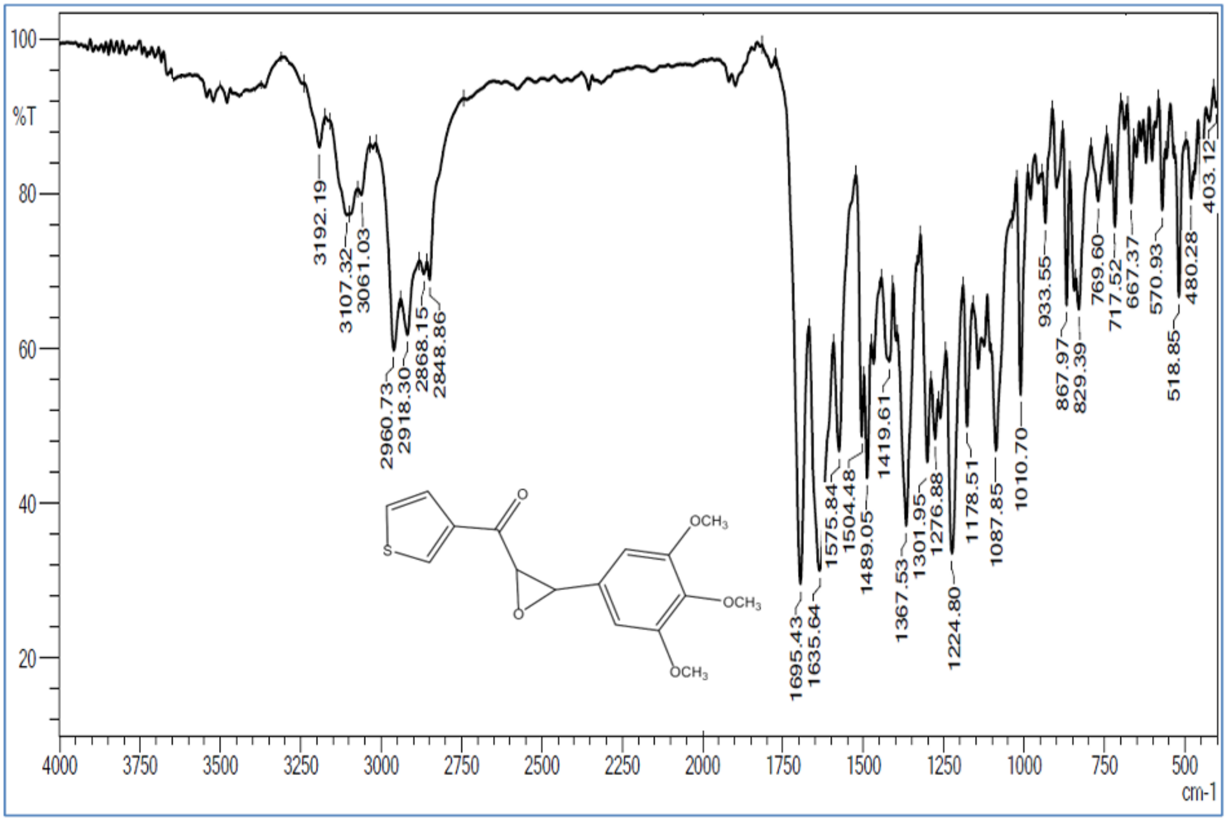

Fig.(10a). IR spectrum of compound **(6)**

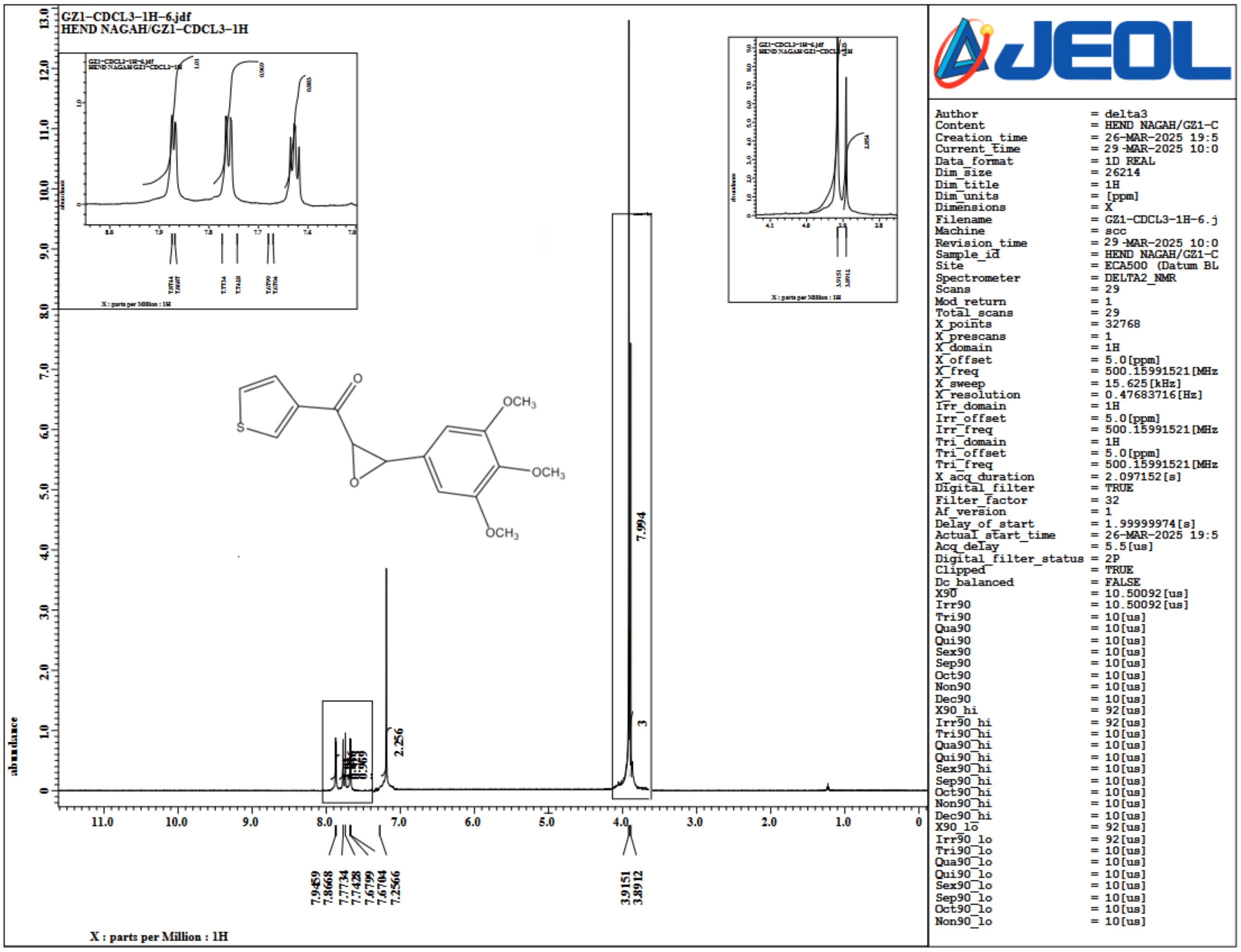

Fig.(10b). ^{1}H NMR spectrum of compound **(6)**

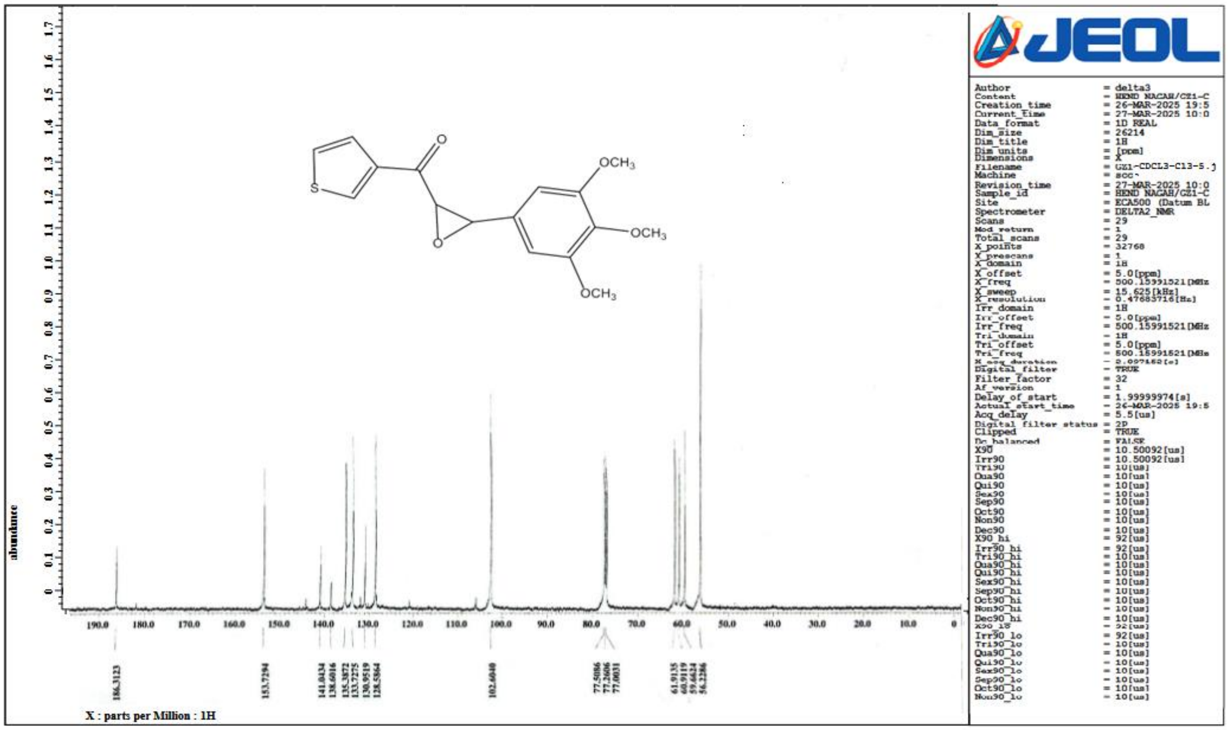

Fig.(10c). ^{13}C- NMR spectrum of compound **(6)**

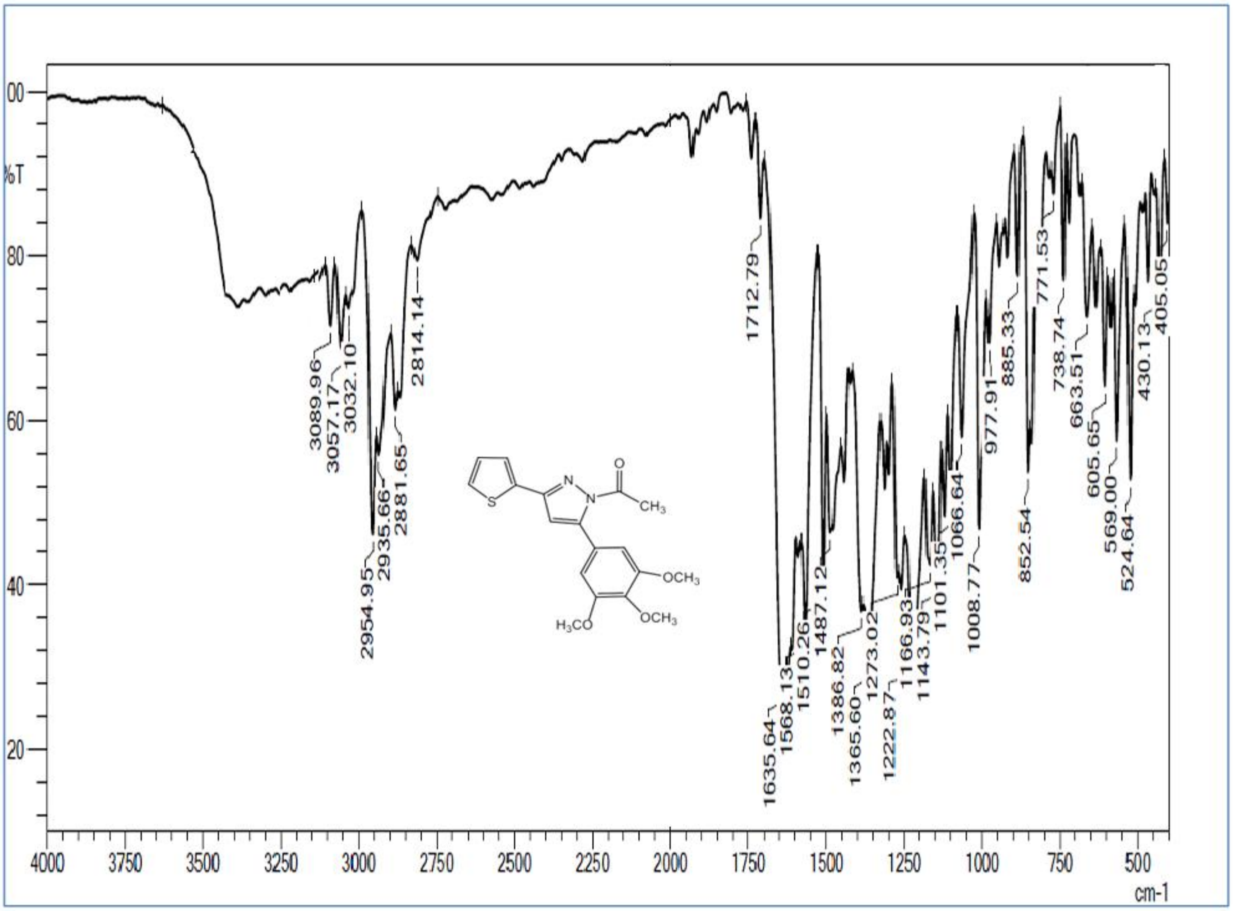

Fig.(11a). IR spectrum of compound **(7)**

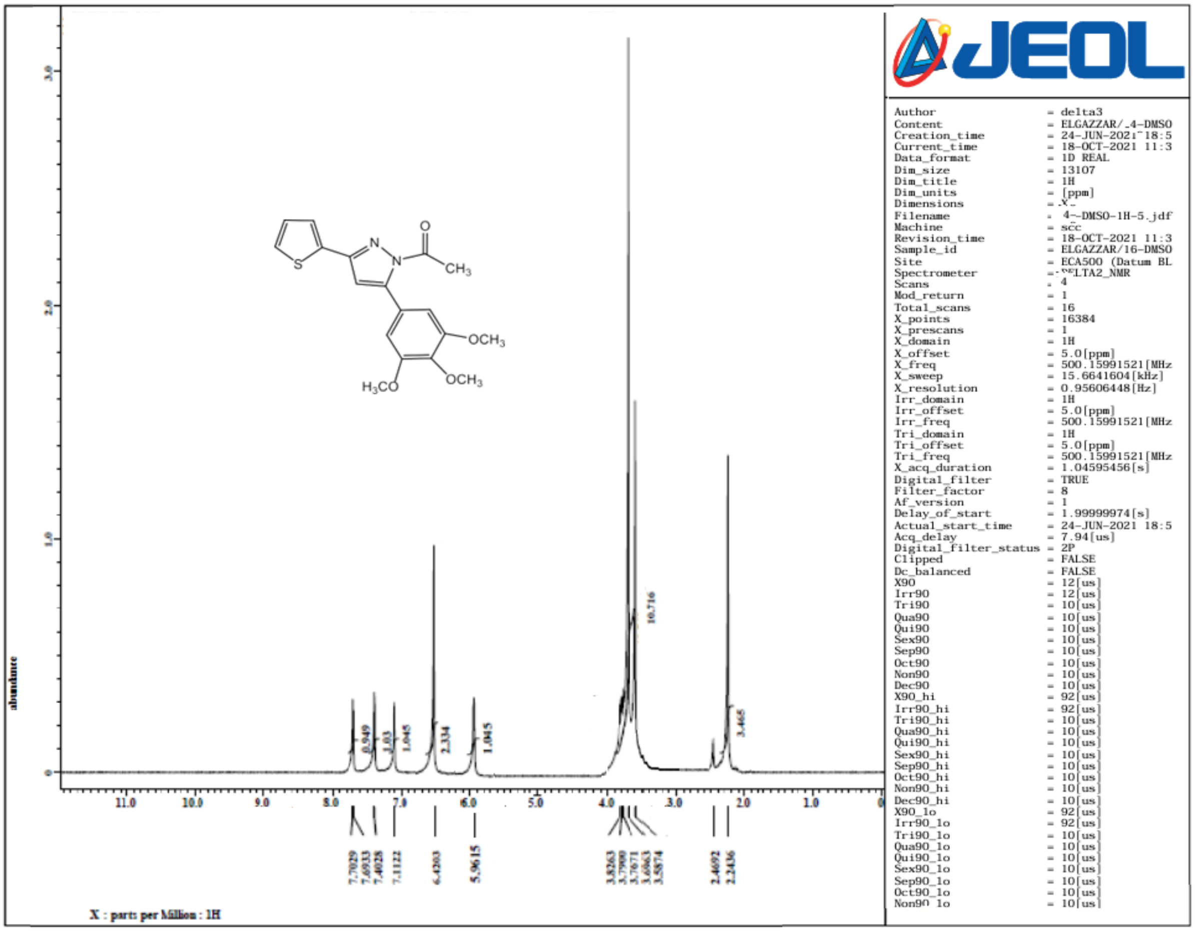

Fig.(11b). ^{1}H NMR spectrum of compound **(7)**

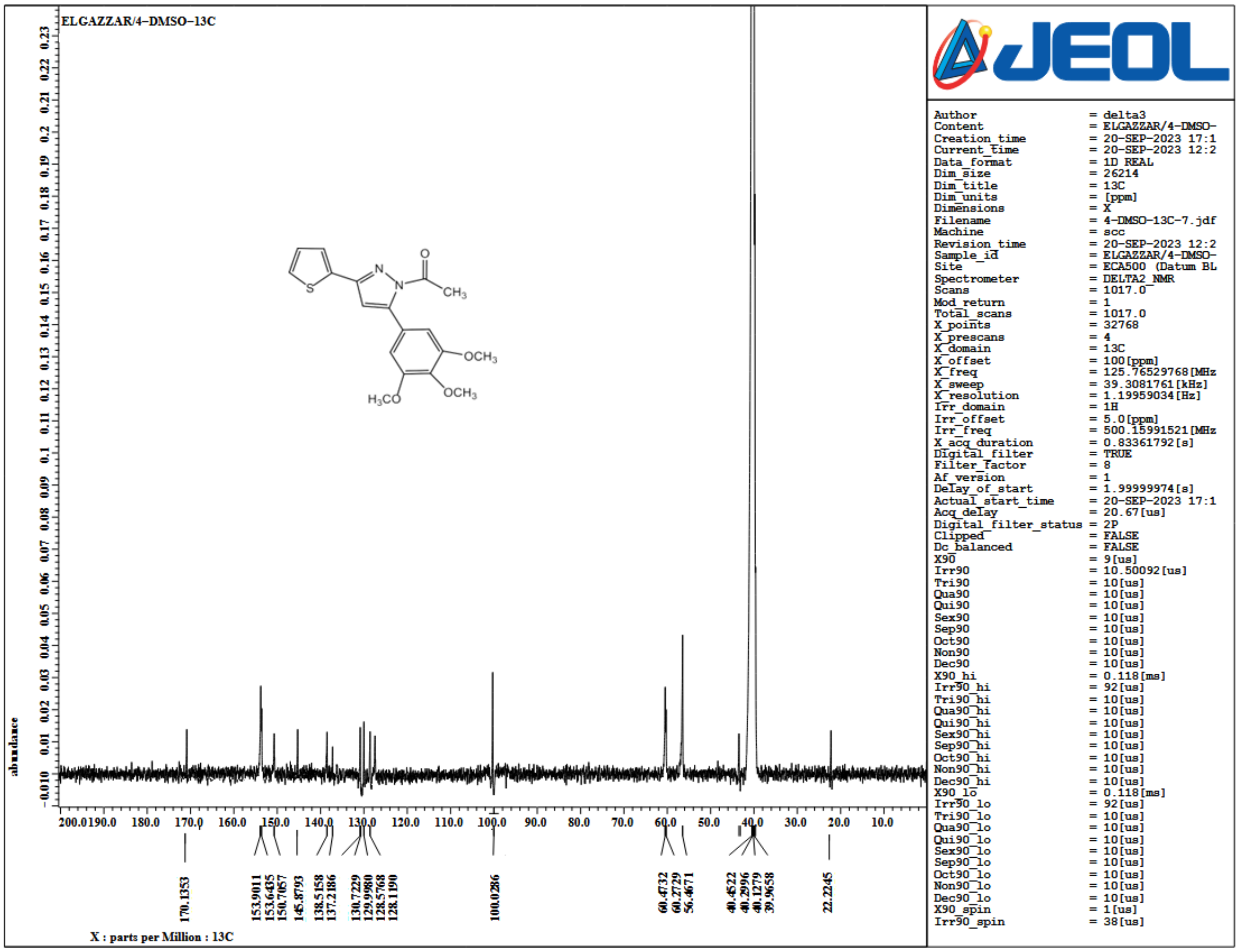

Fig.(11c). ^{13}C- NMR spectrum of compound **(7)**

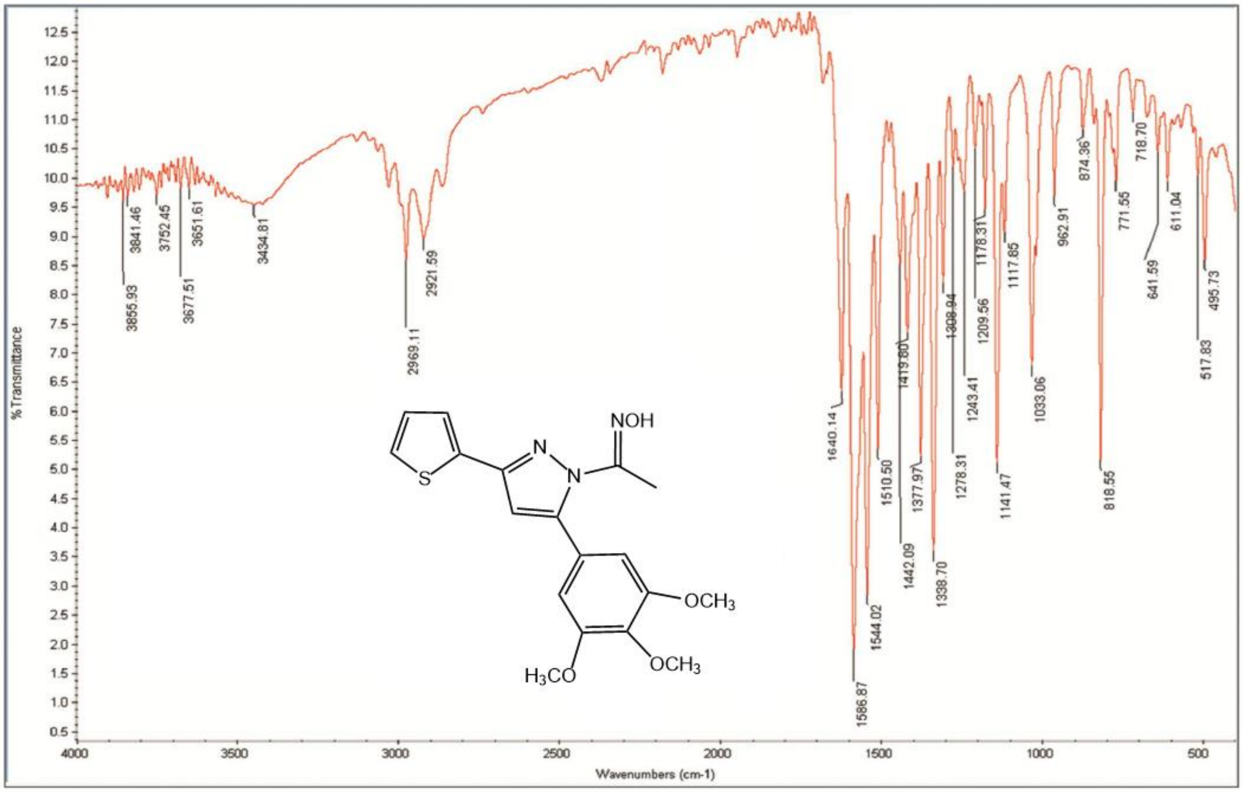

Fig.(12a). IR spectrum of compound **(8)**

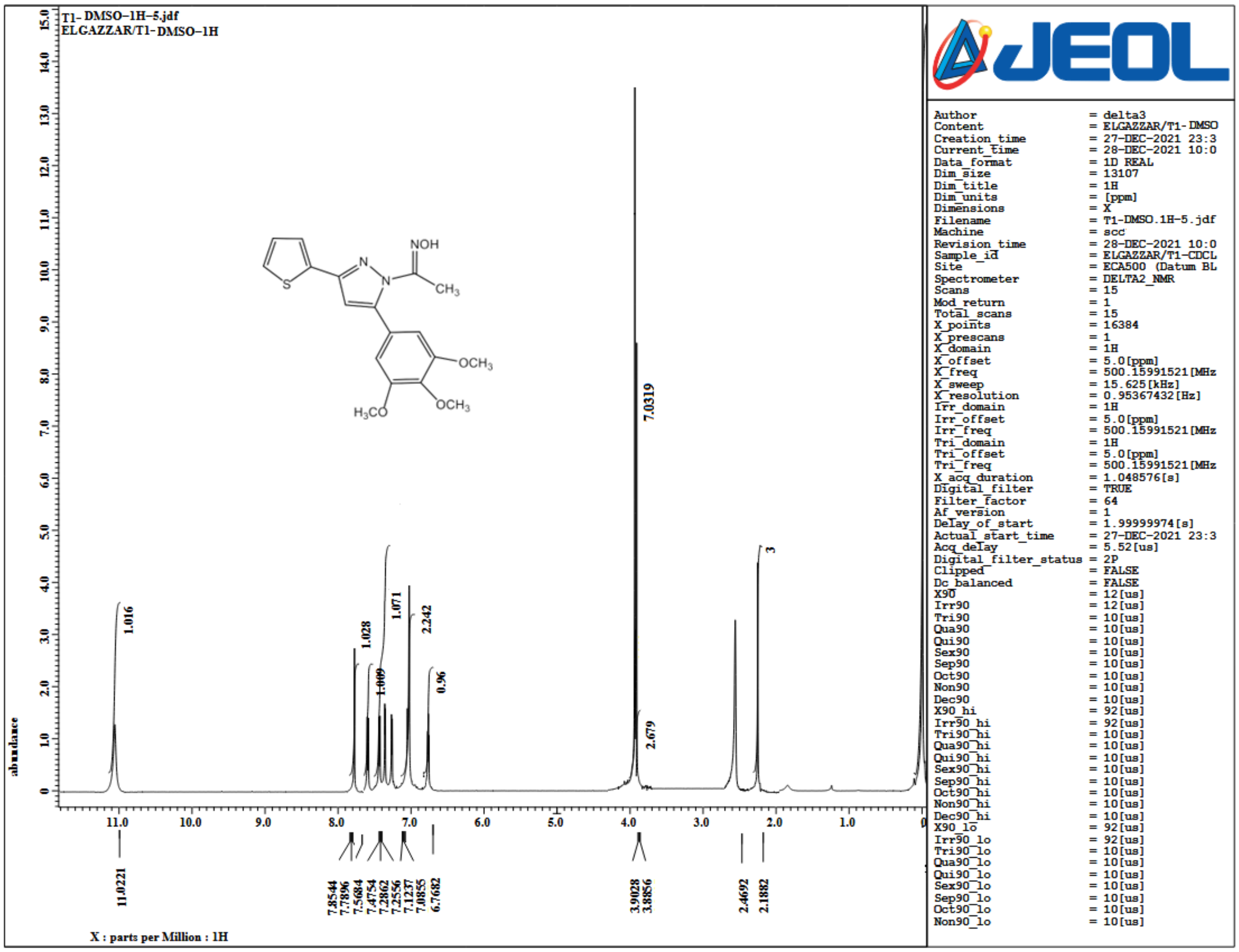

Fig.(12b). ^{1}H NMR spectrum of compound **(8)**

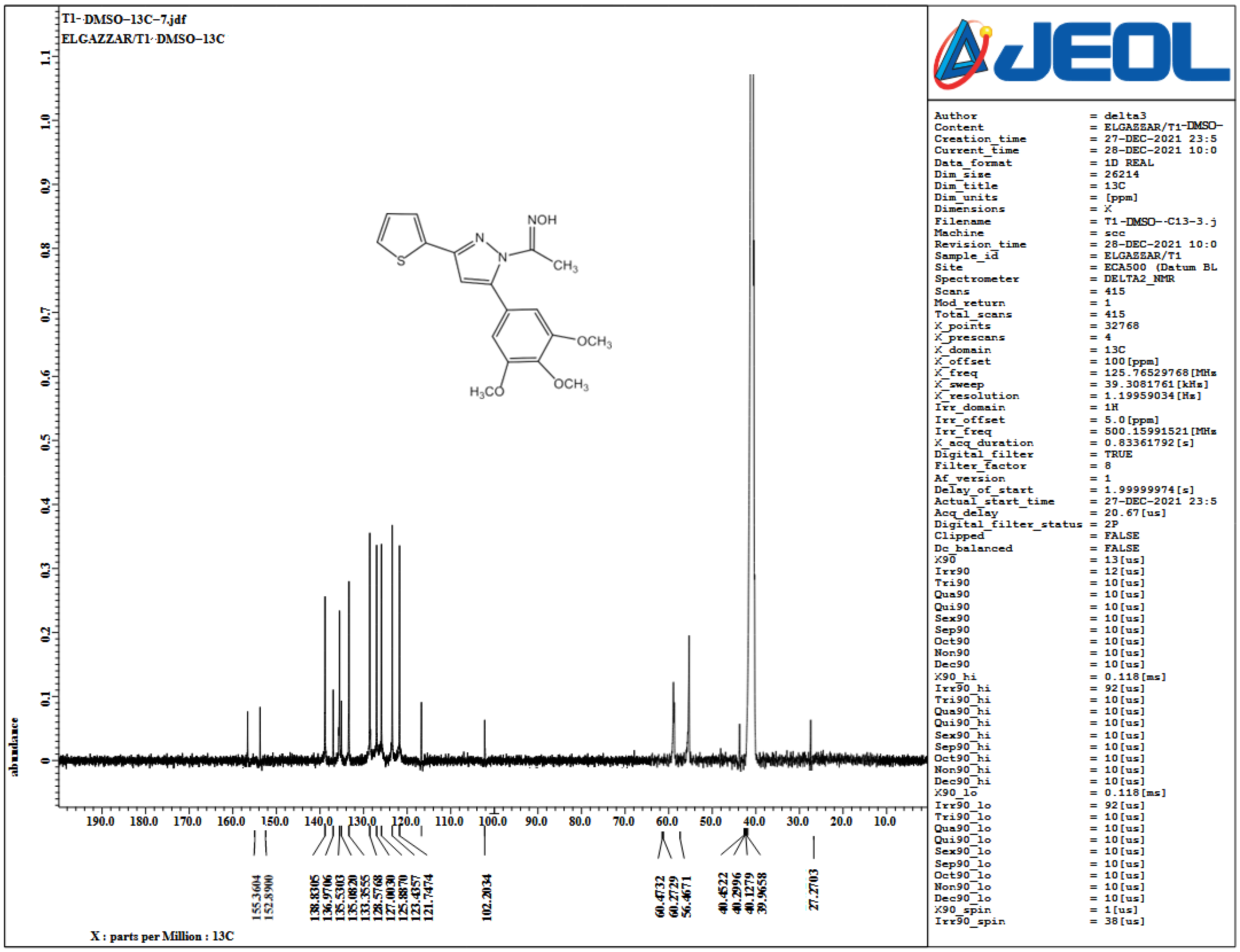

Fig.(12c). ^{13}C- NMR spectrum of compound **(8)**

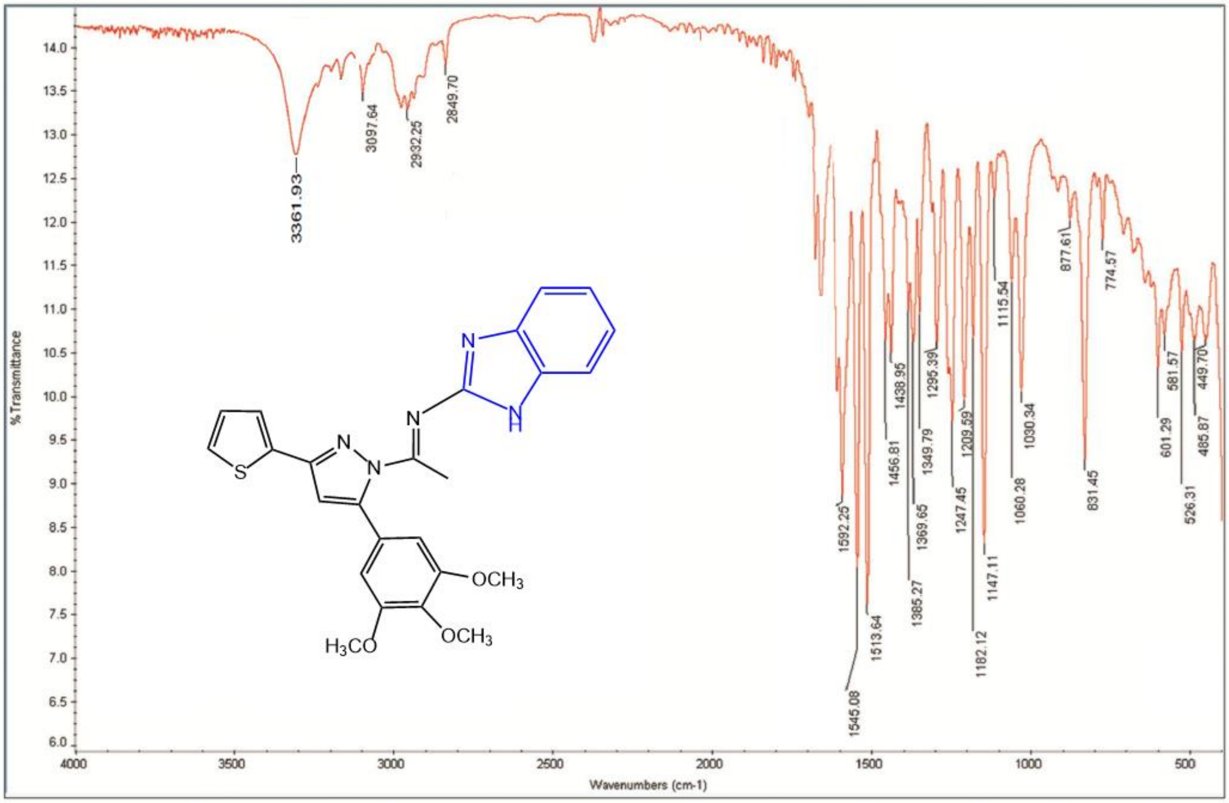

Fig.(13a). IR spectrum of compound **(9)**

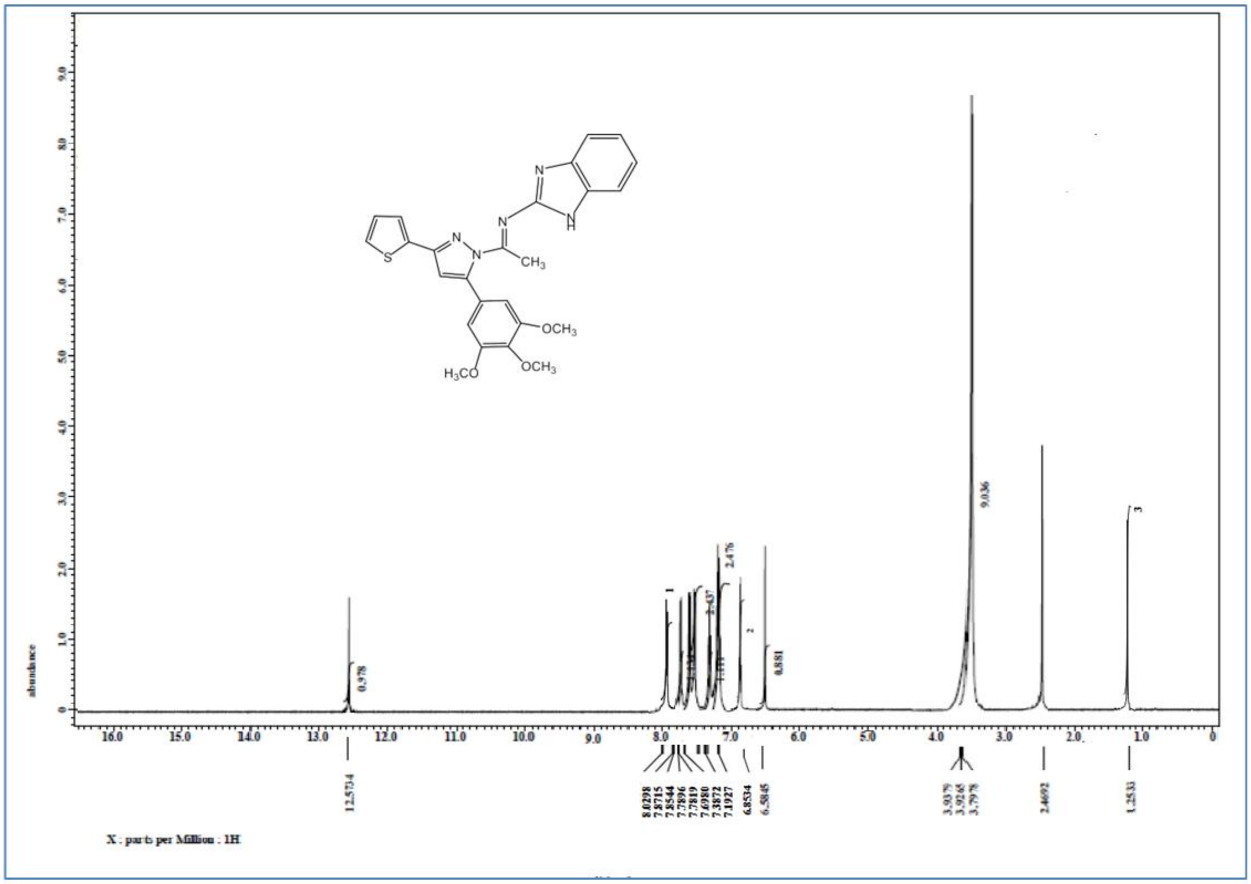

Fig.(13b). ^{1}H NMR spectrum of compound **(9)**

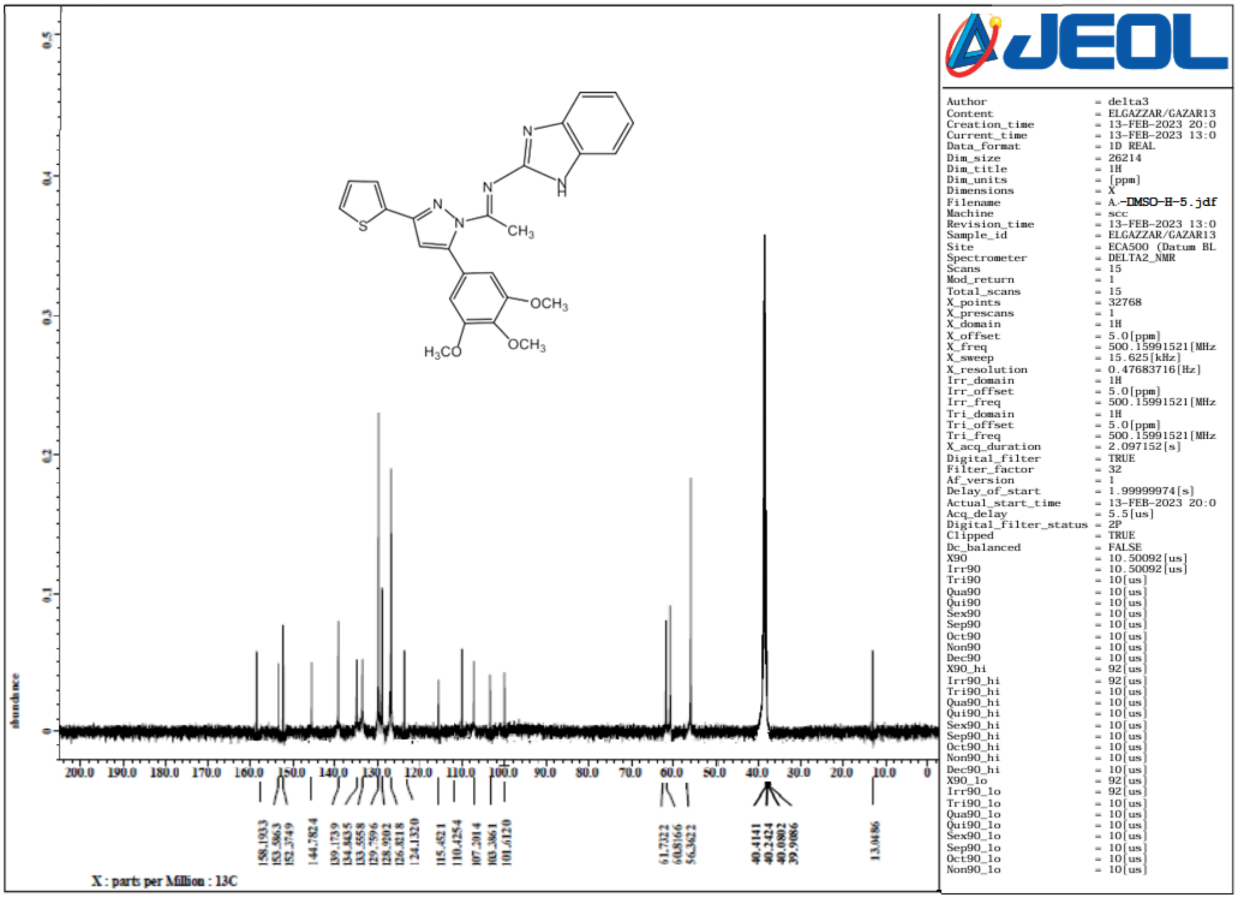

Fig.(13c). ^{13}C- NMR spectrum of compound **(9)**

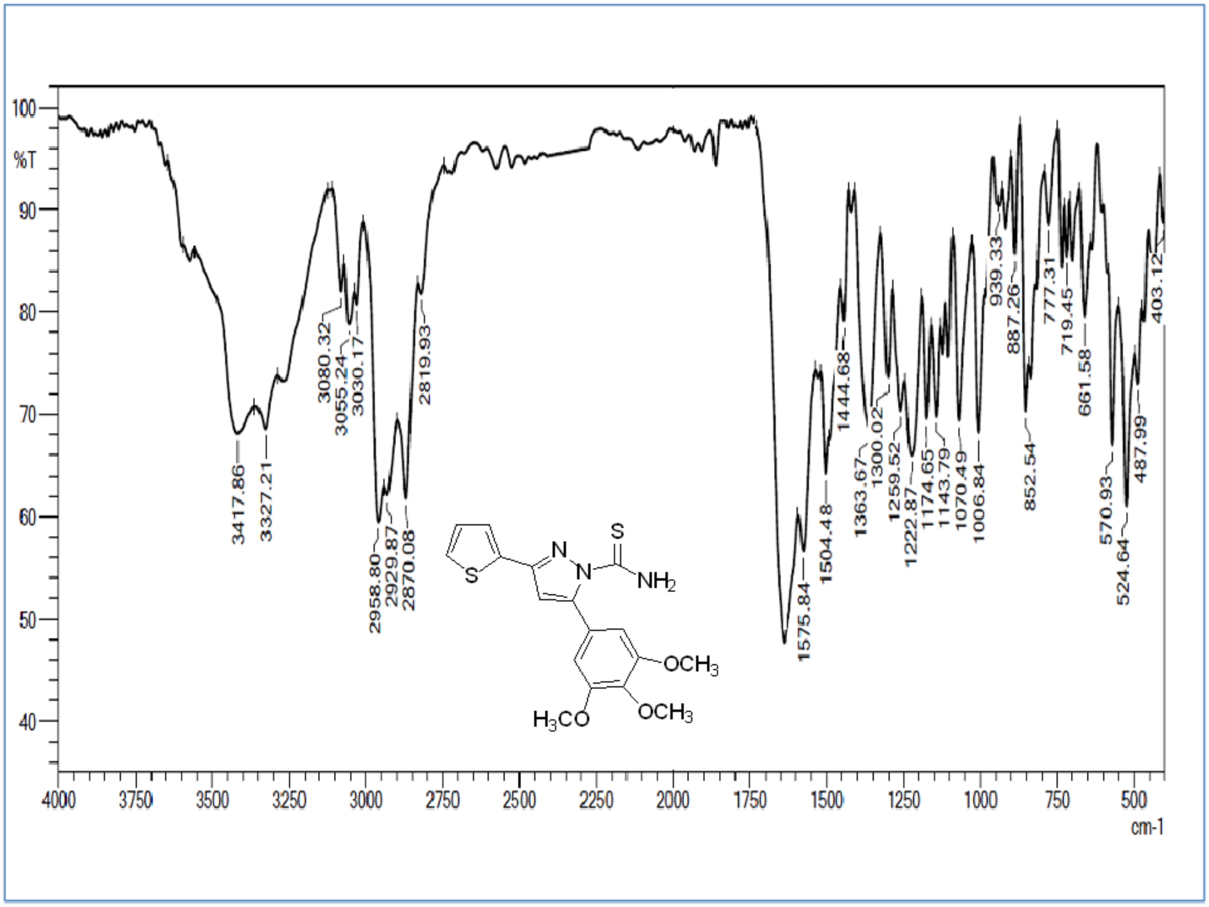

Fig.(14a). IR spectrum of compound **(10)**

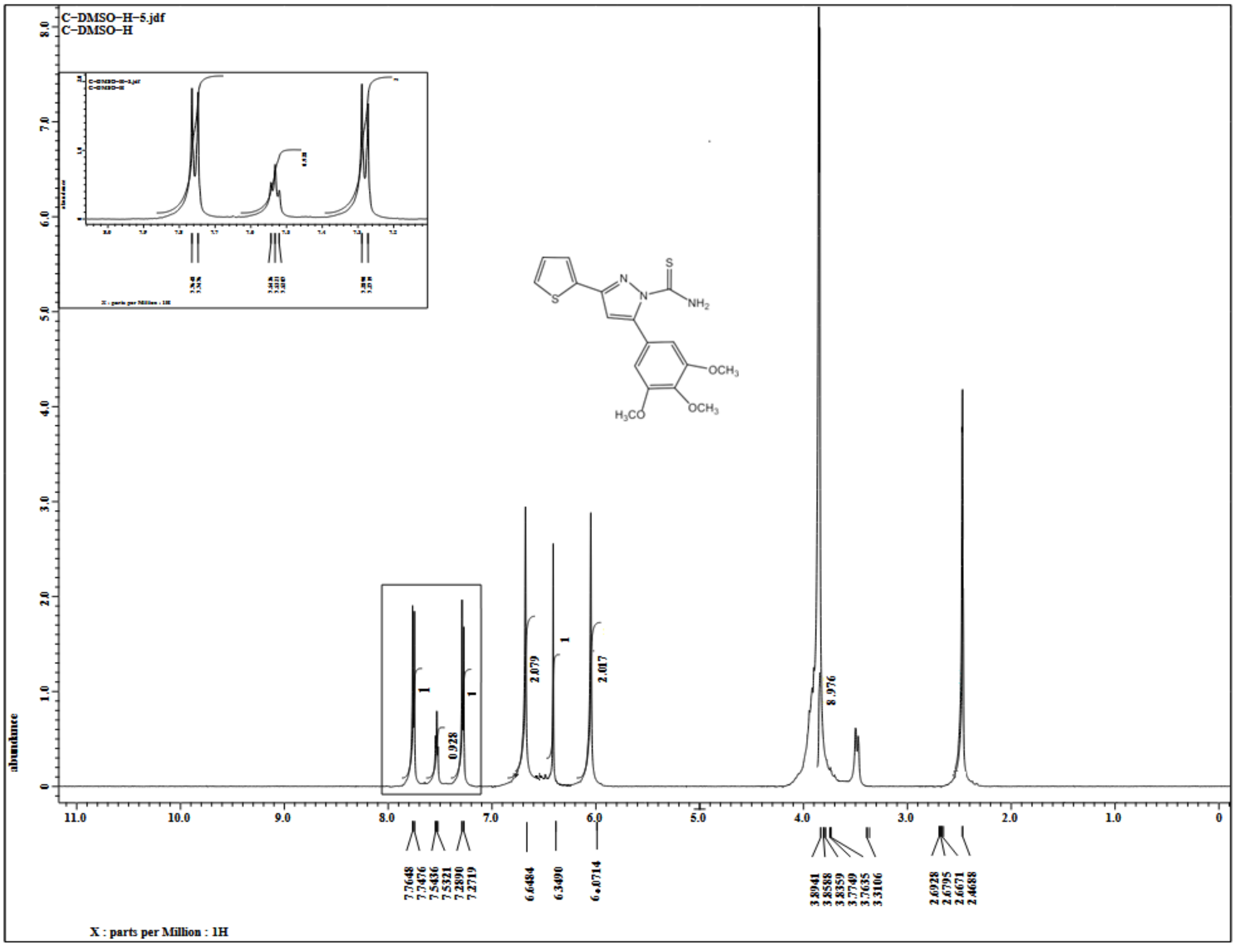

Fig.(14b). ^{1}H NMR spectrum of compound **(10)**

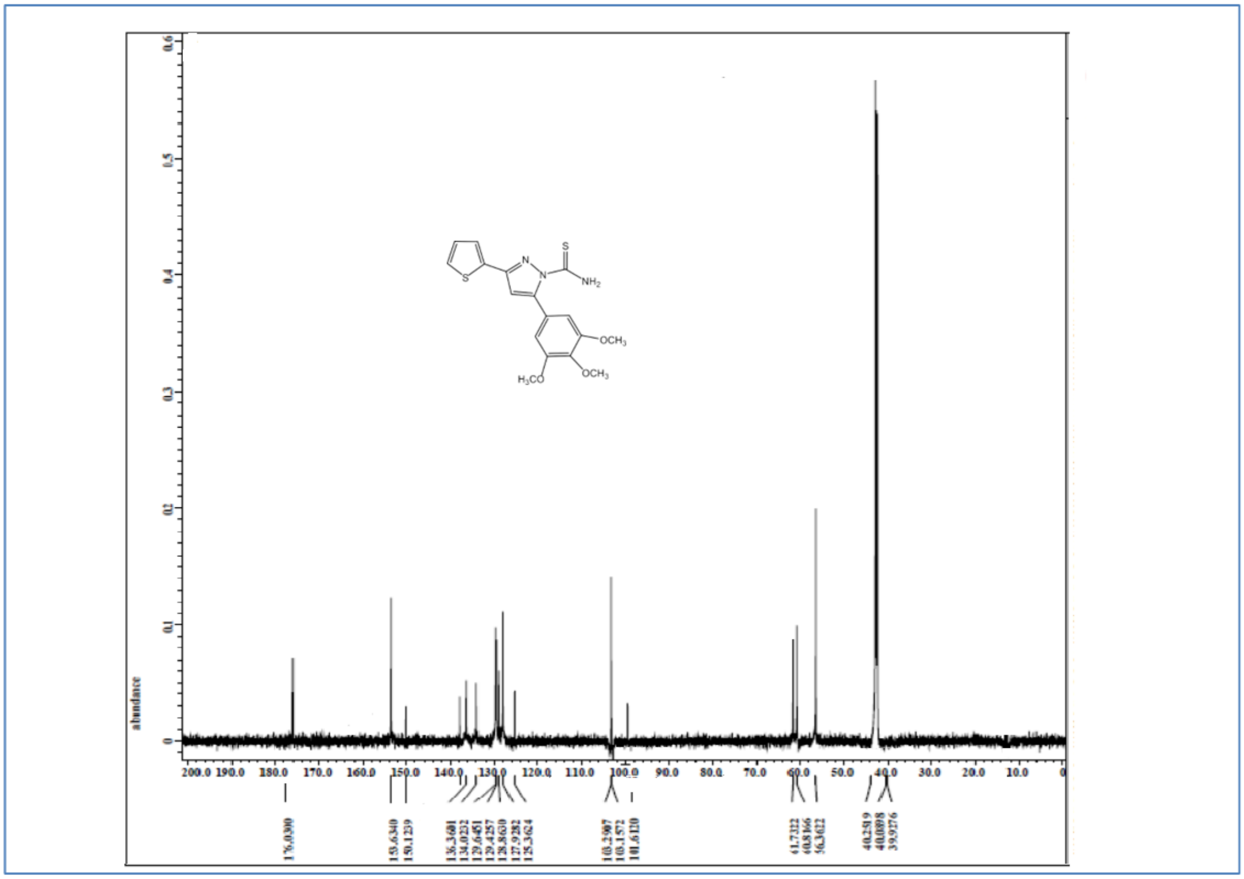

Fig.(14c). ^{13}C- NMR spectrum of compound **(10)**

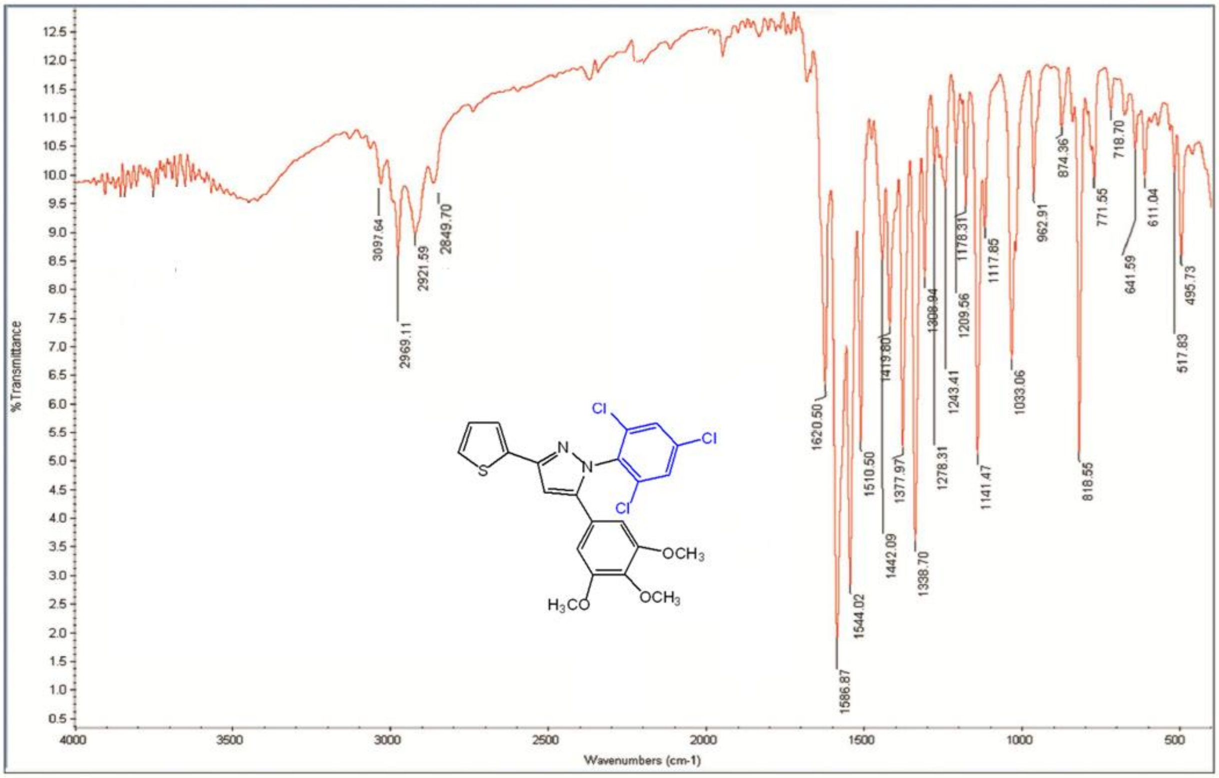

Fig.(15a). IR spectrum of compound **(11)**

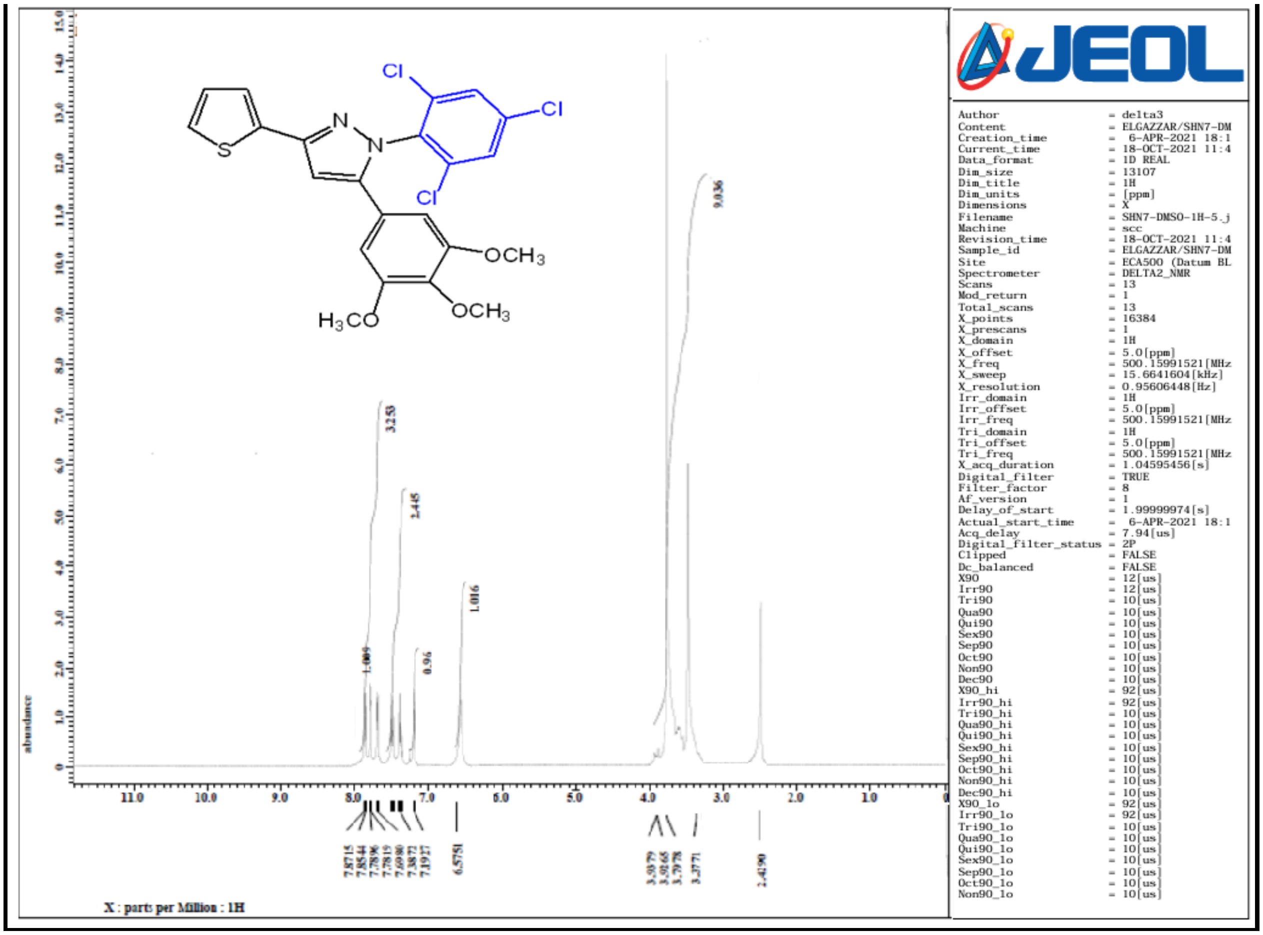

Fig.(15b). ^{1}H NMR spectrum of compound **(11)**

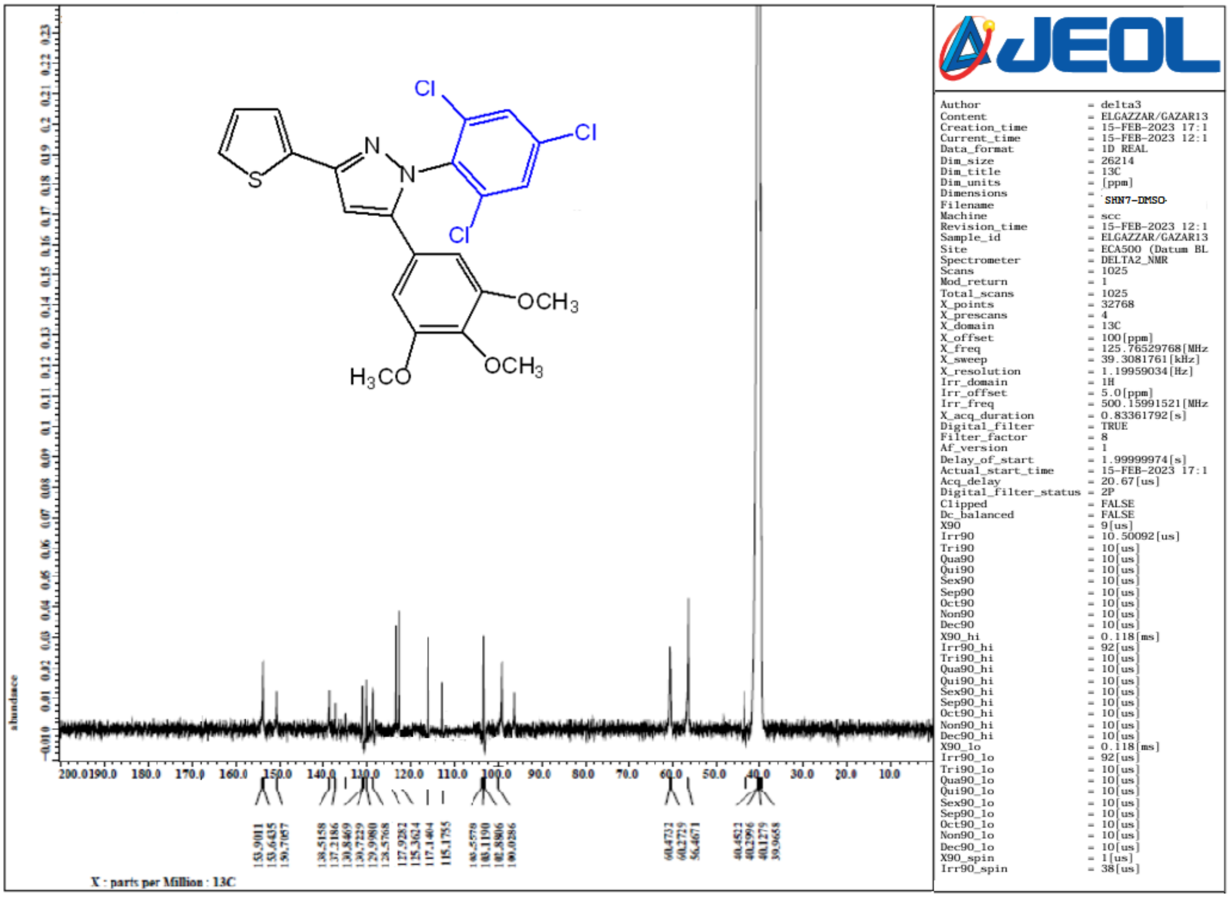

Fig.(15c). ^{13}C- NMR spectrum of compound **(11)**

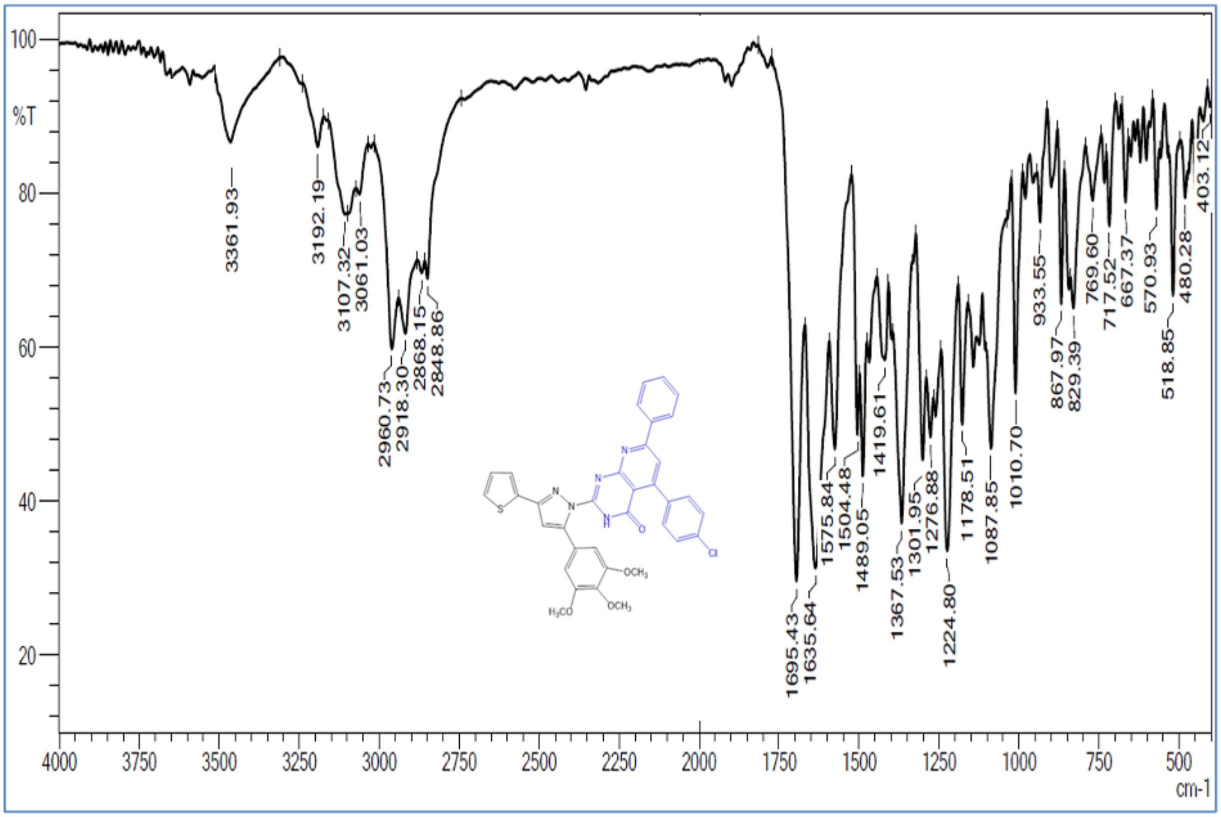

Fig.(16a). IR spectrum of compound **(12)**

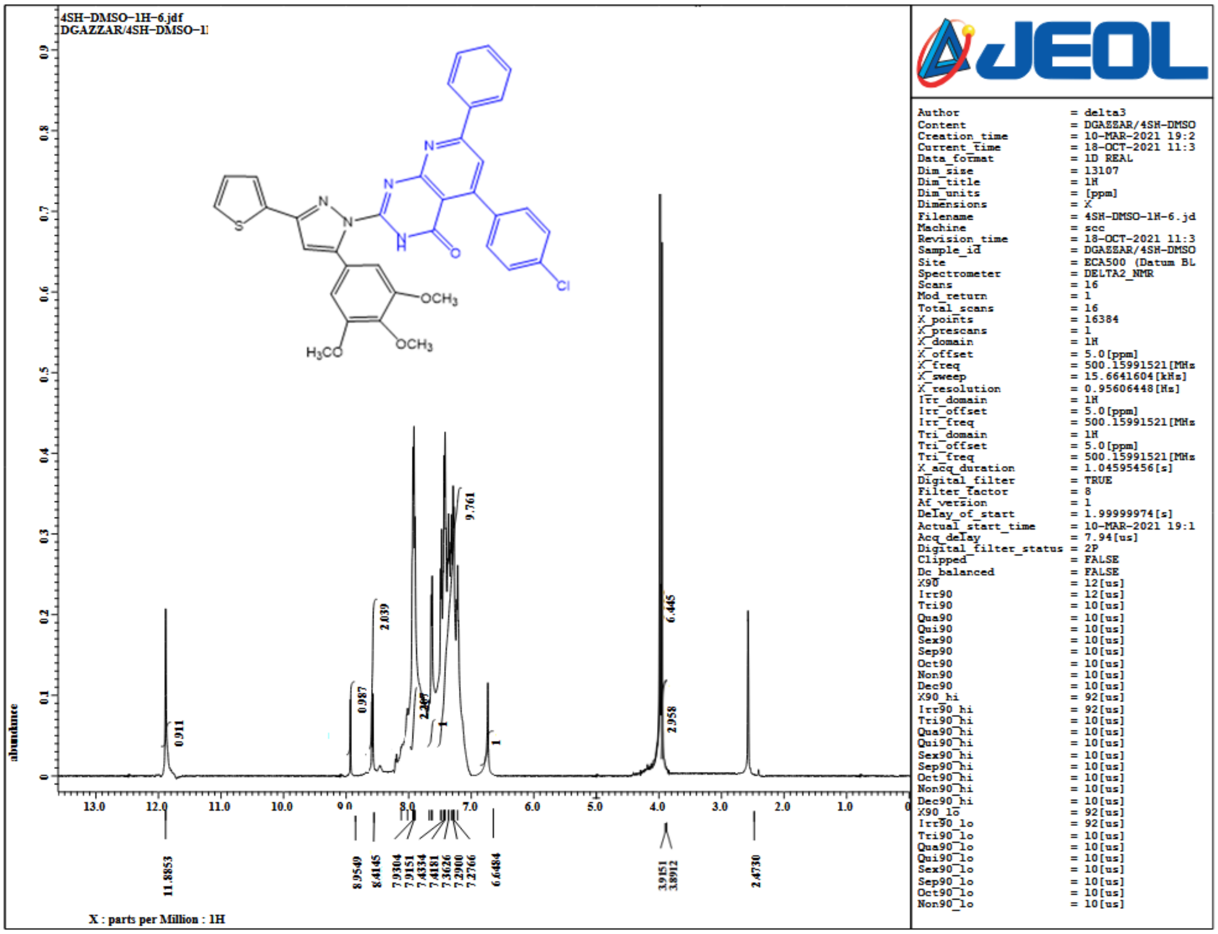

Fig.(16b). ^{1}H NMR spectrum of compound **(12)**

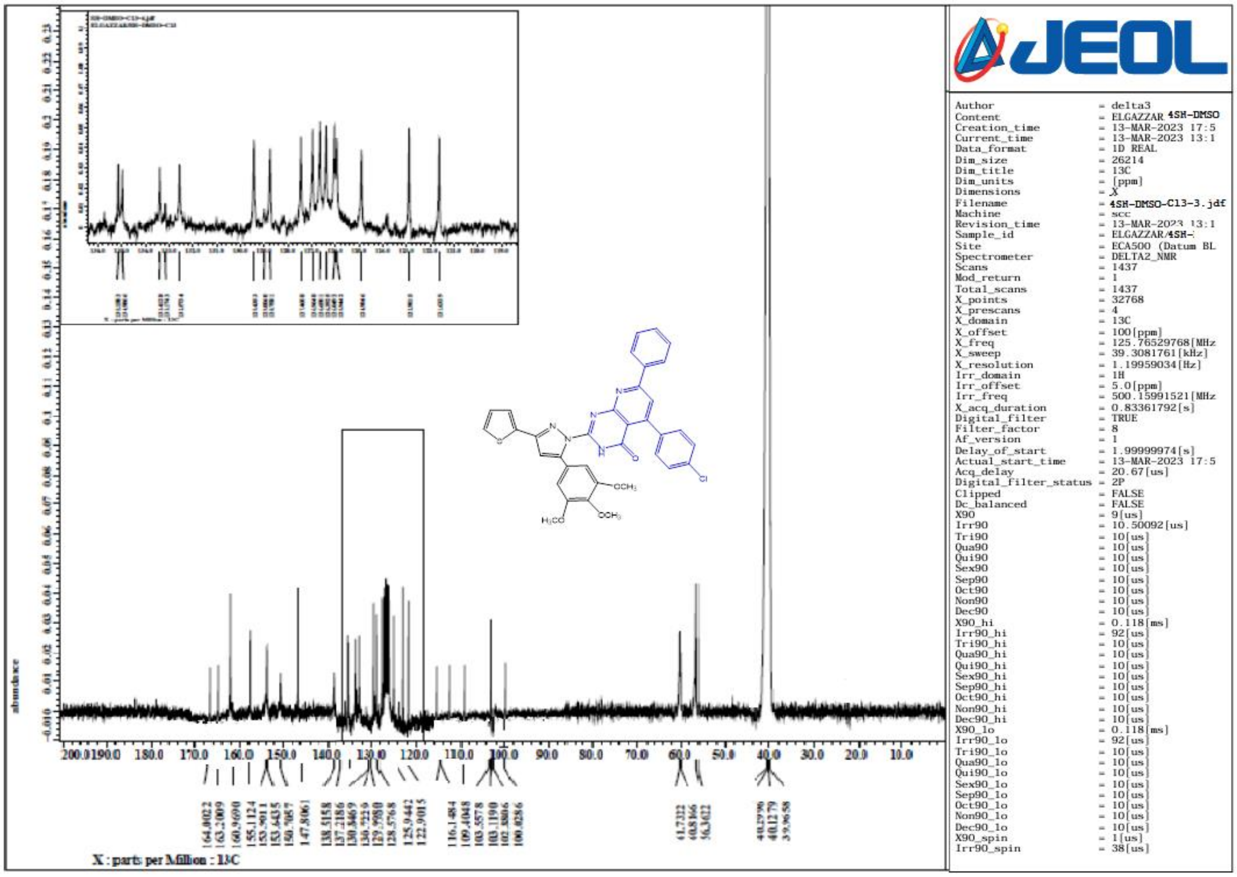

Fig.(16c). ^{13}C- NMR spectrum of compound **(12)**
