# Supplementary material for: Design, synthesis, characterization, pharmacological evaluation and in silico ADMET and molecular docking and dynamics simulations of a novel series of N-substituted pyrazole from chalcone derivatives
Source: Sci Rep. 2026 Mar 1;16:7931. doi: 10.1038/s41598-026-38237-9 (PMC12953870; doi:10.1038/s41598-026-38237-9)
Supplement: Supplementary file 2 — Supplementary Material 2 [file 41598_2026_38237_MOESM2_ESM.pdf]

Table S1: Binding Affinity of ligands with targets of activity

No	Ligands	Affinity (Kcal.mol-1)					
		B. subtilis (PDB.ID: 4URM)	***E.coli*** (PDB.ID: 7P2M)	***K. pneumoniae*** (PDB.ID: 2OV5)	***S. aureus*** (PDB.ID: 1AD4)	***S. pneumonia*** (PDB.ID: 8T8G)	***S. typhimurium*** (PDB.ID: 2SIL)
1	**10**	-6.80	-6.60	-6.30	-6.00	-6.00	-6.30
2	**11**	-6.70	-6.60	-6.30	-7.10	-6.70	-6.50
3	**12**	-7.40	-8.10	-7.30	-7.00	-6.60	-6.90
4	**4a**	-6.30	-6.10	-6.60	-5.80	-5.00	-6.70
5	**4b**	-6.30	-7.20	-6.60	-5.70	-5.70	-6.40
6	**4c**	-7.40	-7.70	-7.30	-7.20	-6.40	-7.50
7	**5a**	-6.50	-6.00	-6.70	-6.00	-6.10	-6.20
8	**5b**	-7.00	-7.80	-7.50	-5.90	-6.20	-6.90
9	**5c**	-8.10	-7.80	-7.80	-7.30	-6.70	-7.60
10	**6**	-5.70	-6.70	-7.40	-6.60	-5.70	-6.80
11	**7**	-6.30	-7.20	-7.10	-6.40	-5.10	-6.80
12	**8**	-5.60	-7.10	-6.30	-6.90	-5.90	-6.50
13	**9**	-6.20	-6.20	-6.90	-6.10	-6.00	-5.70
18	Levofloxacin	-7.10	-6.20	-6.70	-6.40	-5.30	-6.20

Table S2: Molecular interactions of ligands with amino acids of GyraseB of B. subtilis (PDB: ID 4URM)

No	Protein	Ligand	3D Structure	Hydrophilic Interactions		Hydrophobic Contacts		No. of H-Bonds	No. of Total Bonds	affinity kcal mol^{-1}
				Residue (H- Bond)	Length	Residue (Bond type)	Length			
1	GyraseB of B. subtilis (PDB: ID 4URM)	4c	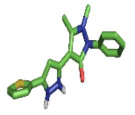	-	-	Pro87, (Pi-alkyl)<br>Ile102, (Pi-sigma)<br>Ala98, (Pi-alkyl)<br>Ile102, (Pi-alkyl)<br>Ile86, (Pi-alkyl)<br>Ile86, (Pi-sigma)	5.38<br>4.81<br>4.55<br>3.97<br>5.31<br>3.42	**0**	**6**	**-7.40**
2		5c	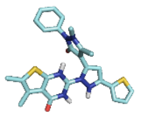	-	-	Ile102, (Pi-alkyl)<br>Ile86, (Pi-alkyl)<br>Asp57, (Pi-Cation)	5.08<br>4.75<br>3.46	**0**	**3**	**-8.10**
3		12	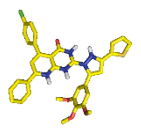	Gly109, (H- Bond)<br>Gly109, (H- Bond)	2.11<br>4.12	Lys111, (Pi-alkyl)<br>Leu103, (Pi-alkyl)<br>Ile102, (Pi-alkyl)<br>Ile86, (Pi-sigma)<br>Gly125, (Pi-cation)<br>Glu50, (Pi-Cation)<br>Asp57, (Pi-Cation)<br>Ser128, (Carbon H. bond)	4.11<br>5.22<br>4.84<br>3.46<br>3.93<br>3.12<br>4.12<br>3.02	**2**	**10**	**-7.40**
4		5b	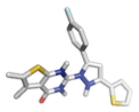	-	-	Ala98, (Pi-alkyl)<br>Pro87, (Pi-alkyl)<br>Leu103, (Pi-alkyl)<br>Ile86, (Pi-sigma)<br>Ile102, (Pi-sigma)<br>Ile86, (Pi-alkyl)<br>Asp81, (Carbon H. bond)	4.43<br>4.89<br>5.03<br>3.75<br>3.48<br>5.39<br>3.55	**0**	**7**	**-8.10**
5		Levofloxacin	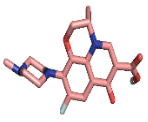	Ser128, (H- Bond)	2.65	Ile102, (Pi-alkyl)<br>Ile102, (Pi-alkyl)<br>Ile86, (Pi-alkyl)<br>Ile86, (Pi-sigma)<br>Asn54, (Pi-alkyl)<br>Asp81, (Carbon H. bond)	4.19<br>4.70<br>3.61<br>3.49<br>3.67<br>3.28	**1**	**7**	**-7.10**

Table S3: Molecular interactions of ligands with amino acids of DNA Gyrase of *E.coli* (PDB: ID 7P2M)

NO	Protein	Ligand	3D Structure	Hydrophilic Interactions		Hydrophobic Contacts		No. of H-Bonds	No. of Total Bonds	affinity kcal mol^{-1}
				Residue (H- Bond)	Length	Residue (Bond type)	Length			
1	DNA Gyrase of *E.coli* (PDB: ID 7P2M)	**4c**	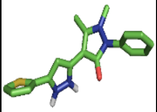	Asn46, (H- Bond)	2.43	Val120, (alkyl)<br>Ile78, (alkyl)<br>Ile94, (alkyl<br>Ile94, (Pi-sigma)<br>Glu50, (Pi-cation)<br>Glu50, (Carbon-H bond)	5.46<br>4.71<br>5.16<br>3.52<br>3.72<br>3.69	**1**	**7**	**-7.70**
2		**5c**	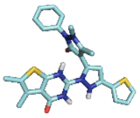	-	-	Val167, (alkyl)<br>Val120, (alkyl)<br>Val120, (alkyl<br>Ile78, (alkyl)<br>Pro79, (alkyl)<br>Ile94, (pi-Sigma)<br>Ile94, (pi-alkyl)<br>Thr165, (pi-Sigma)	5.05<br>5.41<br>5.40<br>4.82<br>3.63<br>3.31<br>4.62<br>3.70	**0**	**8**	**-7.80**
3		**12**	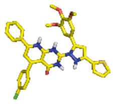	Asn46, (H- Bond)<br>Arg76, (H- Bond)	2.71<br>3.29	Leu98, (alkyl)<br>Val167, (alkyl)<br>Ile94, (alkyl<br>Val120, (alkyl)<br>Met95, (Sulfur)<br>Ile78, (Pi-sigma)	4.63<br>5.35<br>5.39<br>4.19<br>4.51<br>3.01	**2**	**8**	**-8.10**
4		**5b**	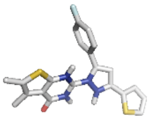	Asn46, (H- Bond)<br>Asn46, (H- Bond)	2.72<br>2.31	Thr165, (Pi-sigma)<br>Val167, (alkyl)<br>Ile78, (alkyl)<br>Ile94, (Pi-sigma)<br>Gly119, (Carbon-H bond)<br>Glu50, (pi-cation)<br>Arg76, (pi-cation)	3.65<br>5.40<br>4.76<br>3.92<br>3.79<br>4.38<br>4.06	**2**	**9**	**-7.80**
5		**Levofloxacin**	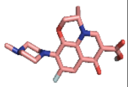	Asp73, (H- Bond)<br>Gly77, (H- Bond)	2.48<br>2.93	Ile94, (alkyl)<br>Ile94, (alkyl)<br>Ile78, (alkyl)<br>Ile78, (alkyl<br>Glu50, (Carbon-H bond)<br>Asn46, (Carbon-H bond)<br>Asn46, (halogen)	5.01<br>5.42<br>4.74<br>5.29<br>3.25<br>3.66<br>3.09	**2**	**9**	**-6.20**

Table S4: Molecular interactions of ligands with amino acids of KPC-2 carbapenemase of *K. pneumoniae* (PDB: ID 2OV5)

No	Protein	Ligand	3D Structure	Hydrophilic Interactions		Hydrophobic Contacts		No. of H-Bonds	No. of Total Bonds	affinity kcal mol^{-1}
				Residue (H- Bond)	Length	Residue (Bond type)	Length			
1	KPC-2 carbapenemase of *K. pneumoniae* (PDB: ID 2OV5)	**4c**	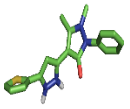	-	-	Trp105, (Pi-Pi T shaped)<br>Trp105, (Pi-alkyl)<br>Asn132, (Carbon-H bond)<br>Leu167, (alkyl)	4.63<br>4.43<br>3.20<br>5.05	**0**	**4**	**-7.30**
2		**5c**	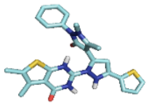	-	-	Glu276, (Carbon-H bond)	2.85	**0**	**1**	**-7.80**
3		**12**	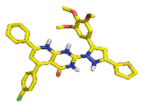	Thr215, (H- Bond)	2.86	Glu276, (Carbon-H bond)<br>Glu276, (Pi-cation)<br>Arg220, (Pi-cation)	5.44<br>4.80<br>4.85	**6**	**10**	**-7.30**
4		**5b**	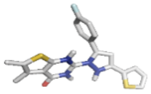	Ser130, (H- Bond)	2.90	Trp105, (Pi-Pi T shaped)<br>Trp105, (Sulfur)<br>Glu276, (Halogen)	4.02<br>4.01<br>2.51	**1**	**4**	**-7.50**
5		**Levofloxacin**	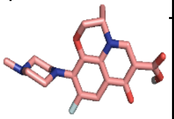	Asn170, (H- Bond)	2.18<br>2.56<br>2.98	Trp105, (Pi-Pi T shaped)<br>Glu276, (Carbon-H bond)<br>Glu276, (Carbon-H bond)	4.73<br>3.74<br>3.55	**3**	**6**	**-6.70**

Table S5: Molecular interactions with amino acids of dihydropteroate synthase of *S. aureus* (PDB: ID 1AD4)

No	Protein	Ligand	3D Structure	Hydrophilic Interactions		Hydrophobic Contacts		No. of H-Bonds	No. of Total Bonds	affinity kcal mol^{-1}
				Residue (H- Bond)	Length	Residue (Bond type)	Length			
1	Dihydropteroate synthase of *S. aureus*	4c	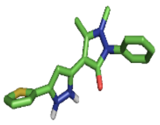	Lys203 (H- Bond)<br>Asp84 (H- Bond)	2.46<br>2.47	Phe172, (Pi-Pi shaped)<br>Phe172, (Pi-Pi shaped)<br>Arg239, (Pi-cation)<br>Arg239, (Pi-cation)<br>His241, (Pi-cation)<br>Ala199, (Pi-alkyl)<br>Met128, (Pi-alkyl)<br>Arg202, (Pi-alkyl)	5.63<br>5.75<br>3.69<br>4.60<br>3.87<br>4.82<br>4.50<br>5.04	**2**	**10**	**-7.20**
2		5c	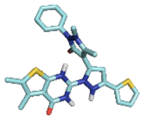	His55, (H- Bond)<br>Arg239, (H- Bond)<br>Asn11, (H- Bond)	2.78<br>2.99<br>2.40	Phe172, (Pi-Pi shaped)<br>Phe172, (Sulfur)<br>His55, (Pi-alkyl)<br>Lys203, (Pi-alkyl)<br>Met128, (Sulfur)<br>Lys203, (Pi-alkyl)	5.04<br>5.81<br>4.86<br>4.87<br>4.76<br>5.58	**3**	**9**	**-7.30**
3		12	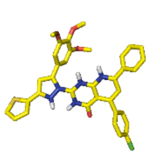	-	-	Met128, (Pi-alkyl)<br>Ala199, (Pi-alkyl)<br>Arg204, (Pi-alkyl)<br>His55, (Pi-alkyl)<br>Pro216, (Pi-alkyl)<br>Asp84, (Pi-cation)<br>Arg52, (Pi-cation)<br>Lys203, (CH-bond)	3.74<br>4.16<br>4.21<br>4.42<br>4.24<br>4.53<br>4.90<br>2.67	0	**8**	**-7.00**
4		11	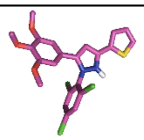	Asn11 (H- Bond)	2.82	Pro216, (Pi-alkyl)<br>His241, (Pi-alkyl)<br>Phe172, (Sulfur)<br>His241, (Pi-cation)<br>Arg239, (Pi- cation)<br>Arg202, (CH-bond)	4.40<br>4.88<br>5.75<br>3.61<br>3.51<br>3.33	**1**	**7**	**-7.10**
5		Levofloxacin	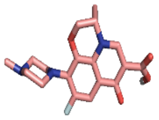	Lys203, (H- Bond)<br>Gln105, (H- Bond)	2.50<br>2.56	His241, (Pi-alkyl)<br>Lys203, (Pi-alkyl)<br>Arg239, (Pi-cation)	4.92<br>4.54<br>4.05	**2**	**5**	**-6.40**

Table S6: Molecular interactions of ligands with Streptococcus pneumonia Sortase A (spySrtA) (8T8G)

NO	Protein	Ligand	3D Structure	Hydrophilic Interactions		Hydrophobic Contacts		No. of H-Bonds	No. of Total Bonds	affinity kcal mol^{-1}
				Residue (H- Bond)	Length	Residue (Bond type)	Length			
1	***S. pneumonia* Sortase A (spySrtA) (8T8G)**	**4c**	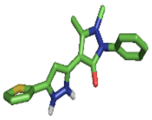	-	-	Arg216, (Pi-cation)<br>Arg216, (Pi-alkyl)<br>Ala208, (Pi-alkyl)<br>Val206, (Pi-alkyl)<br>Met125, (Pi-alkyl)<br>Ile194, (Pi-alkyl)<br>Val191, (Pi-alkyl)	3.95<br>4.18<br>4.23<br>4.33<br>5.32<br>5.17<br>5.33	**0**	**7**	**-6.40**
2		**5c**	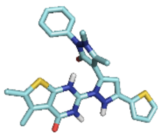	-	-	Val193, (Pi-alkyl)<br>Val206, (Pi-alkyl)<br>Ala208, (Pi-alkyl)<br>Ala213, (Pi-alkyl)<br>Leu113, (Pi-alkyl)<br>Leu118, (Pi-alkyl)<br>Leu113, (Pi-alkyl)<br>Arg216, (Pi-cation)	4.12<br>5.05<br>4.09<br>5.41<br>4.76<br>4.55<br>3.59<br>4.66	**0**	**8**	**-6.70**
3		**12**	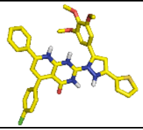	-	-	Ala140, (Pi-alkyl)<br>Leu118, (Pi-alkyl)<br>Leu118, (Pi-alkyl)<br>Val191, (Pi-alkyl)	5.21<br>5.12<br>5.47<br>5.06	**0**	**4**	**-6.60**
		**11**	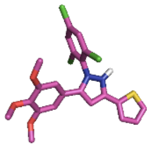	-	-	Leu118, (Pi-alkyl)<br>Ala140, (Pi-alkyl)<br>His142, (Pi-alkyl)<br>Ile218, (Pi-alkyl)<br>Val186, (Pi-alkyl)<br>Val193, (Pi-alkyl)<br>Arg190, (Pi-alkyl)<br>Val191, (Pi-alkyl)<br>Leu113, (Carbon H bond)	4.84<br>4.80<br>4.04<br>5.17<br>4.18<br>4.44<br>4.75<br>5.47<br>3.06	**0**	**9**	**-6.70**
4		**Levofloxacin**	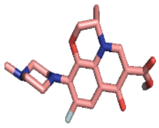	Val206, (H- Bond)<br>Ser141, (H- Bond)<br>Ala208, (H- Bond)	2.89<br>2.04<br>2.95	Met125, (Pi-alkyl)<br>Val206, (Pi-alkyl)<br>Leu113, (Pi-alkyl)<br>Pro188, (Carbon H bond)	5.43<br>4.29<br>4.63<br>3.40	3	7	**-6.30**

Table S7: interactions of ligands with amino acids of Neuraminidase of S. typhimurium (PDB:ID 2SIL)

No	Protein	Ligand	3D Structure	Hydrophilic Interactions		Hydrophobic Contacts		No. of H-Bonds	No. of Total Bonds	affinity kcal mol^{-1}
				Residue (H- Bond)	Length	Residue (Bond type)	Length			
1	**Neuraminidase of S. typhimurium (PDB:ID 2SIL)**	**4c**	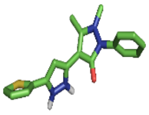	Trp128, (H-Bond)<br>Thr127, (H-Bond)	2.57<br>2.65	Asp100, (Carbon H bond)<br>Met99, (Pi-alkyl)<br>Trp128, (Pi-alkyl)<br>Tyr307, (Sulfur)<br>Asp62, (Pi-Cation)<br>Arg246, (Pi-cation)	3.60<br>4.94<br>4.70<br>4.85<br>4.85<br>3.74	**2**	**8**	**-7.50**
2		**5c**	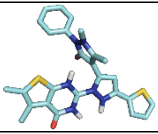	Gln63, (H-Bond)<br>Thr127, (H-Bond)	1.99<br>2.42	Met99, (Sulfur)<br>Met99, (Pi-alkyl)<br>Leu205, (Pi-alkyl)<br>Asp62, (Pi-cation)<br>(Pi-cation) Arg246,<br>Glu231, (Carbon H bond)	5.65<br>5.32<br>4.96<br>4.03<br>4.38<br>3.24	**2**	**8**	**-7.60**
3		**12**	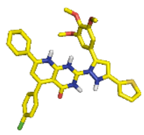	Arg246, (H-Bond)	2.51	Leu205, (Pi-alkyl)<br>Val196, (Pi-alkyl)<br>Leu175, (Pi-alkyl)<br>Met99, (Pi-alkyl)<br>Leu205, (Pi-Sigma)<br>Glu231, (Pi-cation)<br>Asp62, (Pi-cation)<br>Asp100, (Pi-cation)	3.28<br>2.91<br>4.93<br>4.92<br>3.80<br>4.43<br>2.48<br>4.49	**1**	**9**	**-6.90**
4		**5b**	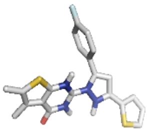	-	-	Tyr307, (Sulfur)<br>Leu175, (Pi-alkyl)<br>Met99, (Pi-alkyl)<br>Glu231, (Pi-cation)<br>(Pi-cation) Arg246,<br>Trp128, (Amide-Pi stacked)<br>Glu129, (Carbon H bond)<br>Leu205, (Pi-Sigma)	5.93<br>4.17<br>4.50<br>4.83<br>3.73<br>4.29<br>3.04<br>3.98	**0**	**8**	**-6.90**
5		**Levofloxacin**	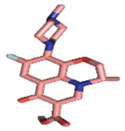	Arg37, (H-Bond)<br>Arg246, (H-Bond)<br>Arg309, (H-Bond)<br>Trp128, (H-Bond)	2.26<br>1.76<br>2.44<br>2.73	Leu205, (Pi-alkyl)<br>Trp128, (Pi-alkyl)<br>Arg246, (Pi-cation)	5.17<br>4.68<br>3.93	**4**	**7**	**-6.20**

Table S8: Prediction of pharmacokinetics and physicochemical properties of compound

Id	ID	4c	5c	12	11	Id	ID	4c	5c	12	11
Physicochemical Properties	MW	336.10	514.12	647.14	494.0	Metabolism	CYP1A2-inh	0.026	0.002	1.000	1.000
	Vol	333.217	493.218	630.429	440.984		CYP1A2-sub	0.985	1.000	0.740	0.999
	Dense	1.009	1.042	1.027	1.120		CYP2C19-inh	0.002	0.001	0.997	1.000
	nHA	5.000	8.000	9.000	5.000		CYP2C19-sub	0.014	0.922	0.000	0.145
	nHD	1.000	1.000	1.000	0.000		CYP2C9-inh	0.020	0.972	0.989	0.992
	TPSA	55.610	90.500	104.150	45.510		CYP2C9-sub	0.641	0.986	0.565	1.000
	nRot	3.000	4.000	8.000	6.000		CYP2D6-inh	0.000	0.000	0.000	0.134
	nRing	4.000	6.000	7.000	4.000		CYP2D6-sub	0.459	0.986	0.934	0.997
	MaxRing	6.000	9.000	10.000	6.000		CYP3A4-inh	0.765	0.852	0.899	0.989
	nHet	6.000	10.000	11.000	9.000		CYP3A4-sub	1.000	1.000	0.581	1.000
	fear	0.000	0.000	0.000	0.000	Excretion	CL (Clearance)	3.6532	2.1942	2.5870	5.2398
	nRig	22.000	33.000	40.000	22.000		T12	0.6391	0.6046	0.8339	0.7406
	Flex	0.136	0.121	0.200	0.273	Toxicity	hERG Blockers	0.1804	0.2164	0.9348	0.6467
	nStereo	0.000	0.000	0.000	0.000		H-HT	0.7505	0.9184	0.9164	0.8299
Solubility	LogS	-3.631	-4.397	-6.080	-7.000		DILI	0.9980	0.9999	0.9998	0.9996
	LogD	2.118	2.713	4.219	4.664		AMES Toxicity	0.7485	0.8122	0.3837	0.3737
	LogP	1.931	2.769	5.660	5.411		Rat OralToxicity	0.3495	0.3310	0.3451	0.4069
	ESOL Log S	-4.47	-6.53	-8.50	-7.28		FDAMDD	0.4151	0.3894	0.8699	0.6845
	Ali Log S	-4.77	-7.82	-9.83	-8.08		Skin Sensitization	0.2765	0.1909	0.0498	0.3309
	Silicon-IT class	Moderately	Moderately	Moderately	Moderately		Carcinogenicity	0.7547	0.8995	0.4839	0.6539
drug-likeness	Lipinski Rule	Accepted	Accepted	Rejected	Accepted		Eye Corrosion	0.0068	0.0000	0.0000	0.0001
	Pfizer Rule	Accepted	Accepted	Accepted	Rejected		Eye Irritation	0.7489	0.0479	0.0024	0.0513
	Golden Triangle	Accepted	Rejected	Rejected	Accepted		Respiratory Toxicity	0.9576	0.9281	0.9343	0.9738
Absorption	Pgp-inh	0.00183	0.00684	0.61268	0.6212	Toxicophoric Rules	Non-Genotoxic Carcinogenicity	1	2	1	
	Pgp-sub	0.03974	0.00109	0.00017	0.00025		LD50_oral	4.0353	4.5125	4.8905	4.6455
	HIA	0.00000	0.00000	0.00001	0.00000		Neurotoxicity-DI	0.8442	0.8556	0.9854	0.9043
	F (20%)	0.00001	0.00000	0.01111	0.00132		Ototoxicity	0.5316	0.8225	0.9058	0.7806
	F (30%)	0.00000	0.00000	0.00134	0.00796		Hematotoxicity	0.5130	0.5821	0.6462	0.5648
	Caco-2	-4.77	-4.76	-4.90	-4.96		Nephrotoxicity-DI	0.7066	0.8984	0.9842	0.8728
	MDCK	-4.47	-4.66	-4.68	-4.77		Genotoxicity	0.9994	1.0000	0.9982	0.9595
Distribution	BBB	0.540	0.430	0.324	0.420	Medicinal Chemistry	RPMI-8226	0.0298	0.0436	0.1148	0.1268
	PPB%	97.079	98.087	99.324	98.436		QED	0.621	0.359	0.179	0.282
	VDss	-0.287	0.074	0.156	0.117		Synth	2.573	3.022	2.968	2.519
	Fu %	2.112	1.525	0.406	1.230		Fsp3	0.111	0.154	0.086	0.136

Table S9: Prediction of toxicity risks and oral toxicity prediction results of compounds

No	Ligand	Toxicity risks				Physicochemical properties					
		**Mutagenic**	**Tumorigenic**	**Irritant**	**Reproductive**	**CLogP**	**Solubility**	**Molecular Weight**	**TPSA**	**Drug likeness**	**Drug score**
1	**4c**	(-)	(-)	(-)	(-)	1.56	-2.74	336.0	80.47	6.94	0.53
2	**5c**	(+)	(-)	(-)	(-)	3.76	-6.00	514.0	139.3	6.79	0.25
3	**12**	(-)	(-)	(-)	(-)	7.69	-10.4	647.0	128.1	9.41	0.15
4	**11**	(+)	(-)	(+)	(+)	5.96	-7.09	494.0	73.75	6.23	0.10

Table S10: Results of common feature pharmacophore models

Hypothesis	Features	Score	Conf/#Mol	Radius
**F1**	Hyd | Aro	100%	4/4	1.48
**F2**	ML | Aro	100%	4/4	1.48
**F3**	ML | Aro | Acc | Don	100%	4/4	1.76
**F4**	ML | Acc | Don	75%	3/3	0.96
**F5**	Aro	75%	3/3	1.15
**F6**	ML | Hyd | Acc	75%	3/3	1.31
**F7**	Acc& ML	50%	2/2	0.53
**F8**	Hyd	50%	2/2	0.54
**F9**	Acc& ML	50%	2/2	0.54
**F10**	Acc& ML	50%	2/2	0.56
**F11**	Hyd	50%	2/2	0.58
**F12**	Hyd	50%	2/2	0.58
**F13**	Acc& ML	50%	2/2	0.58
**F14**	ML	50%	2/2	0.71
